# The sequence of rice chromosomes 11 and 12, rich in disease resistance genes and recent gene duplications

**DOI:** 10.1186/1741-7007-3-20

**Published:** 2005-09-27

**Authors:** The Rice Chromosomes 11 and 12 Sequencing Consortia*

**Affiliations:** 1 Waksman Institute, Rutgers University, Piscataway, New Jersey 08854

## Abstract

**Background:**

Rice is an important staple food and, with the smallest cereal genome, serves as a reference species for studies on the evolution of cereals and other grasses. Therefore, decoding its entire genome will be a prerequisite for applied and basic research on this species and all other cereals.

**Results:**

We have determined and analyzed the complete sequences of two of its chromosomes, 11 and 12, which total 55.9 Mb (14.3% of the entire genome length), based on a set of overlapping clones. A total of 5,993 non-transposable element related genes are present on these chromosomes. Among them are 289 disease resistance-like and 28 defense-response genes, a higher proportion of these categories than on any other rice chromosome. A three-Mb segment on both chromosomes resulted from a duplication 7.7 million years ago (mya), the most recent large-scale duplication in the rice genome. Paralogous gene copies within this segmental duplication can be aligned with genomic assemblies from sorghum and maize. Although these gene copies are preserved on both chromosomes, their expression patterns have diverged. When the gene order of rice chromosomes 11 and 12 was compared to wheat gene loci, significant synteny between these orthologous regions was detected, illustrating the presence of conserved genes alternating with recently evolved genes.

**Conclusion:**

Because the resistance and defense response genes, enriched on these chromosomes relative to the whole genome, also occur in clusters, they provide a preferred target for breeding durable disease resistance in rice and the isolation of their allelic variants. The recent duplication of a large chromosomal segment coupled with the high density of disease resistance gene clusters makes this the most recently evolved part of the rice genome. Based on syntenic alignments of these chromosomes, rice chromosome 11 and 12 do not appear to have resulted from a single whole-genome duplication event as previously suggested.

## Background

Rice (*Oryza sativa*) is a major staple food and is consumed by nearly half the world's population. It accounts for more than 21% of global human per capita energy and 15% of per capita protein. In the past few decades, although rice production has doubled due to the introduction of high yielding varieties/hybrids and improved cultivation practices, it is still insufficient to cope with ever-increasing global demand, which is expected to increase at the rate of about 1% per annum [1]. At the same time, inappropriate natural resource use, along with biotic and abiotic stress pressure, is casting a shadow on rice production [2]. Access to the rice genome sequence will enable identification of genes responsible for traits and alleles that will be essential to meet the growing demands of food production in the coming years. Towards this end, whole-genome shotgun-based draft sequences of the *indica *and *japonica *subspecies of rice were reported previously [3-5] while the International Rice Genome Sequencing Project (IRGSP), using a clone-by-clone approach, focused on generating a high quality, finished sequence of the rice genome [6]. Indeed, access to the rice genome has served as a catalyst for investigations on comparative genomics, functional genomics, map-based gene cloning and molecular breeding in rice [7,8].

Rice is one of the cereals in the Poaceae family, which collectively provides the largest source of calories for human consumption. Within the cereals are larger genome species such as maize, wheat, millet and sorghum [9]. Synteny in the cereals has been reported previously using molecular markers [10] and recently at a higher resolution using sequences available from the rice, sorghum, maize, and wheat genomes [3,11,14]. Thus, access to a complete, high-resolution rice genome sequence will facilitate research on other cereals with larger, partially sequenced genomes.

One of the emerging features of plant genomes appears to be the recent generation of gene copies that have diverged in regulation and function [15]. A common pathway for such a mechanism in plants is whole-genome duplication (WGD), which occurred for instance in maize as little as 5 million years ago [16]. Other mechanisms involve tandem gene amplifications and segmental duplications. In rice, chromosomes 11 and 12 provide such an example. In addition, several genes of agronomic importance, such as blast, bacterial blight, virus and insect resistance, photoperiod-sensitive male-sterility as well as salt tolerance, have been mapped on these two chromosomes [17]. Here, we report the in-depth analysis of chromosomes 11 and 12 of a *japonica *cultivar of rice with genetic lengths estimated to be 118.6 cM and 110.1 cM, respectively [18]. We have generated high-quality finished sequences for these two chromosomes, annotated the chromosomes for genes and other features, and used these data to examine novel features such as the organization of disease resistance genes. We also asked whether chromosomes 11 and 12 are likely to have resulted from a WGD event by examining duplication events between the two chromosomes through alignments of mapped genes of wheat along the rice chromosomes.

## Results and discussion

### General features of chromosomes 11 and 12

**Figure 1:**
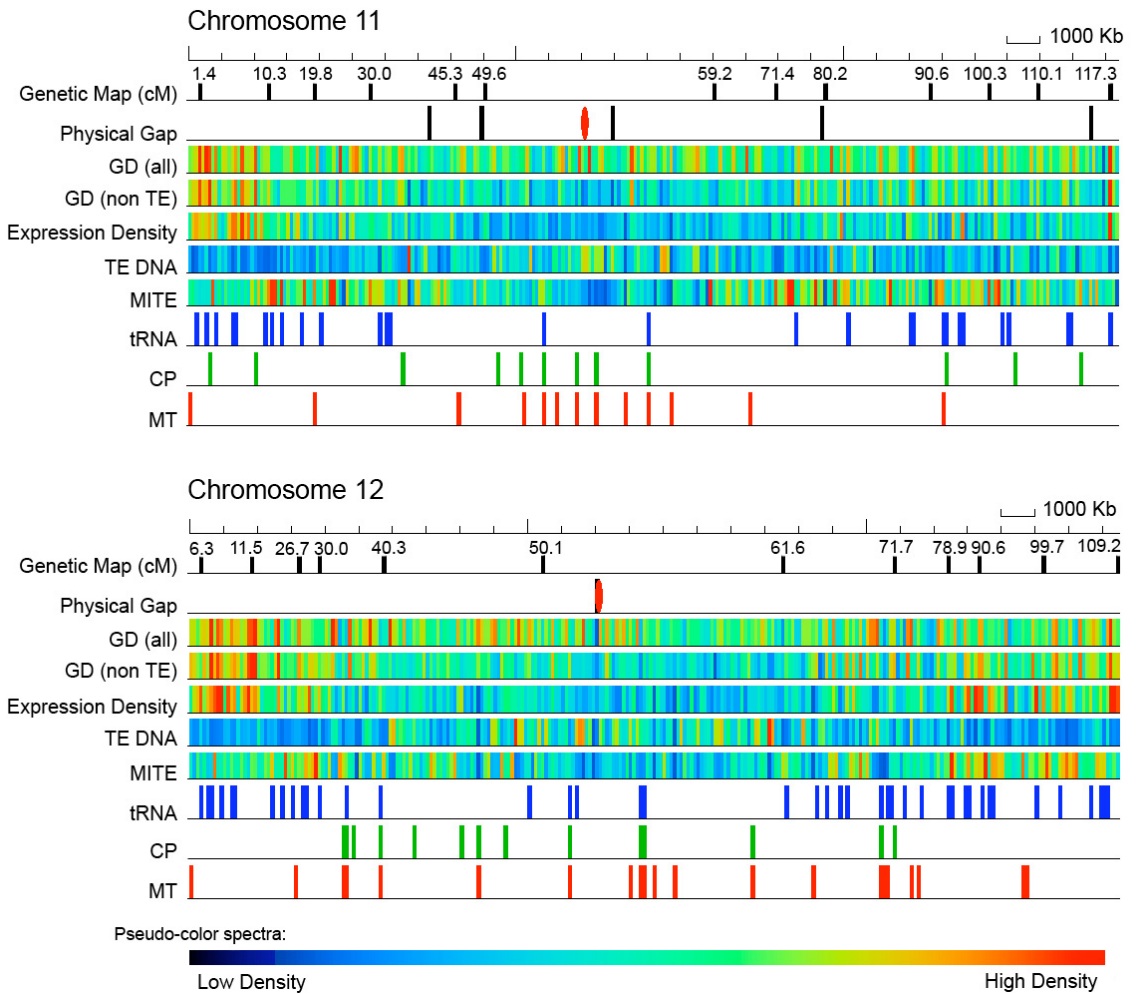
Display of features on rice chromosomes 11 and 12. Using a false-color display, we plotted features present on rice chromosomes 11 and 12. Select genetic markers are plotted with their cM positions noted; physical gaps are plotted with the centromere gap noted in red; gene density (GD) is plotted in two tracks (all genes and non-TE-related genes); expression density is determined by aligment to ESTs; transposable element (TE) density is plotted with a separate track for MITEs; tRNAs are the transfer RNAs, and the CP and MT represent chloroplast and mitochondrial insertions.

A total of 255 and 269 BAC/PAC clones were sequenced from chromosomes 11 and 12, respectively. Chromosome 11 is slightly longer than chromosome 12, with 28.4 Mb and 27.5 Mb, respectively, of sequence identified for them (Table 1, Fig. 1). Excluding the telomeres, a few physical gaps remain on the two pseudomolecules; six and one on chromosomes 11 and 12, respectively (Accession Numbers: chromosome 11: DP000010, chromosome 12: DP000011). This was due to a difficulty in obtaining clones that span these regions, especially the centromeres and telomeres, which present technical challenges in sequencing highly repetitive tracts of the genome.

**Table 1:** Statistics of rice chromosomes 11 and 12

Feature	Statistics	
	Chr11	Chr12
Total number of BACs/PACs	255	269
Total BAC length (Mb)	35.5	33.5
Total nonoverlapping sequence (Mb)	28.4	27.5
Short arm (Mb)	12.0	12.0
Long arm (Mb)	16.4	15.5
Integrated genetic markers	283(loci)	269(loci)
G+C Content		
Overall	42.8	43.0
Exons	51.6	51.8
Introns	38.7	38.8
Intergenic regions	40.8	41.0
Total number of genes^a^	4,286(3,148)	4,169(2,845)
Known/Putative genes	2,364(55.2%)	2,395(57.4%)
Expressed genes	328(7.7%)	336(8.1%)
Hypothetical genes	1,594(37.2)	1,438(34.5%)
Gene density (kb)^b^	6.6(9.0)	6.6(9.7)
Average gene length (bp)^c^	2,603	2,589
Total number of gene models	4,436	4,355
Average exon size (bp)	350	339
Average intron size (bp)	378	371
Average number exons per gene model	4.1	4.2

^a ^Total number of genes includes the TE-related genes and the non-TE-related genes.

^b ^Gene density is reported for all genes and non-TE-related genes (in parentheses).

^c ^Average length of the genes is determined using the length of from the start codon to stop codon of the longest isoform.

Of the 4,286 and 4,169 genes identified on chromosomes 11 and 12, respectively, 3,148 and 2,845 could be annotated as non-transposable element related (non-TE; Fig. 1). A similar percentage of genes on the two chromosomes could be assigned a putative function or annotated as encoding an expressed protein, leaving a similar percentage annotated as encoding a hypothetical protein (Table 1). With respect to domain composition, there was a striking difference in representation of some Pfam domains between the chromosomes, suggesting the presence of genes coding for different suites of proteins on the two chromosomes. As shown in Tables 2 and 3, there was a large enrichment of proteins on chromosome 11 (166 proteins) containing a leucine-rich repeat (LRR) domain, which is involved in protein-protein recognition and is a hallmark of disease resistance genes (see below). Besides the LRR-domain, there are two other domains common to disease resistance genes, protein kinase and NB-ARC domains, which were also enriched on chromosome 11 relative to chromosome 12; 106 versus 74 proteins containing protein kinase domains and 102 versus 49 proteins with NB-ARC domains on chromosome 11 versus 12, respectively. Based on alignments by a rice transcript, 1,235 (39%) and 1,221 (43%) of non-TE-related genes on chromosomes 11 and 12, respectively, were active (Additional figure 10 [see Additional file 1]). Of these, 952 and 980 non-TE-related genes from chromosomes 11 and 12, respectively, could be aligned with a FL-cDNA. In addition, 152 non-TE-related genes aligned with ESTs from other monocots on each chromosome. For 26 genes from chromosome 11 and 23 from chromosome 12, only non-monocot evidence was available for expression (Additional figure 10 [see Additional file 1]). A ready set of mutants is available for 800 and 845 genes on chromosomes 11 and 12, respectively, as evidenced by a flanking sequence tag (FST) in or within 500 bp of the transcription unit.

**Table 2:** Predominant Pfam domains within the chromosome 11 predicted rice proteome. All non-TE related proteins were searched using the Hmmpfam program and Pfam domains above the trusted cutoff were parsed out. Only the top 20 Pfam domains are listed.

**Pfam accession**	**Numbers of matched proteins**	**Pfam common name**
PF00560	166	Leucine Rich Repeat
PF00069	106	Protein kinase domain
PF00931	102	NB-ARC domain
PF00646	78	F-box domain
PF00023	32	Ankyrin repeat
PF00097	29	Zinc finger, C3HC4 type (RING finger)
PF01535	23	PPR repeat
PF00098	21	Zinc knuckle
PF05699	19	*h*AT family dimerisation domain
PF06654	19	Protein of unknown function (DUF1165)
PF07197	19	Protein of unknown function (DUF1409)
PF04578	14	Protein of unknown function, DUF594
PF00036	13	EF hand
PF00076	13	RNA recognition motif. (a.k.a. RRM, RBD, or RNP domain)
PF03018	13	Dirigent-like protein
PF00067	12	Cytochrome P450
PF00400	12	WD domain, G-beta repeat
PF00651	12	BTB/POZ domain
PF00704	12	glycosyl hydrolase, family 18
PF00106	11	oxidoreductase, short chain dehydrogenase/reductase family

**Table 3:** Predominant Pfam domains within the chromosome 12 predicted rice proteome. All non-TE related proteins were searched using the Hmmpfam program and Pfam domains above the trusted cutoff were parsed out. Only the top 20 Pfam domains are listed.

**Pfam accession**	**Numbers of matched proteins**	**Pfam common name**
PF00069	74	Protein kinase domain
PF00560	60	Leucine Rich Repeat
PF00646	51	F-box domain
PF00931	49	NB-ARC domain
PF00098	28	Zinc knuckle
PF01535	28	PPR repeat
PF03578	27	HGWP repeat
PF00097	25	Zinc finger, C3HC4 type (RING finger)
PF05699	22	hAT family dimerisation domain
PF00036	18	EF hand
PF00249	16	Myb-like DNA-binding domain
PF00067	14	Cytochrome P450
PF00076	13	RNA recognition motif. (a.k.a. RRM, RBD, or RNP domain)
PF00004	11	ATPase, AAA family
PF00010	11	Helix-loop-helix DNA-binding domain
PF00400	11	WD domain, G-beta repeat
PF02892	11	BED zinc finger
PF06654	11	Protein of unknown function (DUF1165)
PF07197	11	Protein of unknown function (DUF1409)
PF00023	10	Ankyrin repeat

A similar fraction of chromosomes 11 and 12 could be classified as repetitive (29.5% and 31.6%, respectively (Additional tables 5 and 6 [see Additional file 1]), which with the exception of miniature inverted repeat transposable elements (MITEs) were enriched in the centromeric and pericentromeric regions of the two chromosomes (Fig. 1). As noted previously [19], MITEs, which constitute 18.6 and 16.2% of the repetitive sequences on chromosomes 11 and 12, respectively, were enriched in the euchromatic arms of the two chromosomes (Fig. 1).

**Figure 2:**
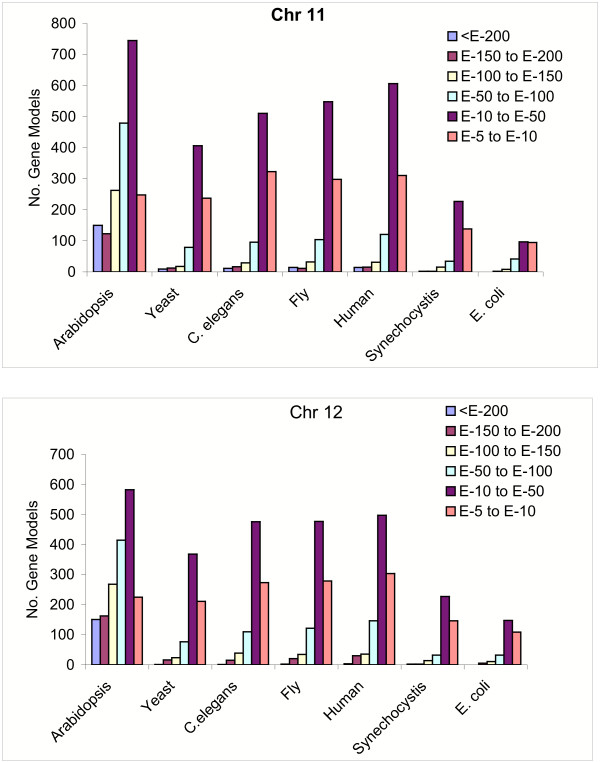
Presence of potential homologs in rice chromosomes 11 and 12 with other model organisms. BLASTP was used to search the proteomes of the listed model organisms and with the non-TE-related proteins from chromosomes 11 and 12. The number of proteins with matches at the designated E-value cutoffs are plotted for each model organism.

A similar number of non-TE-related proteins with putative homologs in model species were present in chromosomes 11 and 12 (Fig. 2). The highest number of putative homologs was seen in Arabidopsis, with 60.9% and 59.6% of the proteins from chromosomes 11 and 12, respectively, having a potential homolog in Arabidopsis (E value cutoff 10^-5^). Similar ranges of potential homologs were seen between the two chromosomes and the other model organisms, ranging from 7–30.6% in chromosome 11 and 10–33.6% in chromosome 12.

A high percentage of rice genes could be aligned with genomic assemblies from two other cereals, maize and sorghum. Of the 2,405 maize assemblies with a best hit on rice chromosome 11 or 12, 1,879 overlap with non-TE-related genes while 160 overlap with TE-related genes (Additional table 7 [see Additional file 1]). The remaining 366 assemblies align to intergenic regions, which might include genes or TEs that are degenerated and not recognized by our bioinformatics methods. Interestingly, for sorghum, the occurrence of alignments was similar to maize except that there was a higher incidence of alignment to TE or intergenic regions, which could be due the fact that the sorghum dataset includes only methyl-filtrated and not C_o_*t*-enriched sequences [20-22]. The 1,469 non-TE-related genes that aligned to both maize and sorghum genomic assemblies suggest that these genes might predate the divergence of the Panicoideae, estimated at 50 mya [23].

### Disease resistance genes in rice chromosomes 11 and 12

**Figure 3:**
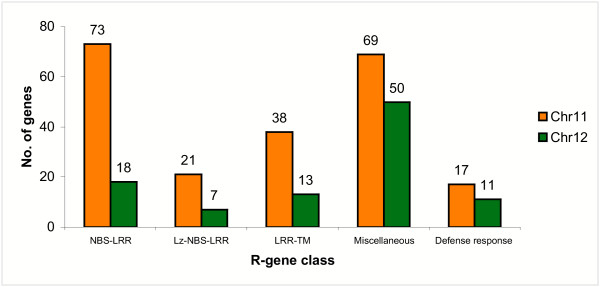
Frequency distribution of different categories of R-like genes and defense response genes on rice chromosomes 11 and 12. The miscellaneous category includes genes showing high homology with putative disease resistance proteins and defense response category includes genes showing high homology to chitinases, glucanases and thaumatin-like proteins. (LZ, leucine zipper; NBS, nucleotide binding site; LRR, leucine rich repeat; TM, trans-membrane).

**Figure 4:**
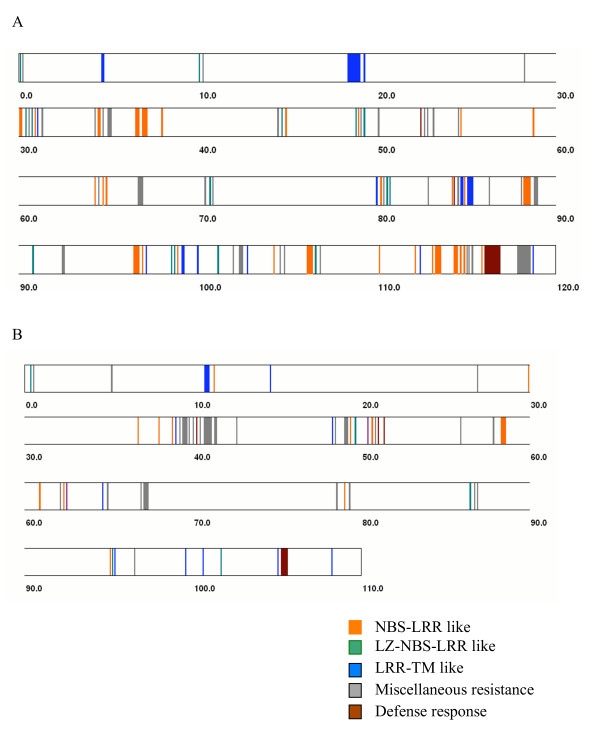
Distribution pattern of resistance genes and defense response genes on rice chromosomes 11 (A) and 12 (B). Each gene category is color coded and plotted on the rice chromosome bar with respect to its cM position. Width of the vertical colored bars is proportional to the number of genes located at that position.

**Figure 5:**
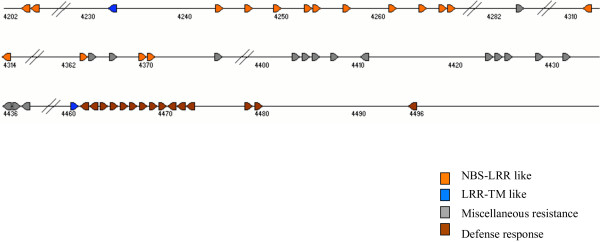
Plot of a portion of the rice chromosome 11 showing tandem arrays of disease resistance and defense response genes between positions 112.0 – 119.0 cM. The category of genes is color-coded and the arrowheads depict their direction. The numbers indicate cumulative number of all the genes predicted by TIGR on chromosome 11. The scale is based on number of genes such that the space occupied by one arrowhead corresponds to one gene, genes in the gap between arrowheads do not match with R-like genes and large gaps of unmatched genes are marked by a double slash (//).

Disease resistance genes (R-like genes) conferring resistance to viral, bacterial, fungal and nematode pathogens have been grouped into five classes on the basis of their encoded protein products [24]. For instance, a genome-wide study identified such typical protein domains in the R-like genes of Arabidopsis [25]. Major classes of conserved domains include leucine zipper (LZ), coiled coil (CC), nucleotide binding site (NBS), leucine-rich repeat (LRR), protein kinase, Toll-IL-IR homology region (TIR) and trans-membrane (TM) as well as miscellaneous R-genes. Apart from the genes containing these typical domains, one maize disease resistance gene, HM1, encodes a reductase that detoxifies HC-toxin [26]. We analyzed the sequences of chromosomes 11 and 12 to identify the type and distribution of the disease resistance as well as downstream defense response genes (Fig. 3). We identified 201 R-like gene models in chromosome 11 (Additional table 8 [see Additional file 1]), which is 4.5% of the total number of gene models predicted for this chromosome. Of these, 73 (36.3%) have homology to the NBS-LRR class of R-like genes and 38 show homology to the LRR-TM-like genes. The long arm of rice chromosome 11 (11L) contains almost twice (132 genes) the number of R-like genes compared to the short arm (68 genes). We also identified 17 downstream defense response genes including glucanases, chitinases and thaumatin-like proteins. Most of these R-like genes and defense response-like genes are present in large clusters of tandem arrays indicating their origin by duplication from a few ancestral genes (Fig. 4). Large clusters of genes (31, 35 and 37 members) are present between positions 30–40 cM (between 5,658,160 – 7,920,402 bp), 80–90 cM (between 20,001,952 – 22,632,644 bp) and 110–119 cM (between 26,213,144 – 28,180,239 bp) of chromosome 11, respectively. Nucleotide positions are based on the pseudomolecules of TIGR release 3 [27]. A detailed depiction of duplicated genes in the interval 112–119 cM on chromosome 11 is shown in Fig. 5 and a cladogram of these genes is shown in Additional figure 11 [see Additional file 1], confirming that the genes within a cluster generally belong to the same clade. A tandem array of 12 *Xa21*-like genes is present on the short arm of chromosome 11 at position 19 cM (between 3,516,492 – 3,665,074 bp), while the actual *Xa21 *gene is present on the long arm of chromosome 11 [28]. A large cluster of 14 defense response genes, 12 of which are chitinases, is present in tandem at 116.2 cM (between 28,056,455 – 28,122,601 bp). Several other R-like genes are also arranged in similar but small clusters.

**Figure 6:**
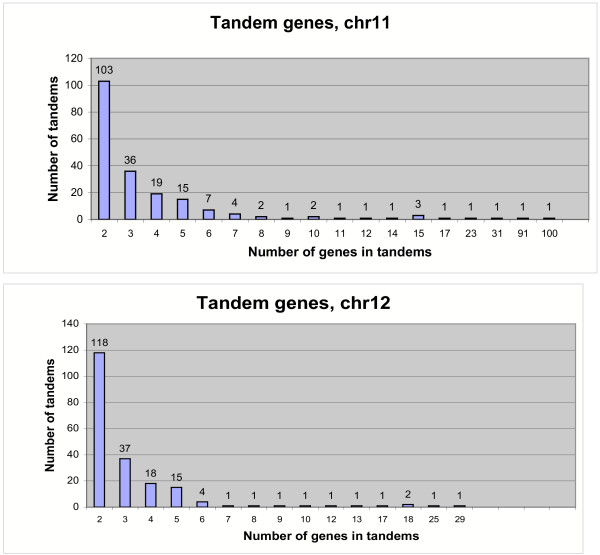
Array sizes of tandemly repeated genes on rice chromosomes 11 and 12. Total tandemly amplified genes on 11 are 924 or 29% of the total genes; total tandemly amplified genes on 12 are 684 or 24% of the total genes.

On chromosome 12, 88 gene models showed homology to R-like genes (Fig. 3 Additional table 8 [see Additional file 1]), which is 2.0% of the total number of gene models predicted for this chromosome. However, 50 of these 88 gene models belonged to the miscellaneous category including viral resistance, Verticillium wilt resistance, BLB resistance genes and those containing LRR motif but without NBS, CC or LZ motifs. On chromosome 12, only 18 gene models showed homology to the NBS-LRR category. Although the R-like genes and defense response genes were distributed throughout the chromosome, they were present in clusters (Fig. 4). A large cluster of 23 miscellaneous resistance genes was found between positions 40–50 cM (between 5,858,249 and 10,009,727 bp) and a cluster of seven defense response genes was found at position 107.4 cM (between 26,803,729 and 26,870,016 bp). The previously described blast resistance gene *Pita *[29] was present near the centromere of chromosome 12. The total number of R-like genes in chromosome 12 was less than half of the number in chromosome 11, even though the size and total number of genes in these two chromosomes are similar. This can be attributed to the enrichment of NBS-LRR, LZ-NBS-LRR and LRR-TM-like genes in chromosome 11, which was 3–4 times higher than chromosome 12 (Fig. 3). The difference in the number of R-like genes between the two chromosomes also has an impact on the extent of tandem gene arrays. Chromosome 11 has a total of 924 genes (29%) and chromosome 12 has 684 genes (24%) that are duplicated at least once within a short distance (Fig. 6).

A previous report on the analysis of R-like genes in the entire rice genome revealed that most of the R-like genes (24.98%) are present on chromosome 11 [30]. Another report found more than 25% of the physically mapped NBS-encoding genes on chromosome 11 and claimed that approximately 20% of the NBS-LRR genes in the Nipponbare genome were predicted to be pseudogenes [31]. Comparison of the total number of genes containing the LRR domain (837) in the finished genome sequence [6] with the number of such genes on chromosomes 11 (166 genes) and 12 (60 genes) in the present study (Tables 2 and 3) shows a similar percentage (27%) of R-like genes to be on these two chromosomes. However, the previous total estimate of 536 *R*-like genes is significantly lower than the one from the finished genome (837), indicating that previous draft sequences did not provide sufficient information for capturing all candidate genes for disease resistance and, in addition, that gene finding methods are not directly comparable. Complete sequence information is also important for accurate sequence alignments of R-like genes with cloned disease resistance genes from rice (*Pita*, *Xa21*, *Pib*) Arabidopsis (*RPM1*), tomato, (*Cf2*, *Cf9*), barley (*Mla1*) and wheat (*Lr10*). The R-like gene and defense response gene hot spots in these two chromosomes will be invaluable for future mapping and cloning of disease resistance genes from naturally occurring disease resistant rice lines. However, manual re-annotation of these R-like genes, as done in case of Arabidopsis [25], and functional validation of the candidate resistance genes, would be important for drawing practical benefits from this information.

### Recent duplication on chromosomes 11 and 12

**Figure 7:**
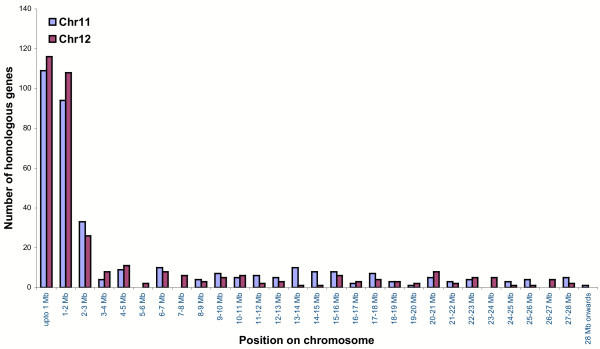
Chromosome 12 sequences were used as query against a database of chromosome 11 sequences using MegaBLAST as described under Methods. At ≥50% coverage and >80% identity, the frequency distribution of unique duplicate gene models is plotted over the length of chromosomes 11 and 12. Based on the chromosomal locations of these unique genes, duplications were identified throughout the length of both the chromosomes. The maximum extent of duplication, however, was found to be confined within the first 2 Mb region of both chromosomes.

**Figure 8:**
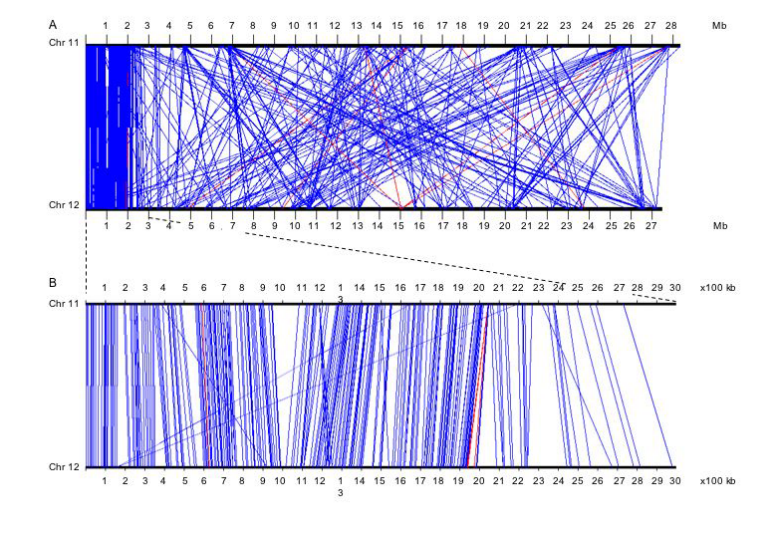
Gene duplication between rice chromosomes 11 and 12 over the whole length (a) and in the first 3 Mb region (b). Blue and red lines connect duplicate gene models in the same and opposite orientation, respectively.

Chromosomes 11 and 12 harbor duplicated regions at the distal ends of their short arms as determined by physical and genetic mapping [32]. Based on previous versions of the rice genome sequence, it became evident that segmental duplications occur throughout the genome [5,7,33]. Here, we focused on segmental duplications between chromosome 11 and 12 only because the finished sequences of these chromosomes permit us to conduct an analysis of the organization of their genes in a detail that was not possible before. At ≥50% coverage and ≥80% identity and excluding TE-related gene models, 546 gene models were identified as duplicated. A subset of these, 350 from chromosome 11 and 352 from chromosome 12, was found to be unique between these chromosomes, excluding repeated gene models. Based on the chromosomal locations of these unique genes, the maximum extent of duplication was confined within the first 3 Mb of both chromosomes (Fig. 7). Possibly, there could be a difference between *japonica *and *indica *varieties, as previous comparisons identified a second duplication between chromosomes 11 and 12 in the *indica *[5] but not the *japonica *[33] species. Here, most of the duplicated gene models (98%) were found in the same orientation on both the chromosomes (Fig. 8a,b). The 3 Mb region was further analyzed to find the number of duplicated genes at ≥30, ≥40, ≥50, ≥60, ≥70, ≥80 and ≥90% coverage. The numbers of duplicated genes at ≥30% coverage for chromosomes 11 and 12 were 287 and 304, while at ≥90% coverage, the numbers of duplicated genes were 109 and 113, respectively (Additional table 9 [see Additional file 1]). Using alignment to ESTs and FL-cDNAs, a total of 132 (chromosome 11) and 145 (chromosome 12) gene models were expressed; of these, a total of 90 expressed gene models were common to both chromosomes. Interestingly, 42 and 55 gene models were expressed but their homoeologous copies on the respective chromosome were not expressed [Additional table 10; see Additional file 1]. Although it is possible that all ESTs or cDNAs are not represented in the database, it may be that variation in expression reflects diverged expression of one of the homoeologous copies. Divergence of regulation of gene expression after gene amplification has also been reported in maize [15,34].

To determine the time of the segmental duplication, the full-length coding regions of a subset of homoeologous genes that had been genetically mapped and were distributed over the entire length of the duplication were aligned and pair-wise analyzed for their nucleotide substitution rates (Table 4). By applying a codon likelihood model, synonymous (Ks) and non-synonymous (Ka) substitution rates were compared. The values for each gene pair varied 18-fold for Ks and 556-fold for Ka, indicating that divergence rates differed significantly among genes. We therefore applied the χ^2 ^homogeneity test to the estimates of divergence time for the rice homoeologs [16]. The χ^2 ^value was highly significant (χ^2 ^= 191.2, P < 0.001), implying that these linked genes diverged within the same time interval. Based on the speciation of rice about 50 mya [23], we calculate the average divergence time for the segmental duplication to be 7.7 mya. We explain the difference from previously published estimates of 25 and 21 mya [3,5] by the sequence accuracy of finished versus whole-genome shotgun sequences [6].

**Table 4:** Nucleotide substitution analysis of a subset of genes in the duplication of chromosomes 11 and 12. A high confidence gene set has been selected for the calculation of synonymous (Ks) and non-synonymous (Ka) nucleotide substitutions. The genes are presented with their Ks/Ka values according to their chromosomal positions.

Gene Number	gene putative function	chrom11 start	chrom11 stop	chrom12 start	chrom12 stop	Ks	se of Ks	Ka	se of Ka	my
1	WRKY family transcription factor	749015	750212	788607	789687	0.16	0.037	0.055	0.013	12.30769
2	WRKY family transcription factor	758715	762245	801426	804997	0.04	0.017	0.015	0.006	3.076923
3	WRKY family transcription factor	781473	782513	816140	817037	0.023	0.017	0.007	0.004	1.769231
4	WRKY family transcription factor	786625	787866	823302	824540	0.077	0.02	0.04	0.007	5.923077
5	alpha-hydroxynitrile lyase	811241	812335	851943	854138	0.0196	0.034	0.0118	0.007	1.507692
6	polyneuridine aldehyde esterase-like	812960	814118	855275	856433	0.025	0.0147	0.009	0.0065	1.923077
7	glycosyl hydrolase family	819185	821808	857627	859498	0.02567	0.009	0.02	0.0053	1.974615
8	scarecrow	1119113	1121579	1042889	1045809	0.0865	0.03	0.0087	0.0026	6.653846
9	glutathione S = transferase T3	1165521	1166165	1096622	1097382	0.327	0.116	0.155	0.04	25.15385
10	apyrase GS52	1174941	1177791	1106432	1109216	0.027	0.01	0.019	0.005	2.076923
11	apyrase S-type	1221864	1225463	1129908	1132441	0.084	0.04	0.027	0.016	6.461538
12	no apical meristem	1233158	1234682	1138382	1139934	0.094	0.026	0.017	0.004	7.230769
13	no apical meristem	1240175	1241314	1146147	1147453	0.32	0.15	0.073	0.0155	24.61538
14	40S robosomal protein S16	1284724	1285173	1168928	1169349	0.093	0	0.003	0	7.153846
15	exostosin family	1288082	1291244	1172080	1174695	0.098	0.02	0.0135	0.004	7.538462
16	cdc45-like protein	1305369	1307183	1188143	1189957	0.068	0.02	0.0037	0.0017	5.230769
17	myb protein homolog	1316546	1317575	1199788	1200856	0.0535	0	0.0115	0	4.115385
18	homoserine dehydrogenase-like protein	1328599	1332331	1215892	1219509	0.0339	0.01	0.01	0.0037	2.607692
19	receptor-like protein kinase	1537568	1540056	1472686	1475196	0.1	0.036	0.02	0.0054	7.692308
20	receptor-loke protein kinase	1545094	1547508	1490358	1492844	0.081	0.021	0.011	0.0027	6.230769
21	60S acidic ribosomal protein	1645643	1647855	1601129	1603328	0.071	0.019	0.0054	0.0027	5.461538
22	calcium dependent protein kinase	1691610	1693844	1647299	1649413	0.0279	0.01	0.00077	0.00078	2.146154
23	YVH 1 protein-tyrosine phosphatase	1696442	1698793	1651813	1654064	0.034	0.012	0.012	0.00436	2.615385
24	hydroxymethylglutaryl-CoA lyase	1715582	1718088	1671209	1673936	0.0757	0.016	0.022	0.0053	5.823077
25	cytochrome P450-like protein	1766039	1767592	1715187	1716737	0.176	0.039	0.0138	0.003	13.53846
26	zinc finger protein-like	1791802	1794503	1731264	1733959	0.1596	0.0386	0.0132	0.0039	12.27692
27	cell death associated protein	1802302	1804168	1744300	1746170	0.1235	0.036	0.0204	0.00572	9.5
28	steroid sulfotransferase	1911460	1912476	1836949	1837788	0.1936	0.078	0.202	0.049	14.89231
29	cyt p450 protein	1997769	2000557	1905242	1908061	0.02	0.015	0.0088	0.0006	1.538462

The number of unique duplicated gene models for the remaining portion (~24–25 Mb) of chromosomes 11 and 12 was 114 (32.6%) and 102 (29%), respectively, with a high degree of rearrangement. Within this region, there were 163 and 189 gene models at ≥30% coverage, while at ≥90% coverage, there were only 31 genes for both the chromosomes (Additional table 10 [see Additional file 1]). Interestingly, only 18 (15.8%) and 14 (13.7%) genes (chromosome 11 and 12, respectively) were found to be expressed based on homology to an EST or cDNA, in contrast to 56 and 58% in the first 3 Mb region of both the chromosomes. Out of these, nine expressed genes were common to both chromosomes. Also, there were nine and five expressed genes of chromosomes 11 and 12 that had non-expressed counterparts. This analysis supports the view that the first 3 Mb duplication of chromosome 11 and 12 is of recent origin. However, the loss of order as well as the low percentage of expressed genes, on each chromosome or in duplicate gene pairs, does not support a duplication of the remaining regions of chromosomes 11 and 12, unless it is very ancient in origin and signatures of duplication have largely disappeared.

### Rice-wheat synteny

To investigate further whether rice chromosomes 11 and 12 were related to each other and arose by a WGD event, we compared each gene model predicted from these two chromosomes against 586,577 wheat ESTs. Comparison of bin-mapped wheat ESTs with earlier versions of rice genome sequence provided a glimpse of the structural similarities between these two important cereal genomes [11,13]. However, the present study involves comparative analysis of the finished version of rice chromosomes 11 and 12, which allowed a higher resolution analysis with wheat and a comparative analysis between rice chromosomes 11 and 12. A gene-by-gene comparison of sequence homology of all the predicted rice gene models with the wheat ESTs revealed that 1,588 (35.8%) and 2,220 (51%) of the gene models in chromosomes 11 and 12, respectively, have significant homology with the wheat ESTs at a bit cut-off score of 100. Of these, 416 gene models (26.2%) from chromosome 11 and 552 gene models (24.86%) from chromosome 12 showed significant similarity with bin-mapped wheat EST contigs, a number that is much larger than the levels reported earlier for chromosomes 11 and 12 [13]. A complete list of the matching genes along with their annotated functions is provided in Additional tables 11 and 12 [see Additional file 1].

**Figure 9:**
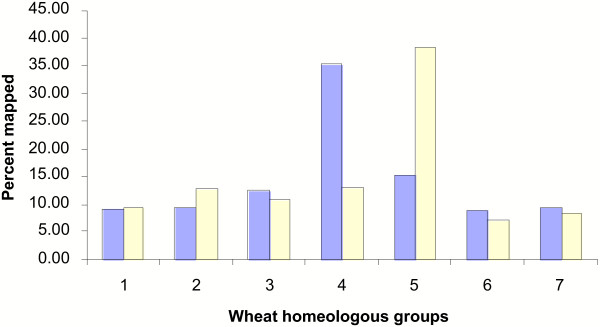
Syntenic mapping of rice genes from rice chromosomes 11 and 12 to wheat. Distribution of the rice gene homologues from rice chromosomes 11 (blue bars) and 12 (yellow bars) on to the wheat chromosomes of seven homoeologous groups.

Out of the 416 gene models from rice chromosome 11, 338 (81.3%) mapped to single wheat homoeologous groups, with the maximum number (35.2%) located on group 4 chromosomes of wheat. The distribution of rice genes that mapped to the wheat chromosome groups 1, 2, 3, 5, 6 and 7 was 9.2%, 9.5%, 12.4%, 15.4%, 8.9% and 9.5%, respectively (Fig. 9 Additional figures 12 and 13 [see Additional file 1]). The majority of rice chromosome 11 gene models mapped to group 4 chromosomes of wheat indicating their common origin. Many of these genes were clustered in the distal region of the long arm of wheat chromosome 4A, but the same gene models mapped to the short arms of wheat chromosomes 4B and 4D, reflecting significant rearrangements and a dynamic state of gene organization in homoeologous chromosomes of wheat and rice chromosome 11. Similar to chromosome 11, a high percentage of chromosome 12 gene models (441, 79.9%) mapped to single wheat homoeologous groups. Of these, the maximum percentage (30.6%) mapped to group 5 chromosomes of wheat whereas the remainder were distributed almost uniformly on the other six wheat homoeologous groups (Fig. 9 Additional figures 12 and 13 [see Additional file 1]).

A comparative distribution of rice gene homologs from chromosomes 11 and 12 to the seven wheat homoeologous groups clearly indicates that the two rice chromosomes have different origins and, apart from the recent 3 Mb duplication, there is not much in common between the two chromosomes. This contradicts earlier observations that the whole of the rice chromosomes 11 and 12 may have evolved as a result of chromosome duplication via polyploidy about 70 mya, i.e. before the divergence of cereals [7]. Furthermore, these results are also consistent with the alignment of the genetic maps of cereal genomes [10], where rice aligns with two homoeologous regions of maize.

## Conclusion

With the completion of the sequences of rice chromosomes 11 and 12, we were able to identify 289 R-like and 28 defense response-like genes, accurately date a 3 Mb recent duplication between the two chromosomes, and show significant synteny between these two chromosomes and wheat chromosome groups 4 and 5, respectively. Rice chromosome 11 has several large clusters of fast evolving disease resistance and defense response genes that have originated by the process of tandem duplication and subsequent divergence under the selective pressure of rice pathogens. This sequence and annotation will be an essential resource for the engineering of rice for tolerance to biotic and abiotic stresses to accommodate growing production needs.

## Methods

### Sequencing and bioinformatic methods

Bacterial artificial chromosome and P1-derived artificial chromosome (PAC) clones from chromosomes 11 and 12 were sequenced to 8–10× coverage using shotgun DNA sequencing [35,36] and standard high-throughput methods [6]. Gaps and ambiguities were resolved using re-sequencing, alternative chemistries, PCR and primer walking [6]. Pseudomolecules or virtual contigs for chromosomes 11 and 12 were constructed as described previously [27]. The pseudomolecules and annotation are available in GenBank under the accession numbers DP000010 and DP000011. Genes were identified using the *ab initio *gene finder FGENESH [37] and gene model structure was improved with EST and full-length cDNA (FL-cDNA) evidence using the Program to Assemble Spliced Alignments [38]. Genes were annotated for function as described [27]. Transposable element-related (TE) genes were identified using either TBLASTN similarity to known repetitive elements in the TIGR *Oryza *Repeat Database [39] or the presence of repetitive element-related Pfam domains [40]. Flanking sequence tags (FSTs) from various insertion mutagenesis projects were aligned with the two pseudomolecules using flast [41] with a cutoff of 95% identity over 80% of the FST length. Transfer RNAs were identified using tRNA-ScanSE [42]. Organellar insertions were determined using BLASTN with the rice mitochondrion and chloroplast genome sequences using a cutoff of 95% identity (Fig. 1). Sequenced genetic markers (4,619 sequences [18,43]) were searched against the pseudomolecules with flast; a cutoff criterion of ≥95% identity over ≥90% length of the marker was used to align the markers to the chromosome sequence. Repetitive sequences were identified, classified, and quantitated on the pseudomolecules using RepeatMasker [44] with the TIGR *Oryza *Repeat Database [39] and a default RepeatMasker cut-off score of 225.

Non-TE-related genes with rice transcript evidence were identified by searching chromosomes 11 and 12 against the TIGR rice gene index (Release 16 [45]). To identify genes with FL-cDNA support, we searched 33,678 FL-cDNAs available from the KOME database [46]. Genes with an alignment of ≥95% identity and a minimum of 50% length of the alignment covered by a gene index sequence were considered supported by a rice EST or FL-cDNA. Seven monocot gene indices [47] were also used to demonstrate support of expressed genes (wheat release 9.0, maize release 15.0, barley release 9.0, sorghum release 8.0, rye release 3.0, onion release 1.0 and sugarcane release 2.1). Cutoff criteria used with the non-rice monocot gene indices were ≥70% identity over 80% of the length of the gene index sequence and a minimum of 50% of the gene model coverage by the gene index sequence. For the analysis of the type and distribution of the disease resistance and defense response genes, coding sequences from the chromosomes 11 and 12 gene models were used in a BLASTX search with the nr database of NCBI [48] and the top hits were extracted in Excel files. The BLASTX output was then searched manually using auto filters against 24 different keywords/phrases known to represent R-like and defense response genes, and categorized into five main classes as follows: (i) NBS-LRR (matching with NBS-LRR, but not with LZ-NBS-LRR and LRR, CC-NBS-LRR, Pib, Pita, I2C, Rp1-d8, T10RGA, LR10, Mla1 and rust resistance), (ii) LZ-NBS-LRR (matching with LZ-NBS-LRR, but not with NBS-LRR, CC-NBS-LRR, LRR and RPM1), (iii) LRR-TM (matching with Xa21, serine/threonine kinases and Cf2/Cf5 resistance), (iv) miscellaneous category (matching with disease resistance, viral resistance, Yr10, Verticillium wilt resistance, LRR, but not with NBS-LRR, CC-NBS-LRR, LZ-NBS-LRR and bacterial leaf blight resistance) and (v) defense response genes (matching with glucanases, chitinases and thaumatin).

Maize genomic assemblies (243,807 total [Release 4.0, Feb. 23, 2004]) were downloaded from TIGR [20,49]. Sorghum genomic reads (593,969) were downloaded from NCBI and processed using the Lucy program [50] to remove vector and low quality sequences. The remaining "good" reads (504,458) were assembled into 163,908 clusters as described [20]. The assemblies from sorghum and maize were searched against the TIGR rice pseudomolecules [Release 3.0, January 2005] using the BLASTZ program [51]. Homologs were identified in other model organisms by searching the non-TE-related proteins from chromosomes 11 and 12 against the *E. coli*, *Synechocystis*, yeast, *Drosophila*, *C. elegans*, human and Arabidopsis predicted proteomes using BLASTP.

Chromosome 11–12 duplication analysis was performed using the predicted gene models. Chromosome 12 sequences were used as query against a database of chromosome 11 sequences using MegaBLAST [52]. Only non-TE gene models at ≥50% coverage were used for further analysis. To compare the expression pattern of duplicated genes, a homology search was performed against the NCBI EST and KOME cDNA databases using the unique duplicated non-TE gene models at ≥90% coverage and identity. Wheat-rice synteny was determined using BLASTN of the chromosome 11 and 12 gene model sequences against *Triticum aestivum *sequences obtained from dbEST. Search parameters were as described previously [12]. Matching gene models were compared with wheat contigs containing bin-mapped ESTs [53] and plotted on the 21 wheat chromosomes.

## List of abbreviations used

bacterial artificial chromosome (BAC), flanking sequence tag (FST), leucine-rich repeat (LRR), million years ago (mya), nucleotide binding site (NBS), resistance gene (*R*-gene), transposable element (TE), whole-genome duplication (WGD).

## Authors' contributions

Consortia members are listed in order of Mb of DNA sequences produced. CRB (TIGR), NKS (IIRGS-IARI), AT (IIRGS-UDSC), and JM (PGIR) conducted the coordination of data analysis and manuscript conception.

At GENOSCOPE, EP, AC, JW, MS, and FQ provided data analysis or intellectual input while NC, ND, GO, SS, AD, LC, BS, PW and CS contributed to sequence/assembly aspects of the project.

At IIRGS-IARI, AD, IAG contributed in physical mapping and identification of BACs; KG, AS, MY, RD, SP, MR, PKS and VS in shotgun cloning, template preparation; SS, AB, AP, KS, HS, SCS and SDM in sequencing, VD and AKP in sequence assembly and annotation, KB in wheat-rice synteny. TRS, TM and NKS contributed in all activities and provided intellectual inputs in analysis.

At IIRGS-UDSC, SR, AM, AKB, AG, VG, DK, VR, SV, AK, PK, SS contributed to sequence, assembly and data analysis. PK, JPK, AKT provided intellectual input for strategic plan and participated in manuscript preparation. AKT is also Coordinator of IIRGS.

At TIGR, QY, SO, JL, WZ, AW, HL, JH, BH, JW, SLS, OW, CF, and CRB provided data analysis or intellectual input while KMJ, MK, LO, TT, DF, JB, BW, SJ, MR, HV, LT, SVA, ML, TU, TF, VZ, SI, JH and ARV contributed to sequence/assembly aspects of the project.

At AGI, WW, DK and CM contributed to shotgun library construction and clone management. YY, TR, JC, KC, HRK, DS and RAW contributed to shotgun sequencing, sequence assembly and finishing aspects of this project.

At CSHL, MK and LS provided sequence assembly and finishing, LN, RP and TZ production sequencing, LP, AO and SD contributed to data analysis and annotation, and WRM directed the research.

At PGIR, EL contributed to clone management, EL and BT to shotgun library construction and sequence/assembly, CD, GF and JM to data analysis, and JM to writing the manuscript; JM is the corresponding author.

At RGP, JW, HM and TM contributed to physical map construction, Fiber-FISH analysis, and physical map extension, NM carried out data analysis and TS directed the research.

At UW, WJ and JJ contributed to gap analysis using fiber-FISH.

## Supplementary Material

Additional file 1 Additional tables (5–12) and figures (10–13) are presented in the Supporting On-line Data, which is in pdf. Each table and figure is annotated with a legend. Click here for file
