# Supplementary material for: The sequence of rice chromosomes 11 and 12, rich in disease resistance genes and recent gene duplications
Source: BMC Biol. 2005 Sep 27;3:20. doi: 10.1186/1741-7007-3-20 (PMC1261165; doi:10.1186/1741-7007-3-20)
Supplement: Additional file 1 — Additional tables (5–12) and figures (10–13) are presented in the Supporting On-line Data, which is in pdf. Each table and figure is annotated with a legend. [file 1741-7007-3-20-S1.pdf]

Supporting On-line Data

Table 5. Repetitive sequences on rice chromosome 11. The rice chromosome 11 pseudomolecule was searched against the TIGR *Oryza* Repeat database using RepeatMasker with a cutoff score of 225. Each sub-class was collapsed and quantitated.

Classification	Total length (kp)	Percent in repeat fraction	Percent in dataset	Occurrence	Occurrence (per Mb)	Average size (bp)
**Transposable elements**	7,734.29	92.33%	27.26%	16,407	578.33	471.40
**Retrotransposons**						
Ty1-copia	453.63	5.42%	1.60%	388	13.68	1,169.16
Ty3-gypsy	2,045.76	24.42%	7.21%	1,235	43.53	1,656.49
LINE	9.60	0.11%	0.03%	22	0.78	436.23
SINE	31.24	0.37%	0.11%	221	7.79	141.36
Unclassified Retrotransposons	1,243.77	14.85%	4.38%	1,339	47.20	928.88
**Subtotal (Retrotransposons)**	3,784.01	45.17%	13.34%	3,205	112.97	1,180.66
**Transposons**						
Ac/Ds	24.21	0.29%	0.09%	103	3.63	235.07
CACTA, En/Spm	1,288.32	15.38%	4.54%	1,008	35.53	1,278.09
Mutator (MULE)	59.36	0.71%	0.21%	275	9.69	215.85
Mariner (MLE)	10.67	0.13%	0.04%	31	1.09	344.19
ping/pong/SNOOPY	12.32	0.15%	0.04%	12	0.42	1,027.00
Unclassified Transposons	998.98	11.93%	3.52%	3,470	122.31	287.89
**Subtotal (Transposons)**	2,393.86	28.58%	8.44%	4,899	172.69	488.64
**MITE**						
Tourist	231.25	2.76%	0.82%	891	31.41	259.54
Stowaway	2.63	0.03%	0.01%	31	1.09	84.81
Crackle	23.84	0.28%	0.08%	117	4.12	203.73
Explorer	19.50	0.23%	0.07%	156	5.50	125.01
Gaijin/Gaigin	56.93	0.68%	0.20%	413	14.56	137.84
Castaway	59.53	0.71%	0.21%	235	8.28	253.31
Snap	11.25	0.13%	0.04%	67	2.36	167.93
Amy/LTP	20.30	0.24%	0.07%	70	2.47	289.97
Ditto	78.41	0.94%	0.28%	461	16.25	170.10
Wanderer	44.85	0.54%	0.16%	304	10.72	147.55
p-SINE1	31.50	0.38%	0.11%	199	7.01	158.28
Pop	14.86	0.18%	0.05%	149	5.25	99.70
Krispie	1.49	0.02%	0.01%	13	0.46	114.23
Snabo	23.67	0.28%	0.08%	129	4.55	183.48
MITE-adh, type A	69.83	0.83%	0.25%	360	12.69	193.97
MITE-adh, type B	248.06	2.96%	0.87%	1,177	41.49	210.76
MITE-adh, type D	90.99	1.09%	0.32%	738	26.01	123.29
MITE-adh, type G	21.61	0.26%	0.08%	86	3.03	251.33
MITE-adh, type H	11.90	0.14%	0.04%	49	1.73	242.90
MITE-adh, type I	82.04	0.98%	0.29%	476	16.78	172.36
MITE-adh, type J	29.29	0.35%	0.10%	262	9.24	111.78
MITE-adh, type K	34.27	0.41%	0.12%	220	7.75	155.76

MITE-adh, type L	17.77	0.21%	0.06%	91	3.21	195.25
MITE-adh, type M	25.05	0.30%	0.09%	128	4.51	195.69
MITE-adh, type N	4.21	0.05%	0.01%	32	1.13	131.41
MITE-adh-1	1.11	0.01%	0.00%	7	0.25	158.71
MITE-adh-2	6.05	0.07%	0.02%	30	1.06	201.63
MITE-adh-3	3.18	0.04%	0.01%	17	0.60	187.18
MITE-adh-4	7.82	0.09%	0.03%	34	1.20	230.03
MITE-adh-5	52.67	0.63%	0.19%	221	7.79	238.33
MITE-adh-6	1.04	0.01%	0.00%	6	0.21	174.00
MITE-adh-7	0.19	0.00%	0.00%	1	0.04	190.00
MITE-adh-8	4.60	0.05%	0.02%	35	1.23	131.40
MITE-adh-9	8.44	0.10%	0.03%	52	1.83	162.37
MITE-adh-10	8.31	0.10%	0.03%	56	1.97	148.36
MITE-adh-11	32.16	0.38%	0.11%	79	2.78	407.11
MITE-adh-12	0.88	0.01%	0.00%	6	0.21	146.83
Buhui	8.96	0.11%	0.03%	38	1.34	235.92
Casin	0.87	0.01%	0.00%	9	0.32	97.00
Centre	2.52	0.03%	0.01%	14	0.49	179.71
Delay	2.36	0.03%	0.01%	14	0.49	168.29
ECR	9.44	0.11%	0.03%	46	1.62	205.22
Helia	0.89	0.01%	0.00%	7	0.25	127.00
ID-2	1.04	0.01%	0.00%	3	0.11	346.00
ID-3	7.10	0.08%	0.03%	41	1.45	173.17
ID-4	4.02	0.05%	0.01%	20	0.70	201.10
Lier	0.15	0.00%	0.00%	2	0.07	77.50
Stola	5.37	0.06%	0.02%	19	0.67	282.63
Stone	0.13	0.00%	0.00%	1	0.04	130.00
Susu	8.92	0.11%	0.03%	41	1.45	217.63
Wuji	0.76	0.01%	0.00%	3	0.11	254.33
Youren	12.08	0.14%	0.04%	52	1.83	232.38
Micron	51.66	0.62%	0.18%	272	9.59	189.92
Pangrangja	47.34	0.57%	0.17%	258	9.09	183.48
Kiddo	4.63	0.06%	0.02%	26	0.92	177.92
mPing/miniSNOOPY	0.86	0.01%	0.00%	2	0.07	430.00
MDM	5.85	0.07%	0.02%	37	1.30	158.11
**Subtotal (MITEs)**	1,556.42	18.58%	5.49%	8,303	292.67	187.45
**Centromere-related**						
Centromere-specific Retrotransposon	118.33	1.41%	0.42%	306	10.79	386.69
CentO	17.55	0.21%	0.06%	90	3.17	195.02
Unclassified Centromere Sequences	17.17	0.20%	0.06%	48	1.69	357.71
**Subtotal (Centromere-related)**	153.05	1.83%	0.54%	444	15.65	344.70
**Telomere-related**						
Telomere-associated	7.15	0.09%	0.03%	34	1.20	210.29
Telomere	97.27	1.16%	0.34%	221	7.79	440.14
**Subtotal (Telomere-related)**	104.42	1.25%	0.37%	255	8.99	409.49
**Ribosomal RNA genes**						
45S rDNA	1.94	0.02%	0.01%	18	0.63	107.94
5S rDNA	0.28	0.00%	0.00%	4	0.14	68.75
**Subtotal (Ribosomal RNA genes)**	2.22	0.03%	0.01%	22	0.78	100.82
**Unclassified**	383.07	4.57%	1.35%	1,676	59.08	228.56

**Total**	8,377.05	100.00%	29.53%	18,804	662.83

Table 6. Repetitive sequences on rice chromosome 12. The rice chromosome 12 pseudomolecule was searched against the TIGR *Oryza* Repeat database using RepeatMasker and a cutoff score of 225. Each sub-class was collapsed and quantitated.

Classification	Total length (kp)	Percent in repeat fraction	Percent in dataset	Occurrence	Occurrence (per Mb)	Average size (bp)
**Transposable elements**	7,989.07	92.05%	29.06%	14,979	544.84	533.35
**Retrotransposons**						
Ty1-copia	480.05	5.53%	1.75%	402	14.62	1,194.16
Ty3-gypsy	2,216.05	25.53%	8.06%	1,100	40.01	2,014.59
LINE	18.05	0.21%	0.07%	43	1.56	419.74
SINE	24.03	0.28%	0.09%	196	7.13	122.61
Unclassified Retrotransposons	1,646.31	18.97%	5.99%	1,554	56.52	1,059.40
**Subtotal (Retrotransposons)**	4,384.49	50.52%	15.95%	3,295	119.85	1,330.65
**Transposons**						
Ac/Ds	23.91	0.28%	0.09%	105	3.82	227.75
CACTA, En/Spm	1,324.64	15.26%	4.82%	966	35.14	1,371.26
Mutator (MULE)	56.26	0.65%	0.20%	249	9.06	225.96
Mariner (MLE)	7.66	0.09%	0.03%	25	0.91	306.20
ping/pong/SNOOPY	4.70	0.05%	0.02%	8	0.29	587.12
Unclassified Transposons	784.35	9.04%	2.85%	2,894	105.26	271.03
**Subtotal (Transposons)**	2,201.52	25.37%	8.01%	4,247	154.48	518.37
**MITE**						
Tourist	194.65	2.24%	0.71%	726	26.41	268.12
Stowaway	1.81	0.02%	0.01%	22	0.80	82.50
Crackle	19.44	0.22%	0.07%	91	3.31	213.65
Explorer	19.83	0.23%	0.07%	157	5.71	126.28
Gaijin/Gaigin	55.97	0.64%	0.20%	378	13.75	148.07
Castaway	51.61	0.59%	0.19%	196	7.13	263.32
Snap	8.75	0.10%	0.03%	59	2.15	148.36
Amy/LTP	14.29	0.16%	0.05%	50	1.82	285.70
Ditto	64.20	0.74%	0.23%	403	14.66	159.31
Wanderer	42.70	0.49%	0.16%	276	10.04	154.72
p-SINE1	24.60	0.28%	0.09%	169	6.15	145.58
Pop	12.40	0.14%	0.05%	119	4.33	104.21
Krispie	1.74	0.02%	0.01%	12	0.44	145.17
Snabo	24.10	0.28%	0.09%	125	4.55	192.77
MITE-adh, type A	51.39	0.59%	0.19%	266	9.68	193.20
MITE-adh, type B	229.69	2.65%	0.84%	1,097	39.90	209.38
MITE-adh, type D	87.22	1.00%	0.32%	687	24.99	126.96
MITE-adh, type G	24.01	0.28%	0.09%	101	3.67	237.73
MITE-adh, type H	13.55	0.16%	0.05%	55	2.00	246.31
MITE-adh, type I	70.97	0.82%	0.26%	405	14.73	175.23
MITE-adh, type J	29.84	0.34%	0.11%	271	9.86	110.11
MITE-adh, type K	33.82	0.39%	0.12%	210	7.64	161.05
MITE-adh, type L	12.82	0.15%	0.05%	66	2.40	194.23
MITE-adh, type M	27.04	0.31%	0.10%	151	5.49	179.07
MITE-adh, type N	3.25	0.04%	0.01%	24	0.87	135.50
MITE-adh-1	1.32	0.02%	0.00%	12	0.44	110.42

MITE-adh-2	5.84	0.07%	0.02%	30	1.09	194.60
MITE-adh-3	5.38	0.06%	0.02%	28	1.02	192.18
MITE-adh-4	12.28	0.14%	0.04%	53	1.93	231.60
MITE-adh-5	38.30	0.44%	0.14%	160	5.82	239.37
MITE-adh-6	0.51	0.01%	0.00%	3	0.11	168.67
MITE-adh-8	4.43	0.05%	0.02%	31	1.13	142.87
MITE-adh-9	8.52	0.10%	0.03%	51	1.86	167.06
MITE-adh-10	4.75	0.05%	0.02%	33	1.20	144.09
MITE-adh-11	30.33	0.35%	0.11%	75	2.73	404.36
MITE-adh-12	0.31	0.00%	0.00%	3	0.11	102.67
Buhui	9.08	0.10%	0.03%	30	1.09	302.63
Casin	0.52	0.01%	0.00%	3	0.11	173.67
Centre	1.58	0.02%	0.01%	11	0.40	143.36
Delay	2.27	0.03%	0.01%	12	0.44	189.58
ECR	11.49	0.13%	0.04%	59	2.15	194.76
Helia	1.77	0.02%	0.01%	11	0.40	160.73
ID-2	0.28	0.00%	0.00%	4	0.15	70.75
ID-3	8.65	0.10%	0.03%	46	1.67	188.11
ID-4	3.81	0.04%	0.01%	19	0.69	200.79
Lier	0.48	0.01%	0.00%	4	0.15	119.50
Stola	5.27	0.06%	0.02%	17	0.62	309.88
Stone	0.13	0.00%	0.00%	1	0.04	129.00
Susu	5.82	0.07%	0.02%	26	0.95	223.65
Wuji	0.77	0.01%	0.00%	3	0.11	257.00
Youren	11.33	0.13%	0.04%	48	1.75	235.96
Micron	61.16	0.70%	0.22%	279	10.15	219.20
Pangrangja	33.73	0.39%	0.12%	198	7.20	170.38
Kiddo	4.01	0.05%	0.01%	26	0.95	154.35
mPing/miniSNOOPY	2.15	0.02%	0.01%	5	0.18	430.00
MDM	7.09	0.08%	0.03%	40	1.45	177.22
**Subtotal (MITEs)**	1,403.06	16.17%	5.10%	7,437	270.51	188.66
**Centromere-related**						
Centromere-specific Retrotransposon	73.48	0.85%	0.27%	193	7.02	380.72
CentO	126.85	1.46%	0.46%	79	2.87	1,605.66
Unclassified Centromere Sequences	10.51	0.12%	0.04%	39	1.42	269.36
**Subtotal (Centromere-related)**	210.83	2.43%	0.77%	311	11.31	677.91
**Telomere-related**						
Telomere-associated	9.64	0.11%	0.04%	36	1.31	267.81
Telomere	98.50	1.13%	0.36%	231	8.40	426.42
Subtotal (Telomere-related)	108.14	1.25%	0.39%	267	9.71	405.04
Ribosomal RNA genes						
45S rDNA	8.36	0.10%	0.03%	43	1.56	194.30
5S rDNA	0.36	0.00%	0.00%	5	0.18	72.60
**Subtotal (Ribosomal RNA genes)**	8.72	0.10%	0.03%	48	1.75	181.62
**Unclassified**	362.26	4.17%	1.32%	1,508	54.85	240.22
**Total**	8,679.02	100.00%	31.57%	17,113	622.46	

Table 7. Statistics of chromosome 11 and 12 rice genes with alignments to maize and sorghum genomic assemblies.

Feature of the chromosome	No. rice genes	No. maize assemblies	No. sorghum assemblies	Both
Non-TE	6,451	1,879	2,135	1,469
TE	2,340	160	481	101
Intergenic		366	1,306	N.A.
Total	8,791	2,405	3,922	1,570

Table 8. Summary statistics of the different categories of R-like and defense response genes on rice chromosomes 11 and 12

Region	NBS-LRR	LZ-NBS-LRR	LRR-TM	Misc. Resistance	Defense response	Total
**Chr11**						
Short arm	22	8	18	20	1	69
Centromeric	1	0	0	0	0	1
Long arm	50	13	20	49	16	148
Total	73	21	38	69	17	218
%	9.63	17.43	31.65	7.80	7.80	100.00
**Chr12**						
Short arm	8	3	7	31	4	53
Centromeric	0	0	0	1	0	1
Long arm	10	4	6	18	7	45
Total	18	7	13	50	11	99
%	18.18	7.07	13.13	50.51	11.11	100.00

Table 9. Number of duplicate gene models of chromosome 11 and 12 at different percent coverage.

	**Chromosome 11**			**Chromosome 12**		
**% coverage (≥)**	**First 3 Mb**	**Rest**	**Total**	**First 3 Mb**	**Rest**	**Total**
30	287	163	450	304	189	493
40	270	137	407	280	143	423
50	236	114	350	250	102	352
60	197	85	282	209	77	286
70	160	71	231	167	66	233
80	141	49	190	146	43	189
90	109	31	140	113	31	144

Table 10. List of unique non-TE gene models showing expression only in one chromosome.

Chromosome 11				Chromosome 12			
**Gene model/Locus**	**Predicted function**	**KOME cDNA**	**NCBI EST**	**Gene model/Locus**	**Predicted function**	**KOME cDNA**	**NCBI EST**
m00042|LOC_Os11g01420	ribosomal protein L10, putative	1	1	m00037|LOC_Os12g01360	expressed protein	1	1
m00044|LOC_Os11g01440	expressed protein		9	m00047|LOC_Os12g01460	expressed protein	2	
m00052|LOC_Os11g01510	ubiquitin activating enzyme e1 2	1	8	m00055|LOC_Os12g01530	Ferritin-like domain, putative	1	2
m00061|LOC_Os11g01590	hypothetical protein	1		m00056|LOC_Os12g01530	Ferritin-like domain, putative	1	
m00063|LOC_Os11g01610	PRA1 family protein, putative		1	m00079|LOC_Os12g01730	Multicopper oxidase, putative	1	
m00081|LOC_Os11g01790	Similar to F21B7.20	1		m00083|LOC_Os12g01760	F-box family protein AtFBL3, putative	1	5
m00084|LOC_Os11g01820	probable Na/H antiporter [imported] - Arabidopsis thaliana, putative	1		m00091|LOC_Os12g01830	Staphylococcal nuclease homologue, putative	1	
m00112|LOC_Os11g02100	Peroxidase	1		m00092|LOC_Os12g01850	expressed protein	1	
m00113|LOC_Os11g02110	Similar to gcn5-like protein 1 (rt14 protein homolog)	1		m00100|LOC_Os12g01930	expressed protein	1	2
m00134|LOC_Os11g02300	Similar to MAP kinase - Arabidopsis thaliana	2	4	m00101|LOC_Os12g01930	expressed protein	1	2
m00137|LOC_Os11g02320	expressed protein	4	3	m00112|LOC_Os12g02040	Hypoxia induced protein conserved region, putative	2	
m00139|LOC_Os11g02330	expressed protein	1		m00137|LOC_Os12g02280	expressed protein	1	
m00146|LOC_Os11g02390	Protease inhibitor/seed storage/LTP family, putative	1		m00138|LOC_Os12g02290	nonspecific lipid-transfer protein 5 precursor (ltp 5).	3	
m00149|LOC_Os11g02420	lipid-transfer protein - maize	3		m00141|LOC_Os12g02320	Protease inhibitor/seedstorage/LTP family, putative	1	
m00165|LOC_Os11g02580	alpha-hydroxy nitrile lyase, putative	1		m00147|LOC_Os12g02370	Chalcone-flavanone isomerase	1	
m00173|LOC_Os11g02660	alpha/beta hydrolase, putative	1	2	m00181|LOC_Os12g02690	expressed protein	1	1
m00174|LOC_Os11g02660	alpha/beta hydrolase, putative	1	2	m00182|LOC_Os12g02690	expressed protein	1	1
m00230|LOC_Os11g03200	expressed protein	1		m00183|LOC_Os12g02690	expressed protein	1	1
m00234|LOC_Os11g03240	MATE efflux family protein, putative	1	2	m00184|LOC_Os12g02690	expressed protein	1	1
m00252|LOC_Os11g03420	ZF-HD protein dimerisation region, putative	1		m00187|LOC_Os12g02720	expressed protein	1	
m00259|LOC_Os11g03490	CDS		1	m00191|LOC_Os12g02760	expressed protein	1	
m00260|LOC_Os11g03500	MATE efflux family protein, putative		1	m00195|LOC_Os12g02800	expressed protein	1	
m00268|LOC_Os11g03580	expressed protein	1		m00196|LOC_Os12g02800	expressed protein	1	
m00269|LOC_Os11g03590	Nse1 non-SMC component of SMC5-6 complex, putative	2		m00201|LOC_Os12g02840	probable Na/H antiporter [imported] - Arabidopsis thaliana	1	
m00272|LOC_Os11g03620	expressed protein	1		m00215|LOC_Os12g02980	nod factor binding lectin-nucleotide phosphohydrolase	1	6
m00288|LOC_Os11g03780	arabinoxylan arabinofuranohydrolase isoenzyme AXAH-II	1	3	m00216|LOC_Os12g02980	nod factor binding lectin-nucleotide phosphohydrolase	1	6
m00289|LOC_Os11g03780	arabinoxylan arabinofuranohydrolase isoenzyme AXAH-II	1	3	m00226|LOC_Os12g03070	FHA domain, putative	1	7
m00290|LOC_Os11g03790	expressed protein		1	m00227|LOC_Os12g03070	FHA domain, putative	2	8
m00300|LOC_Os11g03890	expressed protein	1		m00228|LOC_Os12g03070	FHA domain, putative	3	5
m00313|LOC_Os11g04020	Major Facilitator Superfamily	1	1	m00231|LOC_Os12g03100	Exostosin family, putative	1	
m00314|LOC_Os11g04030	Major Facilitator Superfamily	2	1	m00236|LOC_Os12g03150	myb protein homolog – rice	1	
m00315|LOC_Os11g04030	Major Facilitator Superfamily	2	2	m00241|LOC_Os12g03200	CDS		1
m00332|LOC_Os11g04180	Similar to dual specificity protein phosphatase 12	1		m00247|LOC_Os12g03260	MATE efflux family protein, putative	1	1

m00334|LOC_Os11g04190	Similar to probable auxin transport protein - rice	1	1	m00250|LOC_Os12g03270	expressed protein	1	1
m00344|LOC_Os11g04290	Cytochrome P450	1		m00259|LOC_Os12g03370	expressed protein	1	
m00350|LOC_Os11g04350	expressed protein	2		m00262|LOC_Os12g03400	expressed protein	1	
m00353|LOC_Os11g04380	expressed protein	1		m00266|LOC_Os12g03440	F-box domain, putative	1	
m00367|LOC_Os11g04520	TAP42-like family	1	5	m00270|LOC_Os12g03470	arabinoxylan arabinofuranohydrolase isoenzyme AXAH-II	1	7
m00389|LOC_Os11g04720	response regulator 6	1		m00271|LOC_Os12g03470	arabinoxylan arabinofuranohydrolase isoenzyme AXAH-II	1	7
m00400|LOC_Os11g04830	Similar to embryogenesis transmembrane protein - maize	1		m00306|LOC_Os12g03810	probable serine/threonine kinase (EC 2.7.1.-) SNFL1 – sorghum		1
m00486|LOC_Os11g05660	F-box domain, putative	1		m00321|LOC_Os12g03960	similar to RIKEN cDNA 2810407B07 gene	1	
m00489|LOC_Os11g05690	amino acid transporter-like protein		1	m00327|LOC_Os12g04020	hydroxymethylglutaryl-CoA lyase	1	
m00641|LOC_Os11g07040	Protein kinase domain, putative	1	2	m00353|LOC_Os12g04270	biogenesis protein	1	2
m02139|LOC_Os11g24070	Protease inhibitor/seed storage/LTP family, putative	2		m00369|LOC_Os12g04430	hypothetical protein		1
m02240|LOC_Os11g25080	hypothetical protein	2		m00385|LOC_Os12g04590	Similar to ring finger protein		1
m02899|LOC_Os11g31590	Armadillo/beta-catenin-like repeat, putative		1	m00386|LOC_Os12g04590	Similar to ring finger protein		1
m03303|LOC_Os11g35970	expressed protein		1	m00393|LOC_Os12g04660	Similar to ring finger protein	1	
m04344|LOC_Os11g45890	expressed protein	3		m00415|LOC_Os12g04860	DTW domain, putative	1	
m04420|LOC_Os11g47120	hypothetical protein		2	m00416|LOC_Os12g04860	DTW domain, putative	1	
m04433|LOC_Os11g47250	hypothetical protein		1	m00430|LOC_Os12g04990	long-chain acyl-CoA synthetase		5
m04442|LOC_Os11g47330	expressed protein		2	m00431|LOC_Os12g04990	long-chain acyl-CoA synthetase	1	5
				m00433|LOC_Os12g05000	At1g03250/F15K9_13		1
				m00445|LOC_Os12g05110	Pyruvate kinase, barrel domain	1	6
				m00455|LOC_Os12g05210	AT3g10020/T22K18_16	1	
				m00474|LOC_Os12g05400	Similar to protein kinase-like protein - Arabidopsis thaliana	1	
				m00639|LOC_Os12g06930	hypothetical protein	1	
				m00662|LOC_Os12g07140	V-type ATPase, C subunit	3	
				m00768|LOC_Os12g08130	Transmembrane amino acid transporter protein	6	9
				m03252|LOC_Os12g33300	Integral membrane protein DUF6, putative	1	
				m03811|LOC_Os12g38640	At5g08050/F13G24_250	2	3

Table 11. List of chromosome 11 rice genes showing significant homology with wheat contigs containing bin-mapped ESTs

S. No.	Rice gene information			BLAST search results			Mapped wEST	
	Gene model/ locus number	Predicted cDNA product	Predicted cDNA length	Score Bit	Expect	Identities	contig no	Bin
1	m00017|Os11g01170	cDNA expressed protein	1255	830	0	648/763 (84%)	12025	C-5AL10-0.57*
								C-5BL14-0.75*
								C-5DL1-0.60
2	m00019|Os11g01190	cDNA RNA recognition motif.	4095	548	e-155	451/552 (81%)	10101	5BL
3	m00033|Os11g01330	cDNA expressed protein	3702	665	0	618/797 (77%)	8417	5AL12-0.35-0.57
								5BL1-0.55-0.75
								C-5DL1-0.60
4	m00036|Os11g01360	cDNA hypothetical protein	231	236	5E-63	189/230 (82%)	12468	1AS1-0.47-0.86
								1BS9-0.84-1.06
5	m00037|Os11g01370	cDNA expressed protein	1578	240	2E-63	340/524 (64%)	17623	1AL1-0.17-0.61
								1BL1-0.47-0.69
								1DL
6	m00038|Os11g01380	cDNA clathrin heavy chain,	5573	2044	0	1679/1989 (84%)	16778	4BS4-0.37-0.57
7	m00042|Os11g01420	cDNA ribosomal protein L10, putative	1107	819	0	618/715 (86%)	18290	1AL3-0.61-1.00
								C-5AL10-0.57*
								6DS2-0.45-0.79
8	m00057|Os11g01550	cDNA Similar to lob domain protein 12	836	202	2E-52	216/305 (70%)	14992	C-1BS10-0.50
								C-1DS3-0.48
								4BS4-0.37-0.57
								C-4DS1-0.53
9	m00062|Os11g01600	cDNA MATE efflux family protein, putative	2632	469	e-132	379/448 (84%)	18382	C-5AL10-0.57*
								C-5BL14-0.75*
								C-5DL1-0.60
10	m00063|Os11g01610	cDNA PRA1 family protein, putative	987	185	5E-47	276/432 (63%)	10949	5BL9-0.76-0.79
								5DL5-0.76-1.00

11	m00064|Os11g01620	cDNA Leucine Rich Repeat, putative	1938	110	3E-24	138/207 (66%)	18170	3AL3-0.42-0.78
								3BL2-0.22-0.50
12	m00075|Os11g01730	cDNA Multicopper oxidase, putative	1704	529	e-150	442/541 (81%)	8434	3BL7-0.63-1.00
13	m00076|Os11g01740	cDNA Protein kinase domain, putative	2067	361	1E-99	340/448 (75%)	11579	3BS1-0.33-0.57
14	m00080|Os11g01780	cDNA F-box family protein AtFBL3, putative	2406	811	0	677/838 (80%)	8733	C-5BL14-0.75*
15	m00112|Os11g02100	cDNA Peroxidase	1144	264	8E-71	427/678 (62%)	13267	4AS1-0.20-0.63
								C-4BL1-0.71
								4DL9-0.31-0.56
16	m00115|Os11g02130	cDNA peroxidase	975	250	1E-66	371/585 (63%)	13267	4AS1-0.20-0.63
								C-4BL1-0.71
								4DL9-0.31-0.56
17	m00117|Os11g02150	cDNA expressed protein	1108	225	6E-59	211/276 (76%)	15698	5AL12-0.35-0.57
								5BL6-0.29-0.55
								C-5BL6-0.29
								C-5DL1-0.60
18	m00118|Os11g02150	cDNA expressed protein	1231	489	e-138	466/619 (75%)	17031	C-4AL12-0.43
								C-4BS4-0.37
								4BS4-0.37-0.57
								4DS1-0.53-0.67
19	m00119|Os11g02150	cDNA expressed protein	1286	489	e-138	466/619 (75%)	17031	C-4AL12-0.43
								C-4BS4-0.37
								4BS4-0.37-0.57
								4DS1-0.53-0.67
20	m00124|Os11g02200	cDNA expressed protein	936	134	8E-32	98/112 (87%)	11885	4AL
21	m00128|Os11g02240	cDNA protein kinase homolog - rice	2044	1483	0	1315/1665 (78%)	17610	C-5AL12-0.35
								5BL6-0.29-0.55
								C-5DL1-0.60
								7BS1-0.27-1.00
22	m00139|Os11g02330	cDNA expressed protein	750	274	7E-74	237/297 (79%)	18722	4AL12-0.43-0.59
								4BS4-0.37-0.57
								4DS1-0.53-0.67
23	m00140|Os11g02330	cDNA expressed protein	723	274	7E-74	237/297 (79%)	18722	4AL12-0.43-0.59
								4BS4-0.37-0.57

								4DS1-0.53-0.67
24	m00142|Os11g02350	cDNA lipid transfer protein	741	168	6E-42	233/356 (65%)	18810	2BS1-0.53-0.75
								2DS1-0.33-0.47
25	m00144|Os11g02370	cDNA nonspecific lipid-transferprotein 2 precursor (ltp 2)	919	196	2E-50	239/335 (71%)	17231	3BS8-0.78-1.00
26	m00146|Os11g02390	cDNA Protease inhibitor/seed storage/LTP family, putative	808	263	2E-70	261/342 (76%)	17231	3BS8-0.78-1.00
27	m00147|Os11g02400	cDNA lipid-transfer protein - Maize	965	244	1E-64	257/351 (73%)	18810	2BS1-0.53-0.75
								2DS1-0.33-0.47
28	m00149|Os11g02420	cDNA lipid-transfer protein -Maize	915	261	6E-70	293/397 (73%)	18810	2BS1-0.53-0.75
								2DS1-0.33-0.47
29	m00152|Os11g02450	cDNA Elongation factor P (EF-P), putative	690	714	0	554/653 (84%)	9961	C-4BS4-0.37
								4DS1-0.53-0.67
30	m00153|Os11g02460	cDNA outer membrane protein, OMP85 family, putative	4186	900	0	878/1179 (74%)	15236	4AS4-0.63-0.76
								4DL9-0.31-0.56
								C-4BL1-0.71
31	m00171|Os11g02640	cDNA Similar to expressed protein-like protein	1545	478	e-135	488/660 (73%)	1723	4AL12-0.43-0.59
								4BS4-0.37-0.57
								4DS1-0.53-0.67
32	m00192|Os11g02830	cDNA protein kinase, putative	1550	480	e-135	348/394 (88%)	4367	C-5AL10-0.57*
								C-5BL14-0.75*
								C-5DL1-0.60
33	m00193|Os11g02840	cDNA Similar to protein kinase-like protein	636	372	e-103	296/348 (85%)	4367	C-5AL10-0.57*
								C-5BL14-0.75*
								C-5DL1-0.60
34	m00195|Os11g02860	cDNA protein kinase, putative	483	261	3E-70	197/225 (87%)	4367	C-5AL10-0.57*
								C-5BL14-0.75*
								C-5DL1-0.60
35	m00214|Os11g03050	cDNA Similar to phosphoethanolamine cytidylyl transferase	2153	228	1E-59	243/343 (70%)	11242	2AL
36	m00233|Os11g03230	cDNA apyrase, putative	1290	588	e-168	612/849 (72%)	17912	4BL5-0.86-1.00
								4DL13-0.56-

								1.00*
								5AL23-0.87-1.00
37	m00237|Os11g03270	cDNA apyrase GS52	1647	805	0	797/1082 (73%)	17912	4BL5-0.86-1.00
								4DL13-0.56-1.00*
								5AL23-0.87-1.00
38	m00239|Os11g03290	cDNA S-type apyrase	1701	819	0	886/1221 (72%)	17912	4BL5-0.86-1.00
								4DL13-0.56-1.00*
								5AL23-0.87-1.00
39	m00247|Os11g03370	cDNA No apical meristem (NAM) protein, putative	1569	109	6E-24	122/176 (69%)	4891	2AS5-0.78-1.00
								2DS5-0.47-1.00
								4AL12-0.43-0.59
								4BS1-0.81-1.00
								4DS2-0.82-1.00
								7BL7-0.63-0.78
								7DL2-0.61-0.82
40	m00248|Os11g03380	cDNA expressed protein	1290	717	0	625/788 (79%)	17274	C-5BL14-0.75*
								C5B
								C5D
41	m00254|Os11g03440	cDNA myb protein homolog - rice	1035	261	6E-70	272/380 (71%)	3976	C-6AS5-0.65*
								C-6AL4-0.55
								6BL
								6DL6-0.29-0.68*
								C-6DL6-0.29
42	m00260|Os11g03500	cDNA MATE efflux family protein, putative	1488	150	2E-36	215/336 (63%)	11753	3AL5-0.78-1.00
								3BL7-0.63-1.00
								3DL3-0.81-1.00
43	m00264|Os11g03540	cDNA AP2 domain, putative	1642	163	4E-40	248/389 (63%)	17427	3AL3-0.42-0.78
								3BL10-0.50-0.63
								3DL2-0.27-0.81
44	m00267|Os11g03570	cDNA hypothetical protein	1848	516	e-146	564/768 (73%)	8419	C-4AL12-0.43

								4BS4-0.37-0.57
								4DS1-0.53-0.67
								C-5AL10-0.57*
								C-5BL14-0.75*
45	m00281|Os11g03710	cDNA small zinc finger-like protein	541	239	2E-63	226/297 (76%)	8560	C-4AL12-0.43
								C-4BS4-0.37
								4DS3-0.67-0.82
46	m00283|Os11g03730	cDNA Similar to arabinoxylan arabinofurano hydrolase isoenzyme AXAH-II	3843	513	e-145	448/558 (80%)	14869	C-5AL10-0.57*
								C-5BL14-0.75*
47	m00288|Os11g03780	cDNA arabinoxylan arabinofurano hydrolase isoenzyme AXAH-II	2420	668	0	614/797 (77%)	14869	C-5AL10-0.57*
								C-5BL14-0.75*
48	m00289|Os11g03780	cDNA arabinoxylan arabinofurano hydrolase isoenzyme AXAH-II	2416	668	0	614/797 (77%)	14869	C-5AL10-0.57*
								C-5BL14-0.75*
49	m00308|Os11g03970	cDNA probable serine/threonine kinase (EC 2.7.1.-) SNFL1 - sorghum	1907	627	e-180	742/1079 (68%)	17053	4BL5-0.86-1.00
								4DL12-0.71-1.00
								5AL23-0.87-1.00
50	m00309|Os11g03980	cDNA Similar to calmodulin	501	293	8E-80	268/352 (76%)	16205	1AL3-0.61-1.00
51	m00310|Os11g03990	cDNA probable WD-40 repeat	1818	1283	0	1031/1223 (84%)	16831	C-4AL12-0.43
								C-4BS4-0.37
								4DS1-0.53-0.67
52	m00311|Os11g04000	cDNA expressed protein	1232	134	1E-31	192/290 (66%)	12110	1AS3-0.86-1.00
								C1D
53	m00312|Os11g04010	cDNA Similar to latex-abundant protein	1311	259	2E-69	312/456 (68%)	18138	3DL2-0.27-0.81
								3BL2-0.22-0.50
54	m00321|Os11g04070	cDNA ribosomal protein L10, putative	1120	1144	0	846/961 (88%)	18665	C7A
								C4A
55	m00331|Os11g04170	cDNA calcium-dependent calmodulin-independent protein kinase	1935	706	0	892/1334 (66%)	18270	C-2AS5-0.78
								C-2BS1-0.53
								C-2DS1-0.33

56	m00344|Os11g04290	cDNA Cytochrome P450	1927	182	8E-46	222/330 (67%)	13305	3BS9-0.57-0.78
								3DS6-0.55-1.00
								3AS4-0.45-1.00
57	m00346|Os11g04310	cDNA Cytochrome P450	1548	196	3E-50	219/315 (69%)	13305	3BS9-0.57-0.78
								3DS6-0.55-1.00
								3AS4-0.45-1.00
58	m00372|Os11g04560	cDNA EF hand, putative	771	196	2E-50	190/242 (78%)	16191	4AL13-0.59-0.66
								3AL5-0.78-1.00
59	m00374|Os11g04580	cDNA hypothetical protein	633	206	2E-53	230/316 (72%)	12559	7DL3-0.82-1.00
								7AL1-0.39-0.71
								6DS2-0.45-0.79
60	m00405|Os11g04880	cDNA Ubiquitin family	624	323	9E-89	220/237 (92%)	18275	C-5AL10-0.57*
								C-5BL14-0.75*
								4DS1-0.53-0.67
61	m00416|Os11g04990	cDNA At1g03250/F15K9_13	1001	442	e-124	371/454 (81%)	15108	C-5DL1-0.60
								5AL12-0.35-0.57
								5BL1-0.55-0.75
62	m00417|Os11g05000	cDNA hypothetical protein	1467	110	2E-24	207/325 (63%)	8273	5DL1-0.60-0.74
								C-5BL9-0.76*
								6BL5-0.40-1.00
								5AL10-0.57-0.78
								5BL14-0.75-0.76
63	m00423|Os11g05050	cDNA aluminium-induced protein, putative	1254	770	0	584/683 (85%)	17620	C-7BS1-0.27
								7DS5-0.36-0.61
64	m00429|Os11g05110	cDNA pyruvate kinase	2066	1957	0	1615/1936 (83%)	18758	3BL7-0.63-1.00
								1BS10-0.50-0.84
65	m00438|Os11g05190	cDNA phytosulfokines 2 precursor	1053	114	2E-25	88/105 (83%)	7579	C-5BL14-0.75*
								C-5AL10-0.57*
66	m00458|Os11g05380	cDNA Cytochrome P450	1871	134	2E-31	206/323 (63%)	13305	3BS9-0.57-0.78
								3DS6-0.55-1.00
								3AS4-0.45-1.00
67	m00460|Os11g05400	cDNA Ser/Thr protein phosphatase family protein, putative	2002	1042	0	754/846 (89%)	8465	C-5BL14-0.75*
								C-5AL10-0.57*

								7AL18-0.90-1.00
68	m00461|Os11g05410	cDNA purple acid phosphatase	1320	637	0	624/832 (75%)	8465	C-5BL14-0.75*
								C-5AL10-0.57*
								7AL18-0.90-1.00
69	m00471|Os11g05510	cDNA hypothetical protein	342	187	5E-48	169/216 (78%)	1034	4AL4-0.80-1.00
70	m00472|Os11g05520	cDNA Eukaryotic-type carbonic anhydrase	549	160	1E-39	150/195 (76%)	1034	4AL4-0.80-1.00
71	m00474|Os11g05540	cDNA rac GTPase activating protein 1	1440	1111	0	788/876 (89%)	15039	5BL1-0.55-0.75
72	m00490|Os11g05700	cDNA ABC transporter, putative	4981	743	0	578/688 (84%)	13087	7DS4-0.61-1.00
								7BS1-0.27-1.00
								7AS8-0.45-0.89*
73	m00493|Os11g05730	cDNA histone H3 - maize	507	553	e-158	373/398 (93%)	14705	7AS5-0.59-0.89
74	m00494|Os11g05740	cDNA Similar to probable AV2-like DNA binding protein [imported] - Arabidopsis thaliana	942	641	0	570/716 (79%)	8636	C-2AL1-0.85
								2DL9-0.76-1.00
								C-5DL1-0.60
								5BL6-0.29-0.55
								C-5AL12-0.35
								2BL4-0.50-0.89
75	m00496|Os11g05760	cDNA At2g24280/F27D4.19	1536	321	7E-88	425/633 (67%)	16864	7AL1-0.39-0.71
76	m00500|Os11g05800	cDNA TB2/DP1, HVA22 family, putative	654	187	1E-47	255/388 (65%)	9554	2DL3-0.49-0.76
								C-2AL1-0.85
								2BL2-0.36-0.50
77	m00515|Os11g05930	cDNA Similar to timing of CAB expression 1-like protein	2351	773	0	656/820 (80%)	9929	4BS4-0.37-0.57
								4AL12-0.43-0.59
								4DS1-0.53-0.67
78	m00539|Os11g06140	cDNA Similar to At1g64630	1760	578	e-165	570/776 (73%)	13056	3BS1-0.33-0.57
								C-6BS5-0.76
								5DL1-0.60-0.74
								C-5BL14-0.75*
								5AL17-0.78-0.87
								C-6AS1-0.35
79	m00562|Os11g06340	cDNA ubiquinol--cytochrome-c reductase, putative	291	229	6E-61	176/208 (84%)	14102	C-5BL14-0.75*

								C-5DL1-0.60
								C-5AL10-0.57*
80	m00567|Os11g06390	cDNA actin	1709	1505	0	1094/1229 (89%)	17177	C-3DL2-0.27
								1DS3-0.48-0.59
								5AL12-0.35-0.57
								C-5BL6-0.29
								C-5DL1-0.60
81	m00568|Os11g06390	cDNA actin	1723	1505	0	1094/1229 (89%)	17177	C-3DL2-0.27
								1DS3-0.48-0.59
								5AL12-0.35-0.57
								C-5BL6-0.29
								C-5DL1-0.60
82	m00569|Os11g06390	cDNA actin	1701	1505	0	1094/1229 (89%)	17177	C-3DL2-0.27
								1DS3-0.48-0.59
								5AL12-0.35-0.57
								C-5BL6-0.29
								C-5DL1-0.60
83	m00571|Os11g06410	cDNA expressed protein	2582	594	e-169	565/736 (76%)	3898	C-5AL10-0.57*
								C-5BL14-0.75*
								C-5DL1-0.60
84	m00572|Os11g06410	cDNA expressed protein	1891	594	e-170	565/736 (76%)	3898	C-5AL10-0.57*
								C-5BL14-0.75*
								C-5DL1-0.60
85	m00573|Os11g06410	cDNA expressed protein	1810	594	e-170	565/736 (76%)	3898	C-5AL10-0.57*
								C-5BL14-0.75*
								C-5DL1-0.60
86	m00585|Os11g06520	cDNA Thiamine pyrophosphate enzyme, N-terminal TPP binding domain, putative	426	275	1E-74	205/237 (86%)	18633	4AS1-0.20-0.63
								2AS5-0.78-1.00
87	m00604|Os11g06700	cDNA hypothetical protein	2079	767	0	662/826 (80%)	697	4AL12-0.43-0.59
								4BS8-0.57-0.81
								4DS1-0.53-0.67

88	m00606|Os11g06720	cDNA abscisic acid- and stress-induced protein - rice	851	396	e-110	340/421 (80%)	17732	4DS1-0.53-0.67
89	m00608|Os11g06740	cDNA hypothetical protein	642	554	e-158	419/489 (85%)	9833	4BS4-0.37-0.57
								4DS1-0.53-0.67
								C-4AL12-0.43
90	m00609|Os11g06750	cDNA ribosomal protein L3, putative	1170	1431	0	1039/1171 (88%)	18673	5BL6-0.29-0.55
91	m00610|Os11g06760	cDNA hypothetical protein	909	467	e-132	348/402 (86%)	13940	4DS1-0.53-0.67
								C-4AL12-0.43
92	m00612|Os11g06780	cDNA serine/threonine protein kinase-like protein	1167	131	9E-31	250/408 (61%)	16028	1AL3-0.61-1.00
								1BL2-0.69-1.00*
								1DL
								4AL12-0.43-0.59
								4DS1-0.53-0.67
								C-6AL4-0.55
93	m00623|Os11g06890	cDNA V-type ATPase, C subunit, putative	498	635	0	449/498 (90%)	17669	4AL12-0.43-0.59
								4BS8-0.57-0.81
								4DS1-0.53-0.67
								C-5AS1-0.40
								C-5BL6-0.29
94	m00636|Os11g07020	cDNA Fructose-bisphosphate aldolase class-I	1928	946	0	676/756 (89%)	18751	4DS1-0.53-0.67
95	m00637|Os11g07020	cDNA Fructose-bisphosphate aldolase class-I	1031	502	e-142	356/396 (89%)	18751	4DS1-0.53-0.67
96	m00638|Os11g07020	cDNA Fructose-bisphosphate aldolase class-I	1752	1456	0	1049/1180 (88%)	18751	4DS1-0.53-0.67
97	m00639|Os11g07020	cDNA Fructose-bisphosphate aldolase class-I	1677	702	0	510/578 (88%)	18751	4DS1-0.53-0.67
98	m00641|Os11g07040	cDNA Protein kinase domain, putative	2582	939	0	963/1324 (72%)	16833	2DL9-0.76-1.00
								C-2AL1-0.85
99	m00682|Os11g07440	cDNA invertase, putative	2320	602	e-172	442/505 (87%)	9906	4DS3-0.67-0.82
								4BS8-0.57-0.81
100	m00690|Os11g07510	cDNA growth regulator protein, putative	1236	312	4E-85	251/306 (82%)	11653	2BL2-0.36-0.50
101	m00724|Os11g07850	cDNA Protein-tyrosine phosphatase	1511	378	e-105	422/591 (71%)	10914	C-2AS5-0.78

								C-2DS1-0.33
								C-2BS1-0.53
102	m00730|Os11g07910	cDNA Endomembrane protein 70	1713	955	0	760/913 (83%)	13728	5B
103	m00731|Os11g07920	cDNA Similar to seven transmembrane protein Mlo4	2943	307	3E-83	308/408 (75%)	2231	2AS5-0.78-1.00
								2BS1-0.53-0.75
								2DS5-0.47-1.00
								4AL12-0.43-0.59
								4BS1-0.81-1.00
								4DS2-0.82-1.00
104	m00735|Os11g07960	cDNA Transferase family	1491	316	2E-86	524/824 (63%)	2911	6AS5-0.65-1.00
								6BS-Sat
105	m00750|Os11g08100	cDNA nucellin-like aspartic protease	1924	385	e-107	304/366 (83%)	12995	4AL12-0.43-0.59
								C-4BS4-0.37
106	m00752|Os11g08120	cDNA Glycosyl hydrolase family 1	1173	353	2E-97	407/577 (70%)	16566	5AL12-0.35-0.57
								5BL1-0.55-0.75
								C-5AL10-0.57*
								C-5DL1-0.60
107	m00759|Os11g08190	cDNA Similar to nucellin-like aspartic protease	924	329	2E-90	285/353 (80%)	12995	4AL12-0.43-0.59
								C-4BS4-0.37
108	m00760|Os11g08200	cDNA nucellin-like protein	1463	128	1E-29	188/286 (65%)	12995	4AL12-0.43-0.59
								C-4BS4-0.37
109	m00771|Os11g08300	cDNA aldehyde dehydrogenase, putative	1668	1185	0	967/1187 (81%)	18389	1AL3-0.61-1.00
								1BL1-0.47-0.69
								1DL2-0.41-1.00
110	m00773|Os11g08320	cDNA Putative methyltransferase	594	309	2E-84	291/388 (75%)	7274	7AL1-0.39-0.71
								7BL2-0.33-0.63
								7DL5-0.30-0.61
111	m00779|Os11g08380	cDNA 1-aminocyclopropane-1-carboxylate oxidase	1473	310	1E-84	399/583 (68%)	16848	C-5AL17-0.78*
								5BL
								6AL8-0.90-1.00
								6BL5-0.40-1.00

								6DL
								6DL1-0.47-0.74*
112	m00782|Os11g08410	cDNA GATA zinc finger, putative	1296	123	3E-28	136/195 (69%)	1937	5AS3-0.75-0.98
								5BS6-0.81-1.00
								5DS2-0.78-1.00
113	m00785|Os11g08440	cDNA dnaK protein, putative	4092	312	1E-84	502/798 (62%)	17156	6BS-Sat
								6AS5-0.65-1.00
114	m00787|Os11g08460	cDNA Similar to dnaK-type molecular chaperone Nthsp70 - common tobacco	1751	310	2E-84	468/731 (64%)	17156	6BS-Sat
								6AS5-0.65-1.00
115	m00788|Os11g08470	cDNA dnaK protein	1707	294	1E-79	502/804 (62%)	17156	6BS-Sat
								6AS5-0.65-1.00
116	m00800|Os11g08580	cDNA Similar to cycloartenol synthase	888	396	e-110	349/449 (77%)	16219	6BS5-0.76-1.05
117	m00836|Os11g08940	cDNA dna-directed rna polymerase ii 8.2 kda polypeptide (ec 2.7.7.6)	539	237	5E-63	171/193 (88%)	15837	3AL3-0.42-0.78
								3DL2-0.27-0.81
								3BL10-0.50-0.63
118	m00844|Os11g09020	cDNA Transmembrane amino acid transporter protein	1787	213	2E-55	185/236 (78%)	7914	C-2AL1-0.85
								C-2DL3-0.49
119	m00856|Os11g09140	cDNA expressed protein	1588	179	6E-45	247/372 (66%)	15023	3AL5-0.78-1.00
								3DL2-0.27-0.81
								3BL7-0.63-1.00
120	m00870|Os11g09280	cDNA protein disulfide isomerase2 precursor	1945	1640	0	1299/1549 (83%)	18681	4AL5-0.66-1.00*
								4DS1-0.53-0.67
								C-4BL1-0.71
121	m00871|Os11g09280	cDNA protein disulfide isomerase2 precursor	2024	1640	0	1299/1549 (83%)	18681	4AL5-0.66-1.00*
								4DS1-0.53-0.67
								C-4BL1-0.71
122	m00875|Os11g09320	cDNA seed protein B32E	1835	524	e-149	658/911 (72%)	14763	4BS8-0.57-0.81
								4DS3-0.67-0.82
123	m00955|Os11g10100	cDNA similar to mitogen-activated protein kinases	2147	890	0	811/1026 (79%)	14734	4AL12-0.43-0.59

								4DS3-0.67-0.82
124	m00973|Os11g10280	cDNA Similar to receptor-like protein kinase	2373	277	2E-74	265/356 (74%)	2160	7AL1-0.39-0.71
								C-7DL5-0.30
125	m00974|Os11g10290	cDNA Similar to receptor-like protein kinase	2412	152	1E-36	136/177 (76%)	2160	7AL1-0.39-0.71
								C-7DL5-0.30
126	m00992|Os11g10460	cDNA peroxidase, putative	1633	104	2E-22	266/452 (58%)	16087	3AS2-0.23-0.45
								C-3BS1-0.33
127	m00994|Os11g10480	cDNA alcohol dehydrogenase I	1627	1025	0	739/832 (88%)	18568	C4A
								C-4BS4-0.37
								4DS3-0.67-0.82
128	m00995|Os11g10480	cDNA alcohol dehydrogenase I	1862	1369	0	1008/1148 (87%)	18568	C4A
								C-4BS4-0.37
								4DS3-0.67-0.82
129	m00996|Os11g10480	cDNA alcohol dehydrogenase I	1884	1443	0	1029/1148 (89%)	18568	C4A
								C-4BS4-0.37
								4DS3-0.67-0.82
130	m00999|Os11g10510	cDNA alcohol dehydrogenase 2	1716	1141	0	926/1133 (81%)	18568	C4A
								C-4BS4-0.37
								4DS3-0.67-0.82
131	m01000|Os11g10520	cDNA alcohol dehydrogenase	1616	752	0	671/863 (77%)	18568	C4A
								C-4BS4-0.37
								4DS3-0.67-0.82
132	m01012|Os11g10640	cDNA Similar to stem rust resistance protein	2109	220	3E-57	391/619 (63%)	12999	C-4AL12-0.43
								4DS3-0.67-0.82
133	m01016|Os11g10680	cDNA expressed protein	978	171	9E-43	281/440 (63%)	3115	C-6BL3-0.36
134	m01017|Os11g10690	cDNA Protein kinase domain, putative	1334	175	5E-44	373/616 (60%)	12999	C-4AL12-0.43
								4DS3-0.67-0.82
135	m01019|Os11g10710	cDNA Similar to barley stem rust resistance protein	1959	191	1E-48	305/475 (64%)	3115	C-6BL3-0.36
136	m01046|Os11g10980	cDNA pyruvate kinase	2373	605	e-173	989/1582	18758	1BS10-0.50-0.84

						(62%)		
								3BL7-0.63-1.00
137	m01050|Os11g11020	cDNA Similar to DAG protein homolog F18F4.120 - Arabidopsis thalian	1500	196	3E-50	225/322 (69%)	17856	2BS3-0.84-1.00
								2DS5-0.47-1.00
								7DS5-0.36-0.61
138	m01152|Os11g12020	cDNA hypothetical protein	270	187	4E-48	189/261 (72%)	10912	4AL12-0.43-0.59
								4DS3-0.67-0.82
139	m01155|Os11g12050	cDNA NB-ARC domain, putative	3373	275	1E-73	245/317 (77%)	10912	4AL12-0.43-0.59
								4DS3-0.67-0.82
140	m01184|Os11g12340	cDNA NB-ARC domain, putative	3054	217	4E-56	277/403 (68%)	10912	4AL12-0.43-0.59
								4DS3-0.67-0.82
141	m01185|Os11g12350	cDNA expressed protein	1137	193	2E-49	216/311 (69%)	10912	4AL12-0.43-0.59
								4DS3-0.67-0.82
142	m01203|Os11g12530	cDNA Leucine Rich Repeat, putative	3351	134	3E-31	185/281 (65%)	2609	5BS4-0.43-0.56
								4AL4-0.80-1.00
143	m01226|Os11g12760	cDNA O-methyltransferase	1137	729	0	828/1147 (72%)	17941	6AL8-0.90-1.00
								6BL5-0.40-1.00
								6DL10-0.80-1.00
144	m01232|Os11g12820	cDNA Similar to sucrose phosphate synthase	603	142	2E-34	130/166 (78%)	8317	5AL23-0.87-1.00
145	m01241|Os11g13400	cDNA hypothetical protein	231	115	1E-26	99/126 (78%)	13425	2BL2-0.36-0.50
								2DL3-0.49-0.76
								C-7BS1-0.27
146	m01244|Os11g13430	cDNA NB-ARC domain, putative	2790	123	6E-28	256/431 (59%)	4054	6AL8-0.90-1.00
								6BL5-0.40-1.00
								6DL10-0.80-1.00
147	m01278|Os11g13770	cDNA hypothetical protein	555	115	3E-26	205/333 (61%)	13015	4AL12-0.43-0.59
148	m01287|Os11g13850	cDNA Rieske [2Fe-2S] domain, putative	1100	473	e-134	378/448 (84%)	8324	4AL13-0.59-1.00*
								4BS
149	m01288|Os11g13850	cDNA Rieske [2Fe-2S] domain, putative	1182	458	e-129	358/423 (84%)	8324	4AL13-0.59-1.00*
								4BS
150	m01289|Os11g13850	cDNA Rieske [2Fe-2S] domain, putative	1230	458	e-129	358/423 (84%)	8324	4AL13-0.59-

								1.00*
								4BS
151	m01293|Os11g13890	cDNA chlorophyll a/b-binding protein CP26 precursor - maize	1682	741	0	552/632 (87%)	18125	4AL12-0.43-0.59
								4BS8-0.57-0.81
								4DS3-0.67-0.82
152	m01294|Os11g13890	cDNA chlorophyll a/b-binding protein CP26 precursor - maize	1250	1087	0	790/890 (88%)	18125	4AL12-0.43-0.59
								4BS8-0.57-0.81
								4DS3-0.67-0.82
153	m01295|Os11g13890	cDNA chlorophyll a/b-binding protein CP26 precursor - maize	1076	459	e-129	361/428 (84%)	18125	4AL12-0.43-0.59
								4BS8-0.57-0.81
								4DS3-0.67-0.82
154	m01307|Os11g14010	cDNA probable zinc finger protein - alfalfa	795	824	0	601/673 (89%)	18239	4AL12-0.43-0.59
								4DS3-0.67-0.82
								6AL4-0.55-0.90
								6BL5-0.40-1.00
								6DL1-0.47-0.68
155	m01310|Os11g14040	cDNA maleylacetoacetate isomerase	723	304	6E-83	423/640 (66%)	13578	5AL12-0.35-0.57
156	m01320|Os11g14140	cDNA Kelch motif, putative	1637	1120	0	914/1107 (82%)	17058	C-2AL1-0.85
								2BL2-0.36-0.50
								2DL3-0.49-0.76
								C-7AS8-0.45
								7BS1-0.27-1.00
								7DS5-0.36-0.61
157	m01330|Os11g14220	cDNA Tubulin/FtsZ family, GTPase domain, putative	1769	1786	0	1246/1366 (91%)	18176	1AL1-0.17-0.61
								1BL2-0.69-0.85
								1DL4-0.18-1.00*
								4AL12-0.43-0.59
								4DS2-0.82-1.00
								5BL14-0.75-0.79*

158	m01331|Os11g14220	cDNA Tubulin/FtsZ family, GTPase domain, putative	1750	1786	0	1246/1366 (91%)	18176	1AL1-0.17-0.61
								1BL2-0.69-0.85
								1DL4-0.18-1.00*
								4AL12-0.43-0.59
								4DS2-0.82-1.00
								5BL14-0.75-0.79*
159	m01332|Os11g14220	cDNA Tubulin/FtsZ family, GTPase domain, putative	1716	1721	0	1228/1366 (89%)	18176	1AL1-0.17-0.61
								1BL2-0.69-0.85
								1DL4-0.18-1.00*
								4AL12-0.43-0.59
								4DS2-0.82-1.00
								5BL14-0.75-0.79*
160	m01338|Os11g14280	cDNA HAT family dimerisation domain, putaive	3216	472	e-133	433/569 (76%)	9044	1AL3-0.61-1.00
								1BL2-0.69-0.85
								1DL2-0.41-1.00
161	m01350|Os11g14400	cDNA endopolygalacturonase	1170	126	3E-29	176/273 (64%)	9240	2BL4-0.50-0.89
								C4B
162	m01351|Os11g14410	cDNA Polygalacturonase pectinase	1544	115	7E-26	132/192 (68%)	9240	2BL4-0.50-0.89
								C4B
163	m01352|Os11g14420	cDNA receptor-like protein kinase	2555	605	e-173	656/921 (71%)	16094	5BL16-0.79-1.00
164	m01362|Os11g14520	cDNA hypothetical protein	1716	443	e-124	429/569 (75%)	9044	1AL3-0.61-1.00
								1BL2-0.69-0.85
								1DL2-0.41-1.00
165	m01364|Os11g14540	cDNA expressed protein	1957	513	e-145	446/569 (78%)	9044	1AL3-0.61-1.00
								1BL2-0.69-0.85
								1DL2-0.41-1.00
166	m01367|Os11g14570	cDNA expressed protein	2467	475	e-134	415/531 (78%)	9044	1AL3-0.61-1.00
								1BL2-0.69-0.85
								1DL2-0.41-1.00
167	m01368|Os11g14570	cDNA expressed protein	2389	475	e-134	415/531 (78%)	9044	1AL3-0.61-1.00

								1BL2-0.69-0.85
								1DL2-0.41-1.00
168	m01375|Os11g14630	cDNA hypothetical protein	1857	394	e-109	411/569 (72%)	9044	1AL3-0.61-1.00
								1BL2-0.69-0.85
								1DL2-0.41-1.00
169	m01402|Os11g14900	cDNA cysteine proteinase (EC 3..4.22.-) - rice	1509	524	e-149	718/1066 (67%)	14500	3AL3-0.42-0.78
								3BL2-0.22-0.50
170	m01403|Os11g14900	cDNA cysteine proteinase (EC 3..4.22.-) - rice	1440	524	e-149	718/1066 (67%)	14500	3AL3-0.42-0.78
								3BL2-0.22-0.50
171	m01404|Os11g14900	cDNA cysteine proteinase (EC 3..4.22.-) - rice	1494	524	e-149	718/1066 (67%)	14500	3AL3-0.42-0.78
								3BL2-0.22-0.50
172	m01405|Os11g14910	cDNA oxidoreductase, zinc-binding dehydrogenase family	1278	624	e-179	606/799 (75%)	12544	4AL12-0.43-0.59
								4BS8-0.57-0.81
173	m01409|Os11g14950	cDNA acetolactate synthase, small subunit, putative	1702	453	e-127	529/765 (69%)	11797	1AL1-0.17-0.61
174	m01413|Os11g14990	cDNA expressed protein	835	443	e-125	361/438 (82%)	11687	4AL12-0.43-0.59
								6BL5-0.40-1.00
175	m01481|Os11g15670	cDNA Similar to NBS-LRR disease resistance protein homologue	2658	237	2E-62	499/814 (61%)	6925	3AS4-0.45-1.00
176	m01491|Os11g16260	cDNA laccase LAC2-1	1788	172	6E-43	319/505 (63%)	8434	3BL7-0.63-1.00
177	m01498|Os11g16320	cDNA expressed protein	1300	960	0	768/916 (83%)	17011	C-1BL6-0.32
178	m01521|Os11g16530	cDNA NB-ARC domain, putative	2883	142	1E-33	252/401 (62%)	4054	6AL8-0.90-1.00
								6BL5-0.40-1.00
								6DL10-0.80-1.00
179	m01527|Os11g16590	cDNA Clp amino terminal domain, putative	3224	855	0	812/1080 (75%)	13724	1AL1-0.17-0.61
								1DL2-0.41-1.00
								2BL6-0.89-1.00
								2DL9-0.76-1.00
								C-5AS1-0.40
								C-5BS4-0.43

								C-5DS1-0.63
180	m01545|Os11g16770	cDNA ATPase, AAA family, putative	2757	664	0	758/1083 (69%)	13724	1AL1-0.17-0.61
								1DL2-0.41-1.00
								2BL6-0.89-1.00
								2DL9-0.76-1.00
								C-5AS1-0.40
								C-5BS4-0.43
								C-5DS1-0.63
181	m01559|Os11g16910	cDNA hypothetical protein	603	465	e-131	421/549 (76%)	9044	1AL3-0.61-1.00
								1BL2-0.69-0.85
								1DL2-0.41-1.00
182	m01576|Os11g17080	cDNA Protein kinase domain, putative	2227	684	0	725/1019 (71%)	14695	7AL1-0.39-0.71
183	m01608|Os11g17380	cDNA Similar to stem rust resistance protein	2082	166	5E-41	310/501 (61%)	15562	4AL12-0.43-0.59
								4BS4-0.37-0.57
								4DS3-0.67-0.82
184	m01625|Os11g17540	cDNA Dienelactone hydrolase family, putative	1103	158	7E-39	259/394 (65%)	11680	1AL1-0.17-0.61
								1BL1-0.47-0.69
								1DL2-0.41-1.00
185	m01626|Os11g17540	cDNA Dienelactone hydrolase family, putative	1044	290	2E-78	462/717 (64%)	11680	1AL1-0.17-0.61
								1BL1-0.47-0.69
								1DL2-0.41-1.00
186	m01644|Os11g17720	cDNA expressed protein	1478	152	8E-37	250/400 (62%)	8402	C-1BS10-0.50
								7BL10-0.78-1.00
187	m01677|Os11g18050	cDNA POT family	1606	153	3E-37	160/224 (71%)	4479	2BL2-0.36-0.50
								5BS8-0.56-0.71
								C-5DS1-0.63
188	m01789|Os11g19140	cDNA hypothetical protein	1116	240	1E-63	242/333 (72%)	17648	4BL5-0.86-1.00
								4DL12-0.71-1.00
								5AL23-0.87-1.00
189	m01800|Os11g19250	cDNA expressed protein	2956	242	1E-63	198/239 (82%)	9821	7DL5-0.30-0.61

190	m01821|Os11g19460	cDNA HAD-superfamily hydrolase, subfamily IA, variant 3, putative	1575	765	0	675/848 (79%)	10369	4DS2-0.82-1.00
191	m01852|Os11g19770	cDNA argininosuccinate synthase, putative	717	412	e-115	359/454 (79%)	17492	1DL2-0.41-1.00
192	m01853|Os11g19780	cDNA caffeic acid methyl transferase, putative	303	244	3E-65	225/297 (75%)	14629	4AL12-0.43-0.59
								4BS8-0.57-0.81
								4DS2-0.82-1.00
193	m01854|Os11g19790	cDNA Similar to herbicide safener binding protein 1 - maize	882	462	e-130	553/785 (70%)	14629	4AL12-0.43-0.59
								4BS8-0.57-0.81
								4DS2-0.82-1.00
194	m01855|Os11g19800	cDNA Similar to small GTP-binding protein	840	182	4E-46	156/198 (78%)	8249	3AS4-0.45-1.00
								3BS1-0.33-0.57
								3DS3-0.24-0.55
195	m01859|Os11g19840	cDNA O-methyltransferase	1253	635	0	757/1096 (69%)	17424	1DS5-0.70-1.00
								4BL5-0.86-1.00
								4DL13-0.56-0.71
196	m01863|Os11g19880	cDNA O-methyltransferase	1812	578	e-165	741/1090 (67%)	17424	1DS5-0.70-1.00
								4BL5-0.86-1.00
								4DL13-0.56-0.71
197	m01879|Os11g20040	cDNA flavonoid7-O-methyltransferase, putative	1107	557	e-159	730/1086 (67%)	17424	1DS5-0.70-1.00
								4BL5-0.86-1.00
								4DL13-0.56-0.71
198	m01883|Os11g20080	cDNA herbicide safener binding protein 1 - maize	1041	523	e-148	677/995 (68%)	17941	6AL8-0.90-1.00
								6BL5-0.40-1.00
								6DL10-0.80-1.00
199	m01884|Os11g20090	cDNA O-methyltransferase	1326	600	e-172	595/807 (73%)	14629	4AL12-0.43-0.59
								4BS8-0.57-0.81
								4DS2-0.82-1.00
200	m01891|Os11g20160	cDNA O-methyltransferase	1157	657	0	754/1076 (70%)	17424	1DS5-0.70-1.00

								4BL5-0.86-1.00
								4DL13-0.56-0.71
201	m01911|Os11g20360	cDNA Similar to receptor protein kinase-like protein	3750	355	2E-97	298/368 (80%)	7304	4AL13-0.59-0.66
								4BS1-0.81-1.00
								4DS1-0.53-0.67
202	m01912|Os11g20360	cDNA Similar to receptor protein kinase-like protein	3312	572	e-163	490/615 (79%)	7304	4AL13-0.59-0.66
								4BS1-0.81-1.00
								4DS1-0.53-0.67
203	m01946|Os11g20700	cDNA 3' exoribonuclease family, domain 2, putative	390	431	e-121	328/385 (85%)	11631	4AL12-0.43-0.59
								4BS8-0.57-0.81
								4DS3-0.67-0.82
204	m01955|Os11g20790	cDNA adenylate kinase b (EC 2.7.4.3) (atp-amp transphosphorylase)	1031	808	0	625/741 (84%)	18143	C-5AL12-0.35
								5BS4-0.43-0.56
								C-5DS1-0.63
205	m01976|Os11g21990	cDNA expressed protein	1717	1588	0	1253/1475 (84%)	18144	C-4AL12-0.43
								C-4BL1-0.71
								C-4DL9-0.31
206	m02106|Os11g23770	cDNA cysteine protease 1, putative	1419	136	4E-32	159/233 (68%)	18489	7BL10-0.78-1.00
207	m02108|Os11g23790	cDNA expressed protein	1753	738	0	558/646 (86%)	2938	5AS3-0.75-0.98
								5BS8-0.56-1.00*
208	m02109|Os11g23790	cDNA expressed protein	1571	738	0	558/646 (86%)	2938	5AS3-0.75-0.98
								5BS8-0.56-1.00*
209	m02110|Os11g23790	cDNA expressed protein	1746	738	0	558/646 (86%)	2938	5AS3-0.75-0.98
								5BS8-0.56-1.00*
210	m02111|Os11g23790	cDNA expressed protein	1666	738	0	558/646 (86%)	2938	5AS3-0.75-0.98
								5BS8-0.56-1.00*
211	m02125|Os11g23930	cDNA expressed protein	768	115	4E-26	190/288 (65%)	9444	2BL
								6BL5-0.40-1.00
212	m02139|Os11g24070	cDNA Protease inhibitor/seed storage/LTP family, putative	865	267	6E-72	261/344 (75%)	17231	3BS8-0.78-1.00

213	m02152|Os11g24200	cDNA At2g22990/T20K9.20	900	315	3E-86	348/493 (70%)	8542	C-4AL12-0.43
								C-4BS4-0.37
214	m02161|Os11g24290	cDNA serine carboxypeptidase, putative-related	690	236	2E-62	257/366 (70%)	8542	C-4AL12-0.43
								C-4BS4-0.37
215	m02166|Os11g24340	cDNA Serine carboxypeptidase	1188	131	1E-30	114/146 (78%)	8542	C-4AL12-0.43
								C-4BS4-0.37
216	m02173|Os11g24410	cDNA Serine carboxypeptidase	711	310	7E-85	334/473 (70%)	8542	C-4AL12-0.43
								C-4BS4-0.37
217	m02177|Os11g24450	cDNA 2-oxoglutarate/malate translocator (clone OMT103), mitochondrial membrane - proso millet	1366	881	0	750/940 (79%)	18719	1AS1-0.47-0.86
								1BS10-0.50-0.84
								1DS3-0.48-0.59
								3BL2-0.22-0.50
								4BS1-0.81-1.00
								C-4AL12-0.43
								4DS2-0.82-1.00
218	m02188|Os11g24560	cDNA At1g05520/T25N20_16	2778	1291	0	942/1065 (88%)	15637	4BL5-0.86-1.00
								4DL13-0.56-1.00*
219	m02235|Os11g25030	cDNA hypothetical protein	165	128	1E-30	93/106 (87%)	18719	1AS1-0.47-0.86
								1BS10-0.50-0.84
								1DS3-0.48-0.59
								3BL2-0.22-0.50
								4BS1-0.81-1.00
								C-4AL12-0.43
								4DS2-0.82-1.00
220	m02238|Os11g25060	cDNA AY085111 leucoanthocyanidin dioxygenase-like protein	1176	182	5E-46	257/400 (64%)	12963	C-2BS1-0.53
								5AS
221	m02258|Os11g25260	cDNA apyrase	1723	841	0	887/1234 (71%)	17912	4BL5-0.86-1.00
								4DL13-0.56-1.00*

								5AL23-0.87-1.00
222	m02265|Os11g25330	cDNA S-type apyrase	1421	737	0	792/1115 (71%)	17912	4BL5-0.86-1.00
								4DL13-0.56-1.00*
								5AL23-0.87-1.00
223	m02277|Os11g25450	cDNA expressed protein	1063	355	5E-98	446/664 (67%)	15946	2DL9-0.76-1.00
224	m02278|Os11g25460	cDNA hypothetical protein	513	234	4E-62	316/480 (65%)	18369	7AS5-0.59-0.89
								7BS1-0.27-1.00
								7DS4-0.61-1.00
225	m02283|Os11g25510	cDNA Protein kinase domain, putative	1524	125	1E-28	236/379 (62%)	16028	1AL3-0.61-1.00
								1BL2-0.69-1.00*
								1DL
								4AL12-0.43-0.59
								4DS1-0.53-0.67
								C-6AL4-0.55
226	m02305|Os11g25730	cDNA UDP-glucosyltransferase BX8	1533	576	e-164	788/1193 (66%)	18369	7AS5-0.59-0.89
								7BS1-0.27-1.00
								7DS4-0.61-1.00
227	m02331|Os11g25990	cDNA hypothetical protein	1515	101	1E-21	194/320 (60%)	15946	2DL9-0.76-1.00
228	m02389|Os11g26570	cDNA embryogenic-cell protein 40 (ecp40)	981	168	8E-42	187/249 (75%)	13095	6AL8-0.90-1.00
								6AS1-0.35-0.65
								6BL5-0.40-1.00
229	m02407|Os11g26750	cDNA dehydrin rab 16d	852	137	8E-33	116/146 (79%)	13095	6AL8-0.90-1.00
								6AS1-0.35-0.65
								6BL5-0.40-1.00
230	m02408|Os11g26760	cDNA dehydrin rab 16c	794	204	7E-53	266/379 (70%)	13095	6AL8-0.90-1.00
								6AS1-0.35-0.65
								6BL5-0.40-1.00
231	m02410|Os11g26780	cDNA dehydrin rab 16b	872	187	1E-47	266/385 (69%)	13095	6AL8-0.90-1.00
								6AS1-0.35-0.65
								6BL5-0.40-1.00
232	m02411|Os11g26790	cDNA probable dehydrin - rice	890	213	1E-55	224/294 (76%)	18080	6AL8-0.90-1.00
								6BL3-0.36-0.40
								6DL11-0.74-0.80

233	m02419|Os11g26860	cDNA serine hydroxymethyltransferase, putative	1896	1855	0	1311/1442 (90%)	18296	C-2AL1-0.85
234	m02425|Os11g26910	cDNA Skp1 family, dimerisation domain, putative	631	626	e-180	457/510 (89%)	17675	4DL12-0.71-1.00
								5AL23-0.87-1.00
235	m02460|Os11g27260	cDNA Similar to serine carboxypeptidase, putative	762	126	2E-29	204/324 (62%)	8542	C-4AL12-0.43
								C-4BS4-0.37
236	m02461|Os11g27260	cDNA Similar to serine carboxypeptidase, putative	955	126	2E-29	204/324 (62%)	8542	C-4AL12-0.43
								C-4BS4-0.37
237	m02465|Os11g27300	cDNA Similar to At5g08050/F13G24_250	381	264	2E-71	203/240 (84%)	2571	C-3BL2-0.22
238	m02480|Os11g27450	cDNA Leucine Rich Repeat, putative	3786	242	1E-63	320/473 (67%)	1610	7AS1-0.89-1.00
239	m02508|Os11g27730	cDNA Cytochrome P450	1925	347	2E-95	478/718 (66%)	15065	3AS4-0.45-1.00
								3BS9-0.57-0.78
								3DS6-0.55-1.00
240	m02509|Os11g27730	cDNA Cytochrome P450	1849	201	2E-51	299/457 (65%)	15065	3AS4-0.45-1.00
								3BS9-0.57-0.78
								3DS6-0.55-1.00
241	m02542|Os11g28060	cDNA Similar to cytochrome P-450 Maize	660	337	5E-93	385/543 (70%)	15065	3AS4-0.45-1.00
								3BS9-0.57-0.78
								3DS6-0.55-1.00
242	m02571|Os11g28350	cDNA CDK-activating kinase Assembly factor MAT1, putative	1133	565	e-161	501/632 (79%)	12768	7DS4-0.61-1.00
243	m02572|Os11g28360	cDNA ubiquitin carboxyl terminal hydrolase, putative	2874	729	0	593/707 (83%)	5325	C-4BL1-0.71
								C-4DL9-0.31
244	m02666|Os11g29290	cDNA Cytochrome P450	1671	148	8E-36	203/308 (65%)	730	4AL12-0.43-0.59
								4BS8-0.57-0.81
								4DS3-0.67-0.82
245	m02674|Os11g29370	cDNA 2,3-diketo-5-methylthio-1-phosphopentane phosphatase, putative	1891	1649	0	1243/1446 (85%)	17902	5AS1-0.40-0.75
								5BS4-0.43-0.56
								C-5DS1-0.63

246	m02677|Os11g29400	cDNA 6-phosphogluconate dehydrogenase, decarboxylating	1890	730	0	959/1433 (66%)	17657	2AS5-0.78-1.00
								3AL5-0.78-1.00
								3DL3-0.81-1.00
								4AL4-0.80-1.00
								4AS1-0.20-0.63
								7AS1-0.89-1.00
								7DS4-0.61-1.00
247	m02697|Os11g29600	cDNA hypothetical protein	237	153	4E-38	134/167 (80%)	15451	1BS.sat18-0.50-1.00
								3AS4-0.45-1.00
								3BS8-0.78-1.00
248	m02716|Os11g29780	cDNA At1g67330	1378	129	3E-30	99/112 (88%)	7271	3BL7-0.63-1.00
								3DL3-0.81-1.00
249	m02725|Os11g29870	cDNA probable wrky transcription factor 75 (wrky dna-binding protein 75)	796	185	4E-47	178/240 (74%)	9688	1AL3-0.61-1.00
								1BL3-0.85-1.00
250	m02774|Os11g30360	cDNA hypothetical protein	231	259	4E-70	183/203 (90%)	14062	4BL5-0.86-1.00
								4DL13-0.56-0.71
								5AL23-0.87-1.00
251	m02776|Os11g30380	cDNA SBP-domain protein 7	420	125	3E-29	165/242 (68%)	15945	C-3AL3-0.42
								C-3BL2-0.22
								3DL2-0.27-0.81
252	m02779|Os11g30410	cDNA ubiquitin activating enzyme like protein	1356	813	0	640/748 (85%)	15686	C7B
253	m02858|Os11g31190	cDNA senescence-associated protein SAG29, putative	1492	643	0	532/659 (80%)	17133	6BL5-0.40-1.00
254	m02904|Os11g31640	cDNA serine palmitoyltransferase	2113	622	e-178	563/729 (77%)	15369	3DL2-0.27-0.81
								3BL7-0.63-1.00
255	m02930|Os11g31900	cDNA acyl carrier protein, putative	1055	126	2E-29	168/247 (68%)	17481	C-7BS1-0.27
256	m02931|Os11g31900	cDNA acyl carrier protein, putative	875	126	2E-29	168/247 (68%)	17481	C-7BS1-0.27
257	m02935|Os11g31940	cDNA GDSL-like Lipase/Acylhydrolase	1311	145	5E-35	481/846 (56%)	18039	3AS
								C-3BS1-0.33
								3DS3-0.24-0.55
258	m02950|Os11g32090	cDNA Similar to zgc:56443	588	294	3E-80	245/295 (83%)	9303	C-7AS8-0.45

								7BS1-0.27-1.00
								C-7DS5-0.36
259	m02952|Os11g32110	cDNA Auxin response factor, putative	2436	326	4E-89	352/484 (72%)	8980	3BL7-0.63-1.00
								3AL5-0.78-1.00
260	m02958|Os11g32170	cDNA stripe rust resistance protein Yr10	4155	166	1E-40	276/433 (63%)	14109	2BS3-0.84-1.00
261	m02962|Os11g32210	cDNA NBS-LRR resistance protein, putative	4161	163	1E-39	270/428 (63%)	14109	2BS3-0.84-1.00
262	m02965|Os11g32240	cDNA cytochrome P450-like protein	2040	1691	0	1258/1445 (87%)	17804	4AL12-0.43-0.59
								4BS4-0.37-0.57
								4DS1-0.53-0.67
263	m02970|Os11g32290	cDNA exonuclease, putative	960	351	4E-97	459/667 (68%)	17476	3DS3-0.24-0.55
264	m02989|Os11g32480	cDNA expressed protein	874	502	e-142	464/582 (79%)	17043	2AS5-0.78-1.00
								2BS4-0.75-0.84
265	m02993|Os11g32520	cDNA Similar to auxin-regulated protein GH3 homolog F3H9.21 - Arabidopsis thaliana	1410	196	3E-50	279/430 (64%)	5995	3BL2-0.22-0.50
266	m02995|Os11g32540	cDNA Similar to chalcone synthase	636	133	2E-31	177/271 (65%)	18714	5DS2-0.78-1.00
267	m02996|Os11g32550	cDNA hypothetical protein	231	104	2E-23	83/101 (82%)	8848	1AS3-0.86-1.00
								1BS.sat19-0.31-0.50
268	m02999|Os11g32580	cDNA Chalcone and stilbene synthases, N-terminal domain, putative	1242	320	2E-87	400/589 (67%)	8848	1AS3-0.86-1.00
								1BS.sat19-0.31-0.50
269	m03002|Os11g32610	cDNA Chalcone and stilbene synthases, N-terminal domain, putative	1206	299	3E-81	400/592 (67%)	8848	1AS3-0.86-1.00
								1BS.sat19-0.31-0.50
270	m03003|Os11g32620	cDNA Chalcone and stilbene synthases, N-terminal domain, putative	1224	378	e-105	427/601 (71%)	8848	1AS3-0.86-1.00
								1BS.sat19-0.31-0.50
271	m03006|Os11g32650	cDNA Chalcone and stilbene synthases, N-terminal domain, putative	1803	278	6E-75	416/652 (63%)	18714	5DS2-0.78-1.00

272	m03056|Os11g33120	cDNA respiratory burst oxidase homolog	2931	283	4E-76	342/501 (68%)	15146	3AL3-0.42-0.78
								3BL10-0.50-0.63
								3DL2-0.27-0.81
273	m03071|Os11g33270	cDNA probable xyloglucan endotransglucosylase/hydrolase precursor(ec 2.4.1.207)	888	386	e-107	277/311 (89%)	10745	C-4AL12-0.43
274	m03074|Os11g33300	cDNA O-methyltransferase, putative	1128	524	e-149	610/875 (69%)	13223	6DS6-0.99-1.00
275	m03138|Os11g34370	cDNA At1g33270	1494	481	e-136	381/459 (83%)	10852	1AL1-0.17-0.61
								C-1BL6-0.32
								1DL2-0.41-1.00
276	m03145|Os11g34440	cDNA hypothetical protein	495	180	6E-46	194/260 (74%)	2586	4DL9-0.31-0.56
								4BL5-0.86-1.00
277	m03146|Os11g34450	cDNA GF14-d protein - rice	1454	633	0	587/770 (76%)	18638	3AL5-0.78-1.00
								3BL7-0.63-1.00
278	m03164|Os11g34630	cDNA Protein kinase domain, putative	1089	264	7E-71	350/534 (65%)	4940	1BL3-0.85-1.00
								1DL2-0.41-1.00
279	m03172|Os11g34710	cDNA purple acid phosphatase precursor, putative	1312	633	0	638/872 (73%)	15186	C-4AL12-0.43
								4BL1-0.71-1.00*
								4DL12-0.71-1.00
280	m03173|Os11g34720	cDNA Ser/Thr protein phosphatase family protein, putative	1038	367	e-102	511/771 (66%)	15186	C-4AL12-0.43
								4BL1-0.71-1.00*
								4DL12-0.71-1.00
281	m03204|Os11g35010	cDNA NB-ARC domain, putative	2913	125	2E-28	202/322 (62%)	4054	6AL8-0.90-1.00
								6BL5-0.40-1.00
								6DL10-0.80-1.00
282	m03205|Os11g35020	cDNA Yippee putative zinc-binding protein	372	206	1E-53	179/229 (78%)	11041	5AL12-0.35-0.57
283	m03206|Os11g35030	cDNA growth-regulating factor, putative	1146	120	2E-27	104/133 (78%)	11904	C-7BL2-0.33
284	m03212|Os11g35090	cDNA kinesin-like centromere protein putative	1929	588	e-168	461/547 (84%)	5326	3BL7-0.63-1.00
285	m03243|Os11g35390	cDNA myb-related protein 306 -garden snapdragon	1464	236	4E-62	249/345 (72%)	3976	6BL
								6DL6-0.29-0.68*
								C-6AL4-0.55

								C-6AS5-0.65*
								C-6DL6-0.29
286	m03262|Os11g35580	cDNA NB-ARC domain, putative	2913	271	2E-72	429/663 (64%)	6089	7AS1-0.89-1.00
287	m03275|Os11g35710	cDNA Similar to beta-amyrin synthase	2217	299	5E-81	396/604 (65%)	16219	6BS5-0.76-1.05
288	m03311|Os11g36050	cDNA probable prefoldin subunit2	444	475	e-134	373/442 (84%)	14886	2DL9-0.76-1.00
								7AS5-0.59-0.89
								7DS4-0.61-1.00
289	m03320|Os11g36140	cDNA Leucine Rich Repeat, putative	3207	101	3E-21	112/161 (69%)	6898	C-7DL5-0.30
								7AL1-0.39-0.71
290	m03340|Os11g36340	cDNA Targeting protein for Xklp2 (TPX2), putative	1965	427	e-119	426/568 (75%)	6385	C-4BL1-0.71
								C-4DL9-0.31
291	m03348|Os11g36420	cDNA At1g08660/F22O13_14	1892	147	3E-35	367/618 (59%)	12177	6BS-Sat
292	m03349|Os11g36430	cDNA Zinc finger, C3HC4 type RING finger, putative	1646	545	e-155	601/794 (75%)	9068	C-4AL12-0.43
								4BL1-0.71-0.86
								C-4DL9-0.31
293	m03350|Os11g36430	cDNA Zinc finger, C3HC4 type RING finger, putative	1647	545	e-155	601/794 (75%)	9068	4BL1-0.71-0.86
								C-4AL12-0.43
								C-4DL9-0.31
294	m03351|Os11g36430	cDNA Zinc finger, C3HC4 type RING finger, putative	1719	833	0	635/735 (86%)	17889	4AL
295	m03352|Os11g36430	cDNA Zinc finger, C3HC4 type RING finger, putative	1799	833	0	635/735 (86%)	17889	4AL
296	m03365|Os11g36560	cDNA hypothetical protein	2682	101	3E-21	132/201 (65%)	5725	C-6BS5-0.76
297	m03382|Os11g36730	cDNA Lipoxygenase	2566	989	0	1105/1557 (70%)	18029	5BL9-0.76-0.79
298	m03403|Os11g36940	cDNA Glycosyl hydrolases family 17, putative	1479	302	4E-82	372/544 (68%)	3637	6DL10-0.80-1.00
								6AL8-0.90-1.00
299	m03416|Os11g37070	cDNA hypothetical protein	1377	407	e-113	301/346 (86%)	4532	C-4BL1-0.71
								C-4DL9-0.31
								C-4AL12-0.43
300	m03417|Os11g37080	cDNA Similar to gene_id:MRG7.14	591	545	e-155	464/560 (82%)	17762	C-2AL1-0.85

								2BL6-0.89-1.00
								2DL9-0.76-1.00
								3AL3-0.42-0.78
								3BL2-0.22-0.50
								3DL2-0.27-0.81
								C-4AS1-0.20
								4BS4-0.37-0.57
								C-4DS1-0.53
301	m03428|Os11g37190	cDNA hypothetical protein	426	114	6E-26	138/200 (69%)	7811	C-6AL4-0.55
302	m03437|Os11g37280	cDNA osc6 protein - rice	863	101	8E-22	82/101 (81%)	5298	4DL13-0.56-1.00*
303	m03443|Os11g37340	cDNA F-box domain, putative	1197	104	1E-22	114/153 (74%)	10479	2AL1-0.85-1.00
304	m03460|Os11g37510	cDNA ribosomal protein L4/L1 family, putative	1219	933	0	749/895 (83%)	15105	3DL2-0.27-0.81
								3BL10-0.50-0.63
305	m03463|Os11g37540	cDNA Protein phosphatase 2C, putative	3258	502	e-142	420/499 (84%)	2206	5DL
								5AL10-0.57-0.78
								5BL9-0.76-0.79
								5DL9-0.74-0.76
306	m03464|Os11g37550	cDNA calmodulin	513	187	8E-48	286/450 (63%)	16205	1AL3-0.61-1.00
307	m03505|Os11g37920	cDNA intracellular protease, PfpI family, putative	1342	133	4E-31	136/179 (75%)	15769	2AL1-0.85-1.00
								2BL6-0.89-1.00
								2DL9-0.76-1.00
308	m03507|Os11g37940	cDNA Barwin family, putative	614	259	1E-69	279/385 (72%)	17663	1DL2-0.41-1.00
309	m03508|Os11g37950	cDNA barwin	896	439	e-123	349/422 (82%)	17663	1DL2-0.41-1.00
310	m03509|Os11g37960	cDNA Barwin family, putative	730	383	e-107	331/421 (78%)	17663	1DL2-0.41-1.00
311	m03510|Os11g37970	cDNA Barwin family	655	256	1E-68	289/412 (70%)	17663	1DL2-0.41-1.00
312	m03515|Os11g38020	cDNA Similar to GTP binding protein	1898	161	1E-39	286/426 (67%)	6301	C-4DS1-0.53
313	m03518|Os11g38050	cDNA Phosphoesterase family, putative	981	351	4E-97	434/632 (68%)	8598	3BL7-0.63-1.00
314	m03519|Os11g38060	cDNA hypothetical protein	675	220	1E-57	234/330 (70%)	17629	4AL5-0.66-0.80
								5BL16-0.79-1.00
315	m03529|Os11g38160	cDNA Sugar transporter	1569	540	e-153	827/1294 (63%)	17497	C-2AL1-0.85
								C-2BL2-0.36

								C-2DL3-0.49
316	m03530|Os11g38170	cDNA expressed protein	2113	1261	0	930/1055 (88%)	10746	1BL3-0.85-1.00
								1DL2-0.41-1.00
317	m03534|Os11g38210	cDNA hypothetical protein	429	271	3E-73	295/415 (71%)	13794	5AL12-0.35-0.57
								5BL6-0.29-0.75*
								C-5DL1-0.60
318	m03535|Os11g38220	cDNA hypothetical protein	429	288	2E-78	307/423 (72%)	13794	5AL12-0.35-0.57
								5BL6-0.29-0.75*
								C-5DL1-0.60
319	m03537|Os11g38240	cDNA expressed protein	652	253	9E-68	287/405 (70%)	13794	5AL12-0.35-0.57
								5BL6-0.29-0.75*
								C-5DL1-0.60
320	m03573|Os11g38600	cDNA kinetochore protein, putative	429	297	3E-81	225/258 (87%)	12166	2BS1-0.53-0.75
								2DS1-0.33-0.47
								C-5BL6-0.29
321	m03580|Os11g38650	cDNA UDP-glucoronosyl and UDP-glucosyl transferase	1885	275	6E-74	250/317 (78%)	4887	C-3AS2-0.23
								C-3BS1-0.33
								C-3DS3-0.24
322	m03582|Os11g38670	cDNA hypothetical protein	1872	110	2E-24	137/205 (66%)	14039	4AS3-0.76-1.00
								4BL5-0.86-1.00
323	m03595|Os11g38800	cDNA Zinc finger, C3HC4 type RING finger, putative	1504	182	7E-46	192/265 (72%)	3810	5D
324	m03596|Os11g38800	cDNA Zinc finger, C3HC4 type RING finger, putative	1500	182	7E-46	192/265 (72%)	3810	5D
325	m03601|Os11g38850	cDNA Putative receptor-like protein kinase 4	2031	576	e-164	585/792 (73%)	16250	2BS3-0.84-1.00
								C-5DL1-0.60
326	m03602|Os11g38860	cDNA Putative methyltransferase	2489	282	1E-75	335/493 (67%)	14877	C-5AL10-0.57*
								C-5BL14-0.75*
								C-5DL1-0.60
327	m03607|Os11g38910	cDNA Similar to pyruvate decarboxylase (EC 4.1.1.1) - fava bean	486	204	4E-53	204/280 (72%)	18633	2AS5-0.78-1.00
								4AS1-0.20-0.63

328	m03610|Os11g38940	cDNA ribosomal protein S4	1026	445	e-125	359/433 (82%)	18785	C-2BS1-0.53
								2DS5-0.47-1.00
329	m03611|Os11g38940	cDNA ribosomal protein S4	1007	483	e-136	359/414 (86%)	18785	C-2BS1-0.53
								2DS5-0.47-1.00
330	m03613|Os11g38960	cDNA ribosomal protein S4	965	483	e-137	359/414 (86%)	18785	C-2BS1-0.53
								2DS5-0.47-1.00
331	m03614|Os11g38960	cDNA ribosomal protein S4	956	450	e-126	351/414 (84%)	18785	C-2BS1-0.53
								2DS5-0.47-1.00
332	m03616|Os11g38980	cDNA expressed protein	2141	545	e-155	692/1026 (67%)	17198	4AS4-0.63-0.76
								4BL5-0.86-1.00
								4DL12-0.71-1.00
333	m03636|Os11g39140	cDNA At3g50960	1064	819	0	611/706 (86%)	14207	C-4DL9-0.31
								C-6BS5-0.76
								7AS5-0.59-0.89
								7BS1-0.27-1.00
								7DS4-0.61-1.00
334	m03660|Os11g39370	cDNA protein serine, putative	2546	478	e-135	436/561 (77%)	10162	7AS5-0.59-0.89
								7BS1-0.27-1.00
								7DS4-0.61-1.00
335	m03661|Os11g39370	cDNA protein serine, putative	2002	212	8E-55	164/195 (84%)	10162	7AS5-0.59-0.89
								7BS1-0.27-1.00
								7DS4-0.61-1.00
336	m03666|Os11g39420	cDNA Jacalin-like lectin domain, putative	2205	134	2E-31	250/416 (60%)	14109	2BS3-0.84-1.00
337	m03669|Os11g39450	cDNA Protein kinase domain, putative	1491	213	2E-55	442/715 (61%)	15562	4AL12-0.43-0.59
								4BS4-0.37-0.57
								4DS3-0.67-0.82
338	m03673|Os11g39490	cDNA Jacalin-like lectin domain, putative	2514	172	8E-43	402/676 (59%)	15562	4AL12-0.43-0.59
								4BS4-0.37-0.57
								4DS3-0.67-0.82
339	m03678|Os11g39540	cDNA Similar to 14-3-3 protein	693	123	1E-28	141/200 (70%)	18638	3AL5-0.78-1.00
								3BL7-0.63-1.00
340	m03689|Os11g39650	cDNA probable protein transport protein SEC12p [imported]	1608	364	e-100	334/429 (77%)	2684	C-4AL12-0.43
341	m03724|Os11g39990	cDNA patatin-like protein	1618	1093	0	957/1215	13614	C-2BL2-0.36

						(78%)		
								C-2DL3-0.49
342	m03725|Os11g40000	cDNA Similar to patatin-like protein 2	381	347	4E-96	269/320 (84%)	13614	C-2BL2-0.36
								C-2DL3-0.49
343	m03727|Os11g40020	cDNA Similar to patatin-like protein	1116	705	0	664/874 (75%)	13614	C-2BL2-0.36
								C-2DL3-0.49
344	m03732|Os11g40070	cDNA expressed protein	1629	250	2E-66	324/491 (65%)	9369	7AS5-0.59-0.89
								7BS1-0.27-1.00
								7DS4-0.61-1.00
345	m03733|Os11g40070	cDNA expressed protein	1532	323	2E-88	451/694 (64%)	9369	7AS5-0.59-0.89
								7BS1-0.27-1.00
								7DS4-0.61-1.00
346	m03734|Os11g40070	cDNA expressed protein	1516	337	1E-92	453/694 (65%)	9369	7AS5-0.59-0.89
								7BS1-0.27-1.00
								7DS4-0.61-1.00
347	m03741|Os11g40140	cDNA proteasome subunit alpha type 5 (EC 3.4.25.1)	1187	909	0	655/732 (89%)	17391	C-4AL12-0.43
								C-4BL1-0.71
								C-4DL9-0.31
348	m03742|Os11g40140	cDNA proteasome subunit alpha type 5 (EC 3.4.25.1)	1180	885	0	650/732 (88%)	17391	C-4AL12-0.43
								C-4BL1-0.71
								C-4DL9-0.31
349	m03748|Os11g40200	cDNA expressed protein	1342	484	e-137	404/498 (81%)	9475	5AL10-0.57-0.78
350	m03779|Os11g40510	cDNA oligouridylate binding protein, putative	1883	549	e-156	734/1092 (67%)	18217	7DL2-0.61-0.82
351	m03790|Os11g40590	cDNA expressed protein	3046	274	3E-73	355/508 (69%)	11406	3BS8-0.78-1.00
								3DS6-0.55-1.00
								7BL10-0.78-1.00
								7DL2-0.61-0.82
352	m03791|Os11g40600	cDNA differentially expressed osmotic protein ODE1	594	206	2E-53	187/240 (77%)	9234	3AL5-0.78-1.00
								3BL7-0.63-1.00
								3DL3-0.81-1.00
353	m03798|Os11g40660	cDNA hypothetical protein	186	158	1E-39	115/131 (87%)	8624	4AS1-0.20-0.63

								4DL9-0.31-0.56
								C-4BL1-0.71
354	m03801|Os11g40690	cDNA oxidoreductase, zinc-binding dehydrogenase family	1032	773	0	626/765 (81%)	16277	3AL5-0.78-1.00
								3BL7-0.63-1.00
								3DL3-0.81-1.00
355	m03845|Os11g41130	cDNA vacuolar sorting protein-like; embryogenesis protein H beta 58-like protein	1548	1079	0	808/936 (86%)	18151	C4A
								C-4DS1-0.53
								C-4BS4-0.37
356	m03848|Os11g41150	cDNA expressed protein	774	142	3E-34	125/161 (77%)	18114	2DS
								C-2AS5-0.78
								C-2BS4-0.75*
								C-2BS1-0.53
357	m03849|Os11g41160	cDNA phosphoserine phosphatase SerB, putative	1282	863	0	740/926 (79%)	13554	2BS1-0.53-0.75
								C-3AS2-0.23
								7AS5-0.59-0.89
358	m03894|Os11g41600	cDNA expressed protein	1107	164	9E-41	137/166 (82%)	6192	4BS8-0.57-0.81
								4DS2-0.82-1.00
359	m03895|Os11g41610	cDNA ribosomal protein S14p/S29e	613	237	5E-63	166/178 (93%)	18382	C-5AL10-0.57*
								C-5BL14-0.75*
								C-5DL1-0.60
360	m03899|Os11g41650	cDNA adenylylsulfate kinase, putative	915	456	e-128	463/639 (72%)	14655	4AS4-0.63-0.76
								4BL5-0.86-1.00
361	m03902|Os11g41680	cDNA Cytochrome P450	1539	172	5E-43	304/485 (62%)	11098	C-6BL3-0.36
362	m03905|Os11g41710	cDNA Cytochrome P450	1913	177	2E-44	314/497 (63%)	11098	C-6BL3-0.36
363	m03917|Os11g41830	cDNA sugar transporter, putative	1416	301	1E-81	383/567 (67%)	13040	4AS3-0.76-1.00
								4BL5-0.86-1.00
								4DL13-0.56-0.71
364	m03918|Os11g41840	cDNA sorbitol transporter	1437	307	1E-83	388/573 (67%)	13040	4AS3-0.76-1.00
								4BL5-0.86-1.00
								4DL13-0.56-0.71
365	m03919|Os11g41850	cDNA sugar transporter protein	1431	328	8E-90	392/573 (68%)	13040	4AS3-0.76-1.00

								4BL5-0.86-1.00
								4DL13-0.56-0.71
366	m03921|Os11g41870	cDNA sorbitol transporter	1635	302	4E-82	384/573 (67%)	13040	4AS3-0.76-1.00
								4BL5-0.86-1.00
								4DL13-0.56-0.71
367	m03924|Os11g41900	cDNA Similar to Z29518-acyl-glycerol-3-phosphate acyltransferase	1116	684	0	517/603 (85%)	17565	5AS1-0.40-0.75
								5BS4-0.43-0.56
								C-5DS1-0.63
368	m03925|Os11g41910	cDNA Similar to GTP-binding protein	2139	123	4E-28	94/111 (84%)	16082	4AS1-0.20-0.63
369	m03965|Os11g42290	cDNA Transferase family	1338	377	e-104	333/429 (77%)	10634	4AS3-0.76-1.00
								4BL5-0.86-1.00
370	m03974|Os11g42370	cDNA Transferase family	1584	367	e-101	330/429 (76%)	10634	4AS3-0.76-1.00
								4BL5-0.86-1.00
371	m03976|Os11g42390	cDNA serine carboxypeptidase I, putative	1389	164	1E-40	227/341 (66%)	8542	C-4AL12-0.43
								C-4BS4-0.37
372	m03989|Os11g42500	cDNA Dirigent-like protein	766	164	6E-41	191/279 (68%)	14799	C-4BL1-0.71
								4DL13-0.56-0.71
373	m04094|Os11g43510	cDNA triacylglycerol lipase	1413	358	7E-99	448/671 (66%)	15135	2AS5-0.78-1.00
								2DS5-0.47-1.00
374	m04095|Os11g43520	cDNA Glutaredoxin	327	125	2E-29	208/323 (64%)	14277	C-2AL1-0.85
								2DL
								2BL2-0.36-0.50
375	m04119|Os11g43750	cDNA polygalacturonase, putative	2065	397	e-110	671/1092 (61%)	17414	4AL5-0.66-0.80
								5BL16-0.79-1.00
								5DL5-0.76-1.00
376	m04140|Os11g43950	cDNA expressed protein	3322	748	0	723/945 (76%)	12591	5AS1-0.40-0.75
								5BS4-0.43-0.56
								C-5DS1-0.63
377	m04141|Os11g43960	cDNA ADP/ATP translocase-like protein	1599	435	e-122	311/348 (89%)	5112	3AS4-0.45-1.00
								3BS8-0.78-1.00
								3DS6-0.55-1.00
378	m04143|Os11g43970	cDNA expressed protein	4223	757	0	684/891 (76%)	13968	2AS5-0.78-1.00
								2BS1-0.53-0.75

								2DS5-0.47-1.00
379	m04144|Os11g43980	cDNA Peroxidase, putative	1346	204	1E-52	353/568 (62%)	3409	7DS4-0.61-1.00
380	m04212|Os11g44660	cDNA Protein kinase domain, putative	1371	190	2E-48	310/481 (64%)	16250	2BS3-0.84-1.00
								C-5DL1-0.60
381	m04225|Os11g44790	cDNA Similar to SNF8 like protein	831	342	2E-94	258/301 (85%)	8945	5AL10-0.57-0.78
								5BL16-0.79-1.00
382	m04233|Os11g44860	cDNA Protein kinase domain, putative	1787	174	2E-43	270/421 (64%)	13637	7BL7-0.63-0.78
383	m04234|Os11g44870	cDNA expressed protein	1883	521	e-148	672/936 (71%)	13637	7BL7-0.63-0.78
384	m04235|Os11g44870	cDNA expressed protein	1407	521	e-148	672/936 (71%)	13637	7BL7-0.63-0.78
385	m04239|Os11g44910	cDNA DNA Helicase	2063	331	1E-90	276/344 (80%)	6662	4AL12-0.43-0.59
								C-4BS4-0.37
386	m04242|Os11g44940	cDNA At3g62580/T12C14_280	975	278	3E-75	245/305 (80%)	11664	C-5AS1-0.40
387	m04285|Os11g45330	cDNA NB-ARC domain, putative	2718	258	2E-68	426/660 (64%)	6089	7AS1-0.89-1.00
388	m04292|Os11g45400	cDNA Acyltransferase, putative	1641	291	9E-79	401/604 (66%)	12354	2BS1-0.53-0.75
								2DS5-0.47-1.00
								6AL8-0.90-1.00
389	m04306|Os11g45540	cDNA Protein kinase domain, putative	1878	702	0	749/1046 (71%)	16250	C-5DL1-0.60
								2BS3-0.84-1.00
390	m04311|Os11g45590	cDNA U2 snRNP auxilliary factor, large subunit, splicing factor, putative	1842	1293	0	919/1023 (89%)	15993	C-4AL12-0.43
								C-4DS1-0.53
391	m04329|Os11g45740	cDNA Myb-like DNA-binding domain, putative	1389	163	3E-40	197/292 (67%)	3976	C-6AL4-0.55
								C-6AS5-0.65*
								6BL
								6DL6-0.29-0.68*
								C-6DL6-0.29
392	m04330|Os11g45750	cDNA NB-ARC domain, putative	4259	166	1E-40	297/485 (61%)	4026	4AL12-0.43-0.59
								4DS3-0.67-0.82
393	m04367|Os11g46100	cDNA hypothetical protein	1584	129	4E-30	215/339 (63%)	10032	4AL4-0.80-1.00
								7DS4-0.61-1.00
394	m04378|Os11g46210	cDNA NB-ARC domain, putative	3778	240	4E-63	278/385 (72%)	10032	4AL4-0.80-1.00
								7DS4-0.61-1.00
395	m04420|Os11g47120	cDNA hypothetical protein	1164	1046	0	766/863 (88%)	18131	4BS4-0.37-0.57

396	m04442|Os11g47330	cDNA expressed protein	1860	1226	0	873/973 (89%)	18131	4BS4-0.37-0.57
397	m04443|Os11g47330	cDNA expressed protein	1659	1605	0	1151/1290 (89%)	18131	4BS4-0.37-0.57
398	m04449|Os11g47390	cDNA diphenol oxidase	1752	242	6E-64	390/598 (65%)	13628	2BS
								3DL2-0.27-0.81
								C-3BL10-0.50*
399	m04456|Os11g47460	cDNA Myb-related transcription factor-like protein	923	600	e-172	557/706 (78%)	13842	C-4AL12-0.43
								C-4DS1-0.53
400	m04457|Os11g47460	cDNA Myb-related transcription factor-like protein	1199	600	e-172	557/706 (78%)	13842	C-4AL12-0.43
								C-4DS1-0.53
401	m04458|Os11g47460	cDNA Myb-related transcription factor-like protein	1201	797	0	729/921 (79%)	13842	C-4AL12-0.43
								C-4DS1-0.53
402	m04483|Os11g47710	cDNA SNF7 family, putative	1087	805	0	601/695 (86%)	17946	4AL4-0.80-1.00
								4BS4-0.37-0.57
								C-4AL12-0.43
403	m04489|Os11g47760	cDNA dnaK-type molecular chaperone hsp70 - rice	2395	743	0	573/673 (85%)	16068	2BL2-0.36-0.50
								3AL3-0.42-0.78
								3BL10-0.50-0.63
								3DL2-0.27-0.81
								C-4BL1-0.71
								4DL9-0.31-0.56
								5DL5-0.76-1.00
								6BL5-0.40-1.00
404	m04490|Os11g47760	cDNA dnaK-type molecular chaperone hsp70 - rice	2096	743	0	573/673 (85%)	16068	2BL2-0.36-0.50
								3AL3-0.42-0.78
								3BL10-0.50-0.63
								3DL2-0.27-0.81
								C-4BL1-0.71
								4DL9-0.31-0.56
								5DL5-0.76-1.00

								6BL5-0.40-1.00
405	m04495|Os11g47810	cDNA Similar to metallothionein-like protein type 1	378	141	4E-34	140/177 (79%)	16897	1AL1-0.17-0.61
								1BL1-0.47-0.69
								1DL2-0.41-1.00
406	m04496|Os11g47820	cDNA glucan endo-1,3-beta-glucosidase precursor (EC 3.2.1.39) ((1-3)-beta-glucanendohydrolase) ((1-3)-beta-glucanase) (beta-1,3-endoglucanase)	1742	1366	0	1129/1382 (81%)	17016	C-4DS1-0.53
407	m04497|Os11g47830	cDNA RNA recognition motif, putative	1775	242	6E-64	269/376 (71%)	16886	5AS1-0.40-0.75
								C-5AS3-0.75*
								5BS4-0.43-0.56
								C-5DS1-0.63
408	m04498|Os11g47840	cDNA Rhomboid family, putative	1423	285	6E-77	414/639 (64%)	13880	C-2AL1-0.85
								2BS1-0.53-0.75
409	m04511|Os11g47970	cDNA ATPase, AAA family, putative	1745	722	0	585/700 (83%)	16892	C-4AL12-0.43
								4BS4-0.37-0.57
								C-4DS1-0.53
410	m04512|Os11g47970	cDNA ATPase, AAA family, putative	1839	722	0	585/700 (83%)	16892	C-4AL12-0.43
								4BS4-0.37-0.57
								C-4DS1-0.53
411	m04513|Os11g47980	cDNA Similar to ribulose bisphosphate carboxylase/oxygenase activase b, chloroplast precursor (rubisco activase b) (rab) - [barley]	1101	193	2E-49	265/379 (69%)	16892	C-4AL12-0.43
								4BS4-0.37-0.57
								C-4DS1-0.53
412	m04517|Os11g48020	cDNA sterol4-alpha-methyl-oxidase, putative	1268	1057	0	813/945 (86%)	16935	C-4AL12-0.43
								4BS4-0.37-0.57
								C-4DS1-0.53
413	m04518|Os11g48030	cDNA expressed protein	714	348	2E-96	274/324 (84%)	13477	C-4AL12-0.43
								4BS4-0.37-0.57
								C-4DS1-0.53
414	m04519|Os11g48040	cDNA Mitochondrial carrier protein, putative	1338	1050	0	802/932 (86%)	18050	C-4AL12-0.43

								4BS4-0.37-0.57
								C-4DS1-0.53
415	m04521|Os11g48060	cDNA laccase (EC 1.10.3.2) precursor - common tobacco	1994	218	9E-57	285/433 (65%)	13628	2BS
								3DL2-0.27-0.81
								C-3BL10-0.50*
416	m04529|Os11g48110	cDNA phenylalanine ammonia-lyase	2103	1580	0	1571/2091 (75%)	18718	6BL3-0.36-0.40
								6DL6-0.29-0.47
								C-6AL4-0.55

Table 12. Complete list of 552 rice genes predicted from the sequence of chromosome 12, showing homology with the bin-mapped wheat EST contigs at a cut off bit score of 100. The gene functions have been annotated using NCBI BLASTX search and recording the top hits.

S. No.	Rice gene information			BLAST search results				Mapped wEST	
	Gene model/Locus	Predicted cDNA function	Predicted cDNA length	score	e-value	Identities	%	contig no	Bin
1	m00017|LOC_Os12g01170	hypothetical protein	861	767	0.0	583/673	86	12025	C-5BL14-0.75*
									C-5AL10-0.57*
2	m00019|LOC_Os12g01190	hypothetical protein	3330	535	e-152	447/552	80	10101	5BL
3	m00037|LOC_Os12g01360	expressed protein	3854	673	0.0	621/798	77	8417	5AL12-0.35-0.57
									5BL1-0.55-0.75
									C-5DL1-0.60
4	m00038|LOC_Os12g01370	probable omega-3 fatty acid desaturase	1809	100	5e-21	196/320	61	12010	6DL1-0.47-0.68
									6BL5-0.40-1.00
									6AL4-0.55-0.90
5	m00039|LOC_Os12g01380	expressed protein	1714	240	2e-63	340/524	64	17623	1DL
									1AL1-0.17-0.61
									1BL1-0.47-0.69
									1AL1-0.17-0.61
									1BL1-0.47-0.69
6	m00040|LOC_Os12g01390	clathrin heavy chain,putative	5605	2017	0.0	1679/1991	84	16778	4BS4-0.37-0.57
7	m00043|LOC_Os12g01420	Ammonium Transporter Family	1377	126	3e-29	275/446	61	4027	3DL2-0.27-0.81
									3AL3-0.42-0.78
									7AS1-0.89-1.00
									3BL10-0.50-1.00*
8	m00044|LOC_Os12g01430	ribosomal protein L10,putative	1347	789	0.0	596/690	86	18290	C-5AL10-0.57*
									1AL3-0.61-1.00
									6DS2-0.45-0.79
9	m00052|LOC_Os12g01510	expressed protein	2563	841	0.0	823/1086	75	14678	1AS3-0.86-1.00

									C-5BL14-0.75*
10	m00053|LOC_Os12g01510	expressed protein	2756	456	e-128	386/485	79	14678	1AS3-0.86-1.00
									C-5BL14-0.75*
11	m00059|LOC_Os12g01550	Similar to lob domain protein 12	693	202	2e-52	216/305	70	14992	C-4DS1-0.53
									4BS4-0.37-0.57
12	m00060|LOC_Os12g01560	expressed protein	1859	171	2e-42	252/397	63	17623	1DL
									1AL1-0.17-0.61
									1BL1-0.47-0.69
									1AL1-0.17-0.61
									1BL1-0.47-0.69
13	m00074|LOC_Os12g01680	light-inducible proteinATLS1	516	415	e-116	286/311	91	18382	C-5AL10-0.57*
									C-5BL14-0.75*
									C-5DL1-0.60
14	m00075|LOC_Os12g01690	PRA1 family protein,putative	663	183	9e-47	281/443	63	10949	5BL9-0.76-0.79
									5DL5-0.76-1.00
15	m00076|LOC_Os12g01700	Similar to Seq ID:rhg1_peking_amplicon	2385	109	9e-24	151/234	64	18170	3D
									3BL2-0.22-0.50
									3AL3-0.42-0.78
16	m00079|LOC_Os12g01730	Multicopper oxidase,putative	1973	527	e-150	443/544	81	8434	3BL7-0.63-1.00
17	m00080|LOC_Os12g01740	Protein kinase domain,putative	2820	370	e-102	343/448	76	11579	3BS1-0.33-0.57
									3A
									3BS
18	m00082|LOC_Os12g01760	F-box family proteinAtFBL3, putative	2631	778	0.0	636/777	81	8733	C-5BL14-0.75*
19	m00083|LOC_Os12g01760	F-box family proteinAtFBL3, putative	2465	817	0.0	679/838	81	8733	C-5BL14-0.75*
20	m00098|LOC_Os12g01910	hypothetical protein	2664	179	1e-44	173/234	73	11579	3BS1-0.33-0.57
									3A
									3BS
21	m00114|LOC_Os12g02060	Peroxidase	876	221	4e-58	337/530	63	13267	4AS1-0.20-0.63
									C-4BL1-0.71
									4DL9-0.31-0.56
									4DL9-0.31-0.56
22	m00116|LOC_Os12g02080	peroxidase	1318	247	2e-65	370/585	63	13267	4AS1-0.20-0.63
									C-4BL1-0.71
									4DL9-0.31-0.56

									4DL9-0.31-0.56
23	m00124|LOC_Os12g02150	expressed protein	909	220	1e-57	228/303	75	11885	4AL
24	m00129|LOC_Os12g02200	protein kinase homolog -rice	2018	1500	0.0	1333/1685	79	17610	C-5DL1-0.60
									7BS1-0.27-1.00
									C-5AL12-0.35
									5BL6-0.29-0.55
25	m00138|LOC_Os12g02290	nonspecific lipid-transfer protein 5 precursor (ltp 5).	321	274	3e-74	237/297	79	18722	4BS4-0.37-0.57
									4AL12-0.43-0.59
									4DS1-0.53-0.67
26	m00139|LOC_Os12g02300	Protease inhibitor/seed storage/LTP family, putative	746	174	8e-44	235/356	66	18810	2DS1-0.33-0.47
									2BS1-0.53-0.75
									2BS1-0.53-0.75
27	m00140|LOC_Os12g02310	nonspecific lipid-transferprotein 2 precursor (ltp 2).	793	206	2e-53	238/332	71	17231	3BS8-0.78-1.00
									3BS8-0.78-1.00
									3BS8-0.78-1.00
									3BS8-0.78-1.00
									3BS8-0.78-1.00
									3BS8-0.78-1.00
28	m00141|LOC_Os12g02320	Protease inhibitor/seedstorage/LTP family, putative	832	240	8e-64	254/342	74	17231	3BS8-0.78-1.00
									3BS8-0.78-1.00
									3BS8-0.78-1.00
									3BS8-0.78-1.00
									3BS8-0.78-1.00
									3BS8-0.78-1.00
29	m00142|LOC_Os12g02330	Protease inhibitor/seedstorage/LTP family, putative	853	240	8e-64	271/376	72	18810	2DS1-0.33-0.47
									2BS1-0.53-0.75
									2BS1-0.53-0.75
30	m00143|LOC_Os12g02340	Protease inhibitor/seedstorage/LTP family, putative	809	242	3e-64	251/340	73	18810	2DS1-0.33-0.47
									2BS1-0.53-0.75

									2BS1-0.53-0.75
31	m00149|LOC_Os12g02380	Elongation factor P (EF-P),putative	1785	689	0.0	500/566	88	9961	C-4BS4-0.37
									C-4BS4-0.37
									4DS1-0.53-0.67
32	m00150|LOC_Os12g02390	outer membrane protein,OMP85 family, putative	3969	885	0.0	869/1170	74	15236	C-4BL1-0.71
									4DL9-0.31-0.56
									4AS4-0.63-0.76
33	m00155|LOC_Os12g02430	hypothetical protein	294	128	2e-30	110/140	78	3976	6BL
									6BL
									C-6DL6-0.29
									C-6AL4-0.55
34	m00169|LOC_Os12g02570	Similar to expressedprotein-like protein	2509	484	e-137	490/660	74	1723	4BS4-0.37-0.57
									4DS1-0.53-0.67
									4DS1-0.53-0.67
									4AL12-0.43-0.59
35	m00175|LOC_Os12g02630	cytochrome P450monooxygenase CYP72A5	1152	101	1e-21	245/422	58	9825	6BS
									6AS5-0.65-1.00
									6AS5-0.65-1.00
									6DS6-0.99-1.00
36	m00197|LOC_Os12g02810	protein kinase, putative	1538	477	e-134	344/388	88	4367	C-5BL14-0.75*
									C-5AL10-0.57*
									C-5DL1-0.60
37	m00198|LOC_Os12g02820	retrotransposon protein,putative, unclassified	4355	247	5e-65	254/353	71	11242	2AL
38	m00213|LOC_Os12g02960	glutathione S-transferaseGST 14	597	128	4e-30	152/224	67	9824	1DS5-0.70-1.00
									1DS5-0.70-1.00
									1AS1-0.47-0.86
									1AS1-0.47-0.86
									1DS5-0.70-1.00
									1BS.sat18-0.50-1.00
39	m00215|LOC_Os12g02980	nod factor bindinglectin-nucleotide phosphohydrolase	1592	800	0.0	800/1081	74	17912	5AL23-0.87-1.00

									4BL5-0.86-1.00
									4DL13-0.56-1.00*
									4DL13-0.56-1.00*
40	m00216|LOC_Os12g02980	nod factor bindinglectin-nucleotide phosphohydrolase	1704	922	0.0	957/1313	72	17912	5AL23-0.87-1.00
									4BL5-0.86-1.00
									4DL13-0.56-1.00*
									4DL13-0.56-1.00*
41	m00221|LOC_Os12g03030	Similar to S-type apyrase	531	164	4e-41	142/181	78	17912	5AL23-0.87-1.00
									4BL5-0.86-1.00
									4DL13-0.56-1.00*
									4DL13-0.56-1.00*
42	m00223|LOC_Os12g03050	hypothetical protein	1191	112	5e-25	124/178	69	4891	7DL2-0.61-0.82
									4DS2-0.82-1.00
									4AL12-0.43-0.59
									4BS1-0.81-1.00
									2DS5-0.47-1.00
									2AS5-0.78-1.00
									7BL7-0.63-0.78
									7DL2-0.61-0.82
43	m00224|LOC_Os12g03060	expressed protein	1371	741	0.0	656/830	79	17274	C-5BL14-0.75*
									C5D
									C5B
44	m00231|LOC_Os12g03100	Exostosin family, putative	1854	129	5e-30	203/325	62	8838	3DL2-0.27-0.81
									3DL2-0.27-0.81
									3DL2-0.27-0.81
									3BL10-0.50-0.63
									3BL10-0.50-0.63
									3BL10-0.50-0.63
									3BL10-0.50-0.63

									C-5BL14-0.75*
45	m00236|LOC_Os12g03150	myb protein homolog - rice	1279	258	7e-69	271/380	71	3976	6BL
									6BL
									C-6DL6-0.29
									C-6AL4-0.55
46	m00247|LOC_Os12g03260	MATE efflux family protein,putative	1752	163	4e-40	219/336	65	11753	3AL5-0.78-1.00
									3BL7-0.63-1.00
									3DL3-0.81-1.00
									3BL7-0.63-1.00
									3DL3-0.81-1.00
									3DL3-0.81-1.00
47	m00249|LOC_Os12g03270	expressed protein	1321	198	1e-50	337/550	61	1957	
48	m00250|LOC_Os12g03270	expressed protein	1569	198	1e-50	337/550	61	1957	
49	m00251|LOC_Os12g03290	Similar toaintegumaenta-like protein - Arabidopsis thaliana	1571	150	2e-36	231/363	63	17427	3DL2-0.27-0.81
									3BL10-0.50-0.63
									3AL3-0.42-0.78
50	m00256|LOC_Os12g03340	hypothetical protein	1953	529	e-150	568/768	73	8419	C-5BL14-0.75*
									C-5AL10-0.57*
									5D
									C-5AL10-0.57*
									C-4AL12-0.43
									4BS4-0.37-0.57
									4DS1-0.53-0.67
51	m00268|LOC_Os12g03460	SET domain protein 105	2037	231	1e-60	182/219	83	8560	4DS3-0.67-0.82
									C-4BS4-0.37
									C-4AL12-0.43
									C-4AL12-0.43
52	m00269|LOC_Os12g03470	arabinoxylanarabinofuranohydrolase isoenzyme AXAH-II	2850	828	0.0	676/815	82	14869	C-5BL14-0.75*
									C-5AL10-0.57*
53	m00270|LOC_Os12g03470	arabinoxylanarabinofuranohydrolase isoenzyme AXAH-II	2711	828	0.0	676/815	82	14869	C-5BL14-0.75*

									C-5AL10-0.57*
54	m00271|LOC_Os12g03470	arabinoxylanarabinofuranohydrolase isoenzyme AXAH-II	2547	828	0.0	676/815	82	14869	C-5BL14-0.75*
									C-5AL10-0.57*
55	m00272|LOC_Os12g03480	Similar to arabinoxylanarabinofuranohydrolase isoenzyme AXAH-I	2770	462	e-130	438/580	75	14869	C-5BL14-0.75*
									C-5AL10-0.57*
56	m00277|LOC_Os12g03530	Similar to arabinoxylanarabinofuranohydrolase isoenzyme AXAH-I	2117	421	e-118	407/544	74	14869	C-5BL14-0.75*
									C-5AL10-0.57*
57	m00280|LOC_Os12g03560	hypothetical protein	1170	103	4e-22	122/175	69	18580	C-4BL1-0.71
58	m00297|LOC_Os12g03730	DHHC zinc finger domain,putative	2073	150	3e-36	146/198	73	530	7A
59	m00305|LOC_Os12g03810	probable serine/threoninekinase (EC 2.7.1.-) SNFL1 - sorghum	1323	575	e-164	658/954	68	17053	4BL5-0.86-1.00
									4DL12-0.71-1.00
									5AL23-0.87-1.00
									4BL5-0.86-1.00
60	m00306|LOC_Os12g03810	probable serine/threoninekinase (EC 2.7.1.-) SNFL1 - sorghum	1808	668	0.0	818/1205	67	17053	4BL5-0.86-1.00
									4DL12-0.71-1.00
									5AL23-0.87-1.00
									4BL5-0.86-1.00
61	m00307|LOC_Os12g03820	WD-40 repeat protein,putative	1830	511	e-145	397/467	85	16831	C-4AL12-0.43
									C-4BS4-0.37
									4DS1-0.53-0.67
62	m00313|LOC_Os12g03880	ribosomal protein L10,putative	963	1134	0.0	843/961	87	18665	C7A
									C4A
63	m00322|LOC_Os12g03970	Protein kinase domain,putative	1626	703	0.0	891/1334	66	18270	C-2AS5-0.78
									C-2BS1-0.53
									C-2DS1-0.33
64	m00335|LOC_Os12g04100	Cytochrome P450	1557	182	7e-46	222/330	67	13305	3BS9-0.57-0.78
									3BS9-0.57-0.78
									3DS6-0.55-1.00

									3BS9-0.57-0.78
									3DS6-0.55-1.00
									3AS4-0.45-1.00
65	m00336|LOC_Os12g04110	Cytochrome P450, putative	4788	194	3e-49	226/330	68	13305	3BS9-0.57-0.78
									3BS9-0.57-0.78
									3DS6-0.55-1.00
									3BS9-0.57-0.78
									3DS6-0.55-1.00
									3AS4-0.45-1.00
66	m00342|LOC_Os12g04170	hypothetical protein	1287	757	0.0	604/726	83	15785	1DL2-0.41-1.00
									1BL1-0.47-1.00*
67	m00362|LOC_Os12g04360	EF hand, putative	795	199	2e-51	191/242	78	16191	3AL5-0.78-1.00
68	m00391|LOC_Os12g04640	hexose transporter-likeprotein	1224	424	e-119	402/532	75	17497	C-2DL3-0.49
									C-2BL2-0.36
									C-2AL1-0.85
69	m00404|LOC_Os12g04750	expressed protein	2346	137	2e-32	242/388	62	8273	C-5BL9-0.76*
									6BL5-0.40-1.00
									5DL1-0.60-0.74
									6BL5-0.40-1.00
									5DL1-0.60-0.74
									5DL1-0.60-0.74
									5AL10-0.57-0.78
									5AL10-0.57-0.78
									5BL14-0.75-0.76
70	m00407|LOC_Os12g04780	Similar to F2E2.12	945	131	8e-31	94/106	88	14161	6AL4-0.55-0.90
									6DL1-0.47-0.68
									6BL5-0.40-1.00
71	m00422|LOC_Os12g04920	Ubiquitin family, putative	837	324	4e-89	215/226	95	18275	C-5AL10-0.57*
									C-5BL14-0.75*
									4DS1-0.53-0.67
									4DS1-0.53-0.67
72	m00432|LOC_Os12g05000	At1g03250/F15K9_13	1025	465	e-131	404/490	82	15108	5AL12-0.35-0.57
									5BL1-0.55-0.75
									C-5DL1-0.60

									5BL1-0.55-0.75
									5AL12-0.35-0.57
									5BL1-0.55-0.75
									C-5DL1-0.60
73	m00433|LOC_Os12g05000	At1g03250/F15K9_13	1032	464	e-131	380/453	83	15108	5AL12-0.35-0.57
									5BL1-0.55-0.75
									C-5DL1-0.60
									5BL1-0.55-0.75
									5AL12-0.35-0.57
									5BL1-0.55-0.75
									C-5DL1-0.60
74	m00437|LOC_Os12g05040	heavy metal-associateddomain, putative	2287	122	1e-27	137/198	69	16955	5BS5-0.71-0.81
									5BS5-0.71-0.81
									5BS5-0.71-0.81
									5DS5-0.67-0.78
75	m00438|LOC_Os12g05050	aluminium-induced protein,putative	1201	743	0.0	604/735	82	17620	C-7BS1-0.27
									7DS5-0.36-0.61
76	m00444|LOC_Os12g05110	Pyruvate kinase, barreldomain	2182	1922	0.0	1489/1736	85	18758	3BL7-0.63-1.00
									1BS10-0.50-0.84
77	m00445|LOC_Os12g05110	Pyruvate kinase, barreldomain	2184	1911	0.0	1489/1738	85	18758	3BL7-0.63-1.00
									1BS10-0.50-0.84
78	m00446|LOC_Os12g05120	Similar to probablereceptor-like protein kinase	2387	153	4e-37	240/364	65	7991	7AL1-0.39-0.71
									7AL1-0.39-0.71
79	m00454|LOC_Os12g05200	expressed protein	1047	144	1e-34	222/349	63	17057	C-1BL6-0.32
									1AL1-0.17-0.61
									1DL2-0.41-1.00
80	m00476|LOC_Os12g05420	IQ calmodulin-bindingmotif, putative	1853	1307	0.0	1018/1207	84	15476	7AL1-0.39-0.71
									6DS2-0.45-0.79
81	m00478|LOC_Os12g05440	Cytochrome P450	1530	117	2e-26	113/153	73	730	4AL12-0.43-0.59
									4DS3-0.67-0.82
									4BS8-0.57-0.81
82	m00481|LOC_Os12g05470	Plastocyanin-like domain,putative	573	126	1e-29	202/315	64	3919	2BL6-0.89-1.00
									2DL9-0.76-1.00

									2BL6-0.89-1.00
									2AL
									2AL
									2DL9-0.76-1.00
									2BL6-0.89-1.00
									2BL6-0.89-1.00
									2DL9-0.76-1.00
83	m00485|LOC_Os12g05510	hypothetical protein	1173	253	2e-67	207/255	81	8465	C-5BL14-0.75*
									C-5AL10-0.57*
									7AL18-0.90-1.00
84	m00488|LOC_Os12g05540	purple acidphosphatase-like protein	1332	662	0.0	632/832	75	8465	C-5BL14-0.75*
									C-5AL10-0.57*
									7AL18-0.90-1.00
85	m00494|LOC_Os12g05600	hydrolase, alpha/beta foldfamily, putative	1203	237	1e-62	556/958	58	13099	2DL3-0.49-0.76
									2BL2-0.36-0.50
									1BL1-0.47-0.69
86	m00499|LOC_Os12g05650	ACT domain, putative	1315	275	4e-74	397/616	64	18106	7DL2-0.61-0.82
									C-7AL1-0.39
87	m00504|LOC_Os12g05680	bZIP transcription factor	2234	269	6e-72	516/843	61	17777	C-4AL12-0.43
									4DL9-0.31-0.56
88	m00509|LOC_Os12g05730	carbonic anhydrase,putative	846	309	2e-84	354/499	70	1034	4D
									4AL4-0.80-1.00
89	m00514|LOC_Os12g05780	hypothetical protein	1047	148	5e-36	134/175	76	10131	2BL
									2DL
									C-2AL1-0.85
90	m00517|LOC_Os12g05810	syntaxin 81 (atsyp81).[mouse-ear cress	825	958	0.0	708/807	87	16022	C-2AL1-0.85
									2DL3-0.49-0.76
									2BL2-0.36-0.50
91	m00519|LOC_Os12g05830	transmembrane protein,putative	1690	150	3e-36	138/182	75	10131	2BL
									2DL
									C-2AL1-0.85
92	m00520|LOC_Os12g05840	germin-like protein -barley	948	565	e-161	531/701	75	6852	7AL16-0.86-0.90
									7DL2-0.61-0.82
									5AL23-0.87-1.00

									5AL23-0.87-1.00
									7AL16-0.86-0.90
									7BL10-0.78-1.00
93	m00522|LOC_Os12g05860	oxalate oxidase-likeprotein or germin-like protein	690	559	e-160	523/689	75	6852	7AL16-0.86-0.90
									7DL2-0.61-0.82
									5AL23-0.87-1.00
									5AL23-0.87-1.00
									7AL16-0.86-0.90
									7BL10-0.78-1.00
94	m00523|LOC_Os12g05870	oxalate oxidase-likeprotein or germin-like protein	690	530	e-151	514/689	74	6852	7AL16-0.86-0.90
									7DL2-0.61-0.82
									5AL23-0.87-1.00
									5AL23-0.87-1.00
									7AL16-0.86-0.90
									7BL10-0.78-1.00
95	m00524|LOC_Os12g05880	oxalate oxidase-likeprotein or germin-like protein	648	461	e-130	431/567	76	6852	7AL16-0.86-0.90
									7DL2-0.61-0.82
									5AL23-0.87-1.00
									5AL23-0.87-1.00
									7AL16-0.86-0.90
									7BL10-0.78-1.00
96	m00526|LOC_Os12g05900	rac GTPase activatingprotein 1	1782	1066	0.0	796/910	87	15039	5BL1-0.55-0.75
97	m00541|LOC_Os12g06050	hypothetical protein	354	141	4e-34	137/186	73	6579	4DL13-0.56-1.00*
98	m00544|LOC_Os12g06080	DNA-binding protein RAV2,putative	822	738	0.0	612/729	83	8636	C-2AL1-0.85
									2DL9-0.76-1.00
									C-5DL1-0.60
									5BL6-0.29-0.55
									C-5AL12-0.35
									2BL4-0.50-0.89
									2BL4-0.50-0.89
99	m00546|LOC_Os12g06100	expressed protein	1686	1412	0.0	1152/1409	81	16224	5AL

100	m00556|LOC_Os12g06200	E2F homolog	2074	136	6e-32	269/423	63	5147	2DL3-0.49-0.76
									C-2AL1-0.85
									2DL3-0.49-0.76
									C-2AL1-0.85
101	m00557|LOC_Os12g06200	E2F homolog	2197	136	7e-32	269/423	63	5147	2DL3-0.49-0.76
									C-2AL1-0.85
									2DL3-0.49-0.76
									C-2AL1-0.85
102	m00578|LOC_Os12g06380	transposon protein,putative, unclassified	3158	803	0.0	629/747	84	5038	5AS3-0.75-0.98
103	m00588|LOC_Os12g06490	Protein kinase domain,putative	1676	998	0.0	890/1141	78	13056	3BS1-0.33-0.57
									C-6BS5-0.76
									5DL1-0.60-0.74
									C-5BL14-0.75*
									5AL17-0.78-0.87
									C-6AS1-0.35
104	m00590|LOC_Os12g06510	EF hand, putative	828	489	e-138	455/592	76	16712	5BS8-0.56-0.71
									C-5DS1-0.63
									C-5AS1-0.40
105	m00601|LOC_Os12g06620	At3g58180/F9D24_90	1552	1038	0.0	791/928	85	16101	4DL9-0.31-0.56
									6BL5-0.40-1.00
									6DL10-0.80-1.00
									6DL10-0.80-1.00
									6DL10-0.80-1.00
106	m00603|LOC_Os12g06640	expressed protein	1827	692	0.0	559/672	83	3898	C-5AL10-0.57*
									C-5BL14-0.75*
									C-5DL1-0.60
107	m00604|LOC_Os12g06640	expressed protein	1826	692	0.0	559/672	83	3898	C-5AL10-0.57*
									C-5BL14-0.75*
									C-5DL1-0.60
108	m00606|LOC_Os12g06660	Similar to actin 3	1463	813	0.0	653/789	82	17177	5AL12-0.35-0.57
									C-5BL6-0.29
									C-5DL1-0.60
109	m00626|LOC_Os12g06840	leucine zipper protein,putative	2415	687	0.0	632/806	78	697	4DS1-0.53-0.67
									4AL12-0.43-0.59

									4BS8-0.57-0.81
110	m00632|LOC_Os12g06870	protein F14N23.29[imported] - Arabidopsis thaliana	3154	1054	0.0	900/1116	80	8329	3AS2-0.23-0.45
									3AS2-0.23-0.45
									3BS1-0.33-0.57
									3DS3-0.24-0.55
111	m00634|LOC_Os12g06890	Similar to proteinT30E16.23 [imported] - Arabidopsis thaliana	2631	1011	0.0	887/1117	79	8329	3AS2-0.23-0.45
									3AS2-0.23-0.45
									3BS1-0.33-0.57
									3DS3-0.24-0.55
112	m00643|LOC_Os12g06970	hypothetical protein	420	136	1e-32	165/235	70	9260	5AL10-0.57-0.78
									C-7BL2-0.33
									5DL5-0.76-1.00
									5BL16-0.79-1.00
113	m00647|LOC_Os12g07010	ribosomal protein L3, C427putative	1170	1469	0.0	1051/1171	89	18673	5BL6-0.29-0.55
114	m00657|LOC_Os12g07110	probable acyl-CoAsynthetase	3354	404	e-112	410/566	72	5754	C-5BL14-0.75*
									5A
115	m00658|LOC_Os12g07110	probable acyl-CoAsynthetase	2561	404	e-112	410/566	72	5754	C-5BL14-0.75*
									5A
116	m00659|LOC_Os12g07120	expressed protein	1609	103	5e-22	119/169	70	9283	2DL3-0.49-0.76
									6BL5-0.40-1.00
									3D
									6D
117	m00661|LOC_Os12g07140	V-type ATPase, C subunit	1077	778	0.0	660/785	84	15895	C-5DL1-0.60
									C-5AS1-0.40
									C-4BS4-0.37
118	m00662|LOC_Os12g07140	V-type ATPase, C subunit	1121	489	e-138	356/399	89	17669	4DS1-0.53-0.67
									4AL12-0.43-0.59
									C-5BL6-0.29
									C-5AS1-0.40
									4BS8-0.57-0.81
									4BS8-0.57-0.81
119	m00669|LOC_Os12g07210	Fructose-bisphosphatealdolase class-I	852	364	e-101	375/491	76	16079	C-5BS4-0.43

									C-5AS1-0.40
120	m00670|LOC_Os12g07220	expressed protein	1281	1069	0.0	869/1024	84	18198	C-2AS5-0.78
									2DS5-0.47-1.00
									2DS5-0.47-1.00
121	m00671|LOC_Os12g07230	Protein kinase domain,putative	1752	938	0.0	936/1281	73	16833	2DL9-0.76-1.00
									C-2AL1-0.85
122	m00705|LOC_Os12g07540	growth regulator, putative	1981	505	e-143	376/434	86	11653	2BL2-0.36-0.50
123	m00709|LOC_Os12g07580	F12P19.3 [imported] -Arabidopsis thaliana	825	100	2e-21	166/265	62	14799	C-4BL1-0.71
									4DL13-0.56-0.71
124	m00710|LOC_Os12g07590	protein tyrosinephosphatase	1453	589	e-168	486/591	82	10914	C-2AS5-0.78
									C-2AS5-0.78
									C-2BS1-0.53
									C-2DS1-0.33
									C-2BS1-0.53
125	m00711|LOC_Os12g07590	protein tyrosinephosphatase	1326	589	e-168	486/591	82	10914	C-2AS5-0.78
									C-2AS5-0.78
									C-2BS1-0.53
									C-2DS1-0.33
									C-2BS1-0.53
126	m00716|LOC_Os12g07640	typical P-type R2R3 Mybprotein	1077	307	1e-83	268/343	78	3976	6BL
									6BL
									C-6DL6-0.29
									C-6AL4-0.55
127	m00719|LOC_Os12g07670	endomembrane protein 70,putative	2381	1145	0.0	833/939	88	13728	5B
128	m00721|LOC_Os12g07690	Similar to chalconesynthase	426	137	4e-33	216/331	65	8848	1BS.sat19-0.31-0.50
									1AS3-0.86-1.00
129	m00724|LOC_Os12g07710	expressed protein	616	160	1e-39	124/143	86	14346	C-5AL10-0.57*
									C-5AL10-0.57*
130	m00726|LOC_Os12g07730	Similar to F8K7.13 protein- Arabidopsis thaliana	2696	100	8e-21	138/209	66	16938	3BS8-0.78-1.00
									3AS4-0.45-1.00
									3AS4-0.45-1.00
									3DS6-0.55-1.00

									3BS8-0.78-1.00
131	m00727|LOC_Os12g07740	translation initiationfactor eIF-2 gamma chain F20D22.6	1899	1775	0.0	1344/1549	86	18249	1DL2-0.41-1.00
132	m00731|LOC_Os12g07780	nucellin-like asparticprotease	1896	240	2e-63	265/364	72	12995	4AL12-0.43-0.59
									C-4BS4-0.37
133	m00732|LOC_Os12g07780	nucellin-like asparticprotease	1818	236	5e-62	246/334	73	12995	4AL12-0.43-0.59
									C-4BS4-0.37
134	m00734|LOC_Os12g07800	D-mannose binding lectin,putative	2343	104	2e-22	329/583	56	2160	C-7DL5-0.30
									7AL1-0.39-0.71
									7AL1-0.39-0.71
									C-7DL5-0.30
135	m00735|LOC_Os12g07810	aldehyde dehydrogenase,putative	1692	1033	0.0	1038/1425	72	18389	1AL3-0.61-1.00
									1DL2-0.41-1.00
									1BL1-0.47-0.69
									1BL1-0.47-0.69
									1DL2-0.41-1.00
136	m00736|LOC_Os12g07820	stromal ascorbateperoxidase	1302	616	e-176	504/600	84	10825	4DL9-0.31-0.56
									C-4BL1-0.71
									4AS3-0.76-1.00
137	m00737|LOC_Os12g07830	stromal ascorbateperoxidase	1326	542	e-154	487/613	79	10825	4DL9-0.31-0.56
									C-4BL1-0.71
									4AS3-0.76-1.00
138	m00738|LOC_Os12g07840	Putative methyltransferase	2973	321	1e-87	303/404	75	7274	7AL1-0.39-0.71
									7BL2-0.33-0.63
									7DL5-0.30-0.61
139	m00740|LOC_Os12g07860	MtN3/saliva family,putative	723	337	5e-93	333/449	74	12357	7AL18-0.90-1.00
									7DS4-0.61-1.00
									7BL10-0.78-1.00
									2BS3-0.84-1.00
									7BL10-0.78-1.00
									7AL16-0.86-0.90
140	m00741|LOC_Os12g07870	Similar toAT4g00750/F15P23_1	585	237	5e-63	196/243	80	7274	7AL1-0.39-0.71
									7BL2-0.33-0.63
									7DL5-0.30-0.61

141	m00753|LOC_Os12g07980	Similar to dna-directed rnapolymerase ii 8.2 kda polypeptide (ec 2.7.7.6)		131	5e-31	89/96	92	15837	3AL3-0.42-0.78
									3DL2-0.27-0.81
									3BL10-0.50-0.63
142	m00764|LOC_Os12g08090	Transmembrane amino acidtransporter protein	1930	206	6e-53	184/239	76	7914	C-2DL3-0.49
									C-2AL1-0.85
143	m00768|LOC_Os12g08130	Transmembrane amino acidtransporter protein	1797	212	7e-55	186/239	77	7914	C-2DL3-0.49
									C-2AL1-0.85
144	m00772|LOC_Os12g08170	Similar to dihydrolipoamideS-acetyltransferase, putative	1495	217	2e-56	242/343	70	7357	7BL
									5DL
									5BL16-0.79-1.00
									3AL
									3BL
145	m00781|LOC_Os12g08260	branched-chain alphaketo-acid dehydrogenase E1 alpha subunit-like protein	2168	1250	0.0	950/1112	85	17354	C-5DL1-0.60
									4BS8-0.57-0.81
									C-5BL6-0.29
146	m00782|LOC_Os12g08260	branched-chain alphaketo-acid dehydrogenase E1 alpha subunit-like protein	1807	1413	0.0	1089/1287	84	17354	C-5DL1-0.60
									4BS8-0.57-0.81
									C-5BL6-0.29
147	m00783|LOC_Os12g08260	branched-chain alphaketo-acid dehydrogenase E1 alpha subunit-like protein	1890	1393	0.0	1093/1293	84	17354	C-5DL1-0.60
									4BS8-0.57-0.81
									C-5BL6-0.29
148	m00784|LOC_Os12g08260	branched-chain alphaketo-acid dehydrogenase E1 alpha subunit-like protein	1853	1413	0.0	1089/1287	84	17354	C-5DL1-0.60
									4BS8-0.57-0.81
									C-5BL6-0.29
149	m00785|LOC_Os12g08270	3'(2'),5'-bisphosphatenucleotidase	1083	885	0.0	802/1041	77	17228	4AL5-0.66-0.80

150	m00786|LOC_Os12g08280	3'(2'),5'-bisphosphatenucleotidase	1369	1134	0.0	906/1087	83	17228	4AL5-0.66-0.80
151	m00841|LOC_Os12g08810	expressed protein	2086	794	0.0	626/747	83	16436	C-4AS1-0.20
									4BS1-0.81-1.00
									4DS2-0.82-1.00
152	m00854|LOC_Os12g08920	peroxidase, putative	1132	117	2e-26	312/541	57	16087	3AS2-0.23-0.45
									C-3BS1-0.33
									3D
									3AS2-0.23-0.45
									3AS2-0.23-0.45
									C-3BS1-0.33
153	m00868|LOC_Os12g09060	Similar to Containssimilarity to trehalose-6-phosphate phosphatase gb|AF007779.		107	9e-24	145/223	65	15960	C-5AS1-0.40
154	m00893|LOC_Os12g09300	Transmembrane amino acidtransporter protein	1899	145	8e-35	164/237	69	7914	C-2DL3-0.49
									C-2AL1-0.85
155	m00894|LOC_Os12g09300	Transmembrane amino acidtransporter protein	1817	145	8e-35	164/237	69	7914	C-2DL3-0.49
									C-2AL1-0.85
156	m00896|LOC_Os12g09320	amino acid transportprotein AAP1 - castor bean	1407	190	3e-48	179/239	74	7914	C-2DL3-0.49
									C-2AL1-0.85
157	m00903|LOC_Os12g09390	hypothetical protein	1002	100	3e-21	164/256	64	11402	4DL9-0.31-0.56
									C-4BL1-0.71
									4AS3-0.76-1.00
158	m00918|LOC_Os12g09540	phosphoribosylamine--glycine ligase	1920	903	0.0	746/923	80	5427	4AL5-0.66-0.80
									4DL13-0.56-1.00*
									7BL10-0.78-1.00
									4BL5-0.86-1.00
159	m00922|LOC_Os12g09580	Emr1	1830	684	0.0	588/740	79	8441	C-5BL14-0.75*
									C-5AS1-0.40
									C-5DL1-0.60
160	m00923|LOC_Os12g09590	Emr1	2571	567	e-161	551/735	74	8441	C-5BL14-0.75*
									C-5AS1-0.40
									C-5DL1-0.60

161	m00934|LOC_Os12g09700	jacalin homolog - barley	1236	511	e-145	625/913	68	14109	2BS3-0.84-1.00
162	m00936|LOC_Os12g09720	jakalin homolog - barley	777	282	3e-76	322/462	69	14109	2BS3-0.84-1.00
163	m00943|LOC_Os12g09790	Cytochrome P450	1467	142	5e-34	315/531	59	16621	1DL4-0.18-1.00*
									1BL1-0.47-0.69
164	m00978|LOC_Os12g10140	O-methyltransferase,putative	753	174	8e-44	395/666	59	16810	7DL5-0.30-0.61
									3AL5-0.78-1.00
									7BL2-0.33-0.63
									3DL3-0.81-1.00
165	m00983|LOC_Os12g10190	transposon protein,putative, unclassified	4233	123	8e-28	217/347	62	17291	C-2AL1-0.85
166	m01018|LOC_Os12g10540	transcription factor	858	221	4e-58	160/181	88	17287	
									1AS1-0.47-0.86
167	m01020|LOC_Os12g10560	clathrin assembly proteinAP17-like protein	906	103	3e-22	205/346	59	12336	6BS5-0.76-1.05
									6AS1-0.35-0.65
168	m01021|LOC_Os12g10570	ATP synthase F1, betasubunit	1233	469	e-132	636/972	65	17316	3BL2-0.22-0.50
									3AL3-0.42-0.78
169	m01029|LOC_Os12g10650	expressed protein	846	653	0.0	537/653	82	16548	5AL
									5BL
									C-5DL1-0.60
170	m01036|LOC_Os12g10720	Glutathione S-transferase,N-terminal domain, putative	611	510	e-145	394/467	84	12736	7BL10-0.78-1.00
									7DL2-0.61-0.82
									7AL16-0.86-0.90
									7DL2-0.61-0.82
									7AL16-0.86-0.90
171	m01037|LOC_Os12g10720	Glutathione S-transferase,N-terminal domain, putative	1276	656	0.0	517/621	83	12736	7BL10-0.78-1.00
									7DL2-0.61-0.82
									7AL16-0.86-0.90
									7DL2-0.61-0.82
									7AL16-0.86-0.90
172	m01038|LOC_Os12g10730	maleylacetoacetateisomerase	1059	510	e-145	474/622	76	13578	5AL12-0.35-0.57
173	m01039|LOC_Os12g10740	expressed protein	3328	145	1e-34	379/652	58	16028	1BL2-0.69-1.00*

									4AL12-0.43-0.59
									4DS1-0.53-0.67
									C-6AL4-0.55
									1AL3-0.61-1.00
									1AL3-0.61-1.00
									1DL
									1BL2-0.69-1.00*
174	m01043|LOC_Os12g10780	DAHP synthetase I family,putative	1470	553	e-157	437/521	83	15482	2AS5-0.78-1.00
									2DS5-0.47-1.00
									2AS5-0.78-1.00
									2AS5-0.78-1.00
									2DS5-0.47-1.00
									2BS1-0.53-0.75
									2DS5-0.47-1.00
									2BS1-0.53-0.75
175	m01044|LOC_Os12g10790	Similar to2-dehydro-3-deoxyphosphooctonate aldolase (ec 2.5.1.55)		370	e-103	270/302	89	15482	2AS5-0.78-1.00
									2DS5-0.47-1.00
									2AS5-0.78-1.00
									2AS5-0.78-1.00
									2DS5-0.47-1.00
									2BS1-0.53-0.75
									2DS5-0.47-1.00
									2BS1-0.53-0.75
176	m01177|LOC_Os12g12110	hypothetical protein	351	137	3e-33	111/136	81	16501	C-5DL1-0.60
									5AL
177	m01183|LOC_Os12g12170	Cytochrome b5-likeHeme/Steroid binding domain, putative	927	470	e-133	409/517	79	8065	7DL2-0.61-0.82
									7AL21-0.74-0.86
									7BL7-0.63-0.78
178	m01184|LOC_Os12g12170	Cytochrome b5-likeHeme/Steroid binding domain, putative	825	470	e-133	409/517	79	8065	7DL2-0.61-0.82
									7AL21-0.74-0.86
									7BL7-0.63-0.78

179	m01204|LOC_Os12g12360	Similar toAt2g43970/F6E13.10	1698	129	4e-30	326/557	58	15718	3BL2-0.22-0.50
									3DL2-0.27-0.81
									3AL3-0.42-0.78
180	m01215|LOC_Os12g12470	oxidoreductase,zinc-binding dehydrogenase family	1245	396	e-110	379/504	75	9646	2BS3-0.84-1.00
									5AL12-0.35-0.57
									2BS3-0.84-1.00
									5BL1-0.55-0.75
									5BL1-0.55-0.75
									5DL1-0.60-0.74
181	m01216|LOC_Os12g12470	oxidoreductase,zinc-binding dehydrogenase family	1098	448	e-126	421/555	75	9646	2BS3-0.84-1.00
									5AL12-0.35-0.57
									2BS3-0.84-1.00
									5BL1-0.55-0.75
									5BL1-0.55-0.75
									5DL1-0.60-0.74
182	m01220|LOC_Os12g12510	hypothetical protein	561	258	3e-69	292/412	70	9646	2BS3-0.84-1.00
									5AL12-0.35-0.57
									2BS3-0.84-1.00
									5BL1-0.55-0.75
									5BL1-0.55-0.75
									5DL1-0.60-0.74
183	m01221|LOC_Os12g12520	At1g65560/F5I14_32	600	179	2e-45	204/286	71	12544	4BS8-0.57-0.81
									4AL12-0.43-0.59
									4AL12-0.43-0.59
									4AL12-0.43-0.59
184	m01225|LOC_Os12g12560	allyl alcohol dehydrogenase	1246	427	e-120	411/548	75	9646	2BS3-0.84-1.00
									5AL12-0.35-0.57
									2BS3-0.84-1.00
									5BL1-0.55-0.75
									5BL1-0.55-0.75
									5DL1-0.60-0.74
185	m01227|LOC_Os12g12580	oxidoreductase,zinc-binding dehydrogenase family	1324	435	e-122	668/1042	64	11439	3DL2-0.27-0.81

									3AL5-0.78-1.00
									3BL7-0.63-1.00
									3DL2-0.27-0.81
									3DL2-0.27-0.81
									3AL5-0.78-1.00
									3DL2-0.27-0.81
186	m01228|LOC_Os12g12590	oxidoreductase,zinc-binding dehydrogenase family	1041	467	e-132	426/553	77	9646	2BS3-0.84-1.00
									5AL12-0.35-0.57
									2BS3-0.84-1.00
									5BL1-0.55-0.75
									5BL1-0.55-0.75
									5DL1-0.60-0.74
187	m01229|LOC_Os12g12600	Similar tojasmonate-induced protein homolog - wheat (fragment)	465	221	2e-58	295/451	65	14109	2BS3-0.84-1.00
188	m01234|LOC_Os12g12650	hypothetical protein	1098	104	1e-22	225/365	61	15676	7BL7-0.63-0.78
									7AL21-0.74-0.86
189	m01241|LOC_Os12g12720	Similar to jacalin homolog- barley	783	309	2e-84	348/492	70	14109	2BS3-0.84-1.00
190	m01242|LOC_Os12g12730	probable calcium bindingprotein [imported] - Arabidopsis thaliana	661	152	3e-37	182/249	73	16191	3AL5-0.78-1.00
191	m01254|LOC_Os12g12850	Clp amino terminal domain,putative	4224	1370	0.0	970/1076	90	13724	1DL2-0.41-1.00
									1AL1-0.17-0.61
									2BL6-0.89-1.00
									2DL9-0.76-1.00
192	m01255|LOC_Os12g12860	calcium-dependent proteinkinase (EC 2.7.1.-) 1 - Arabidopsis thaliana	2385	437	e-122	693/1101	62	16833	2DL9-0.76-1.00
									C-2AL1-0.85
193	m01263|LOC_Os12g12940	POT family	1835	193	4e-49	359/582	61	4479	5BS8-0.56-0.71
									C-5DS1-0.63
									2BL2-0.36-0.50
194	m01268|LOC_Os12g12990	transposon protein,putative, mariner sub-class	3180	110	4e-24	120/171	70	4423	C-6AS1-0.35
									3BS8-0.78-1.00
									3BS8-0.78-1.00
195	m01272|LOC_Os12g13030	Phosphatidylethanolamine-binding protein	537	150	8e-37	317/525	60	11442	7AS8-0.45-0.59

									7DS5-0.36-0.61
196	m01302|LOC_Os12g13320	argininosuccinate synthase	1780	1577	0.0	1121/1248	89	17492	1DL2-0.41-1.00
197	m01308|LOC_Os12g13380	adenylate kinase a (ec2.7.4.3) (atp-amp transphosphorylase)	1156	952	0.0	781/922	84	18143	5BS4-0.43-0.56
									C-5DS1-0.63
									C-5AL12-0.35
									C-5AL12-0.35
198	m01309|LOC_Os12g13390	aspartylaminopeptidase-like protein	1737	855	0.0	632/720	87	13708	C-5AL17-0.78*
									C-5AL17-0.78*
									C-5BL14-0.75*
199	m01316|LOC_Os12g13460	SET domain, putative	1532	1437	0.0	1058/1210	87	15230	5BS4-0.43-0.56
									C-5AS1-0.40
200	m01329|LOC_Os12g13590	exocyst complex componentsec10	2875	860	0.0	631/720	87	10320	5AS1-0.40-0.75
									C-5DS1-0.63
									5BS4-0.43-0.56
201	m01334|LOC_Os12g13640	Similar to HGA6	1945	258	1e-68	446/710	62	11933	1BL1-0.47-0.69
									1AL3-0.61-1.00
202	m01349|LOC_Os12g13790	Similar toAt1g62200/F19K23_13	1401	104	1e-22	134/203	66	16668	1DL2-0.41-1.00
									1AL1-0.17-0.61
									1BL1-0.47-0.69
203	m01350|LOC_Os12g13800	O-methyltransferase	789	293	1e-79	393/588	66	16810	7DL5-0.30-0.61
									3AL5-0.78-1.00
									7BL2-0.33-0.63
									3DL3-0.81-1.00
204	m01351|LOC_Os12g13810	Similar to caffeic acidO-methyltransferase	462	174	5e-44	258/402	64	16810	7DL5-0.30-0.61
									3AL5-0.78-1.00
									7BL2-0.33-0.63
									3DL3-0.81-1.00
205	m01364|LOC_Os12g13930	transposon protein,putative, CACTA, En/Spm sub-class	1503	472	e-133	358/414	86	8166	5AL17-0.78-0.87
									5DL5-0.76-1.00
									5BL16-0.79-1.00
206	m01378|LOC_Os12g14070	chaperone protein DnaK	2575	901	0.0	796/1024	77	17374	2DL3-0.49-0.76
									2BL2-0.36-0.50

207	m01381|LOC_Os12g14100	amino acid transporter-likeprotein 2	1341	537	e-153	451/554	81	12299	2AL1-0.85-1.00
									2BL4-0.50-0.89
									2DL9-0.76-1.00
208	m01382|LOC_Os12g14110	hypothetical protein	630	385	e-107	365/473	77	12262	2AS
									2BL
209	m01415|LOC_Os12g14440	jacalin homolog - barley	1205	535	e-152	643/934	68	14109	2BS3-0.84-1.00
210	m01429|LOC_Os12g14580	Tetraspanin family	933	144	1e-34	154/213	72	13547	
									1DS5-0.70-1.00
									1AS1-0.47-0.86
211	m01518|LOC_Os12g15460	expressed protein	3793	177	5e-44	219/302	72	16142	1A
212	m01522|LOC_Os12g15490	hypothetical protein	258	128	2e-30	132/169	78	16501	C-5DL1-0.60
									5AL
213	m01526|LOC_Os12g15530	retrotransposon protein,putative, Ty1-copia sub-class	2715	263	6e-70	365/555	65	13628	C-3BL10-0.50*
									3A
									2BS
									3DL2-0.27-0.81
214	m01542|LOC_Os12g15680	Multicopper oxidase,putative	1945	286	3e-77	389/583	66	13628	C-3BL10-0.50*
									3A
									2BS
									3DL2-0.27-0.81
215	m01566|LOC_Os12g15920	Multicopper oxidase,putative	1977	274	2e-73	426/665	64	13628	C-3BL10-0.50*
									3A
									2BS
									3DL2-0.27-0.81
216	m01597|LOC_Os12g16220	isoflavone reductasehomolog (ec 1.3.1.-)	1199	733	0.0	696/925	75	14654	7BS1-0.27-1.00
									7BS1-0.27-1.00
									7DS5-0.36-0.61
									7AS8-0.45-0.59
									7DS5-0.36-0.61
217	m01598|LOC_Os12g16220	isoflavone reductasehomolog (ec 1.3.1.-)	1202	721	0.0	696/928	75	14654	7BS1-0.27-1.00
									7BS1-0.27-1.00
									7DS5-0.36-0.61
									7AS8-0.45-0.59

									7DS5-0.36-0.61
218	m01600|LOC_Os12g16240	Similar topinoresinol-lariciresinol reductase TH1	737	396	e-110	387/525	73	14654	7BS1-0.27-1.00
									7BS1-0.27-1.00
									7DS5-0.36-0.61
									7AS8-0.45-0.59
									7DS5-0.36-0.61
219	m01601|LOC_Os12g16250	Similar to isoflavonereductase homolog (ec 1.3.1.-)	384	240	4e-64	236/316	74	14654	7BS1-0.27-1.00
									7BS1-0.27-1.00
									7DS5-0.36-0.61
									7AS8-0.45-0.59
									7DS5-0.36-0.61
220	m01605|LOC_Os12g16290	Similar to isoflavonereductase homolog (ec 1.3.1.-)	2892	250	4e-66	239/316	75	14654	7BS1-0.27-1.00
									7BS1-0.27-1.00
									7DS5-0.36-0.61
									7AS8-0.45-0.59
									7DS5-0.36-0.61
221	m01606|LOC_Os12g16300	hypothetical protein	273	164	2e-41	119/135	88	12920	C-2DL3-0.49
									C-2AL1-0.85
									2BL2-0.36-0.50
222	m01611|LOC_Os12g16350	3-hydroxyisobutyryl-coenzyme A hydrolase, putative	2350	141	3e-33	108/128	84	18220	C-7DL5-0.30
223	m01612|LOC_Os12g16350	3-hydroxyisobutyryl-coenzyme A hydrolase, putative	3156	141	4e-33	108/128	84	18220	C-7DL5-0.30
224	m01619|LOC_Os12g16410	isoflavone reductasehomolog (ec 1.3.1.-)	1222	721	0.0	692/925	74	14654	7BS1-0.27-1.00
									7BS1-0.27-1.00
									7DS5-0.36-0.61
									7AS8-0.45-0.59
									7DS5-0.36-0.61
225	m01643|LOC_Os12g16650	Similar to gene_id:MEE5.5	1908	402	e-112	334/410	81	9180	7DS4-0.61-1.00
									4AL5-0.66-0.80
									7AS5-0.59-0.89
									3AL5-0.78-1.00

226	m01650|LOC_Os12g16720	cytochrome P-450LXXIA1[similarity] - avocado	1904	180	3e-45	238/353	67	4438	1A
									C-1DL4-0.18
									C-1BL6-0.32
227	m01686|LOC_Os12g17080	expressed protein	2385	367	e-101	277/323	85	11566	4DS2-0.82-1.00
									4BS4-0.37-0.57
									4AL13-0.59-0.66
228	m01687|LOC_Os12g17090	NB-ARC domain, putative	3471	125	2e-28	266/435	61	4054	6DL10-0.80-1.00
									6BL5-0.40-1.00
									6AL8-0.90-1.00
229	m01710|LOC_Os12g17320	Polyprenyl synthetase,putative	1856	854	0.0	721/904	79	14281	4DL9-0.31-0.56
									4BL5-0.86-1.00
									4AS4-0.63-1.00*
230	m01727|LOC_Os12g17490	NB-ARC domain, putative	2646	120	5e-27	166/252	65	4054	6DL10-0.80-1.00
									6BL5-0.40-1.00
									6AL8-0.90-1.00
231	m01732|LOC_Os12g17540	Papain family cysteineprotease, putative	1053	345	3e-95	589/926	63	15988	C-5DL1-0.60
									C-5AL10-0.57*
232	m01735|LOC_Os12g17570	GDSL-likeLipase/Acylhydrolase	1221	301	9e-82	453/682	66	16465	1DL2-0.41-1.00
									1AL3-0.61-1.00
									1BL1-0.47-0.69
									1BL1-0.47-0.69
233	m01738|LOC_Os12g17600	Ribulose bisphosphatecarboxylase, small subunit, putative	1083	594	e-170	445/516	86	18798	C-5AL10-0.57*
									C-5DL1-0.60
									C-5BL14-0.75*
234	m01770|LOC_Os12g17910	rubisco subunitbinding-protein alpha subunit, chloroplast precursor		2043	0.0	1541/1789	86	18601	2BS3-0.84-1.00
									2AS5-0.78-1.00
									2DS5-0.47-1.00
235	m01788|LOC_Os12g18090	hypothetical protein	1179	114	2e-25	121/171	70	4423	C-6AS1-0.35
									3BS8-0.78-1.00
									3BS8-0.78-1.00
236	m01804|LOC_Os12g18220	Similar to thioredoxin hisoform 1	633	120	1e-27	144/208	69	17318	C-2BS1-0.53
									C-2AL1-0.85

									C-2AL1-0.85
									C-2DL3-0.49
237	m01894|LOC_Os12g19030	Copine, putative	729	175	3e-44	263/416	63	4116	7A
									C-7BL2-0.33
									7D
238	m01897|LOC_Os12g19060	protein F3F19.5 [imported]- Arabidopsis thaliana	1302	107	2e-23	88/109	80	18165	6BL5-0.40-1.00
									6AL4-0.55-0.90
									6DL6-0.29-0.47
239	m01900|LOC_Os12g19090	expressed protein	964	470	e-133	458/605	75	16439	2AL
									2AL
240	m01921|LOC_Os12g19300	expressed protein	1679	740	0.0	558/645	86	507	C-5AS1-0.40
									5B
									C-5DS1-0.63
									C-5DS1-0.63
									C-5AS1-0.40
									C-5BS8-0.56*
									C-5DS1-0.63
241	m01922|LOC_Os12g19300	expressed protein	2315	756	0.0	568/655	86	507	C-5AS1-0.40
									5B
									C-5DS1-0.63
									C-5DS1-0.63
									C-5AS1-0.40
									C-5BS8-0.56*
									C-5DS1-0.63
242	m01930|LOC_Os12g19380	Ribulose bisphosphatecarboxylase, small subunit	1066	575	e-164	439/516	85	18798	C-5AL10-0.57*
									C-5DL1-0.60
									C-5BL14-0.75*
243	m01931|LOC_Os12g19380	Ribulose bisphosphatecarboxylase, small subunit	1011	462	e-130	363/435	83	18798	C-5AL10-0.57*
									C-5DL1-0.60
									C-5BL14-0.75*
244	m01932|LOC_Os12g19390	Ribulose bisphosphatecarboxylase, small subunit, putative	372	194	2e-50	154/186	82	18798	C-5AL10-0.57*

									C-5DL1-0.60
									C-5BL14-0.75*
245	m01934|LOC_Os12g19410	Ribulose bisphosphatecarboxylase, small subunit, putative	1094	595	e-170	446/517	86	18798	C-5AL10-0.57*
									C-5DL1-0.60
									C-5BL14-0.75*
246	m01940|LOC_Os12g19470	Ribulose bisphosphatecarboxylase, small subunit, putative	2048	519	e-147	422/517	81	18798	C-5AL10-0.57*
									C-5DL1-0.60
									C-5BL14-0.75*
247	m01941|LOC_Os12g19470	Ribulose bisphosphatecarboxylase, small subunit, putative	1962	519	e-147	422/517	81	18798	C-5AL10-0.57*
									C-5DL1-0.60
									C-5BL14-0.75*
248	m01942|LOC_Os12g19470	Ribulose bisphosphatecarboxylase, small subunit, putative	2151	364	e-100	302/375	80	18163	C-5DS1-0.63
									C-2AS5-0.78
									2BS3-0.84-1.00
									2DS5-0.47-1.00
									C-2AS5-0.78
									2BS3-0.84-1.00
									2BS3-0.84-1.00
									C-2AS5-0.78
									2BS3-0.84-1.00
249	m02028|LOC_Os12g20320	Cyclin, N-terminal domain,putative	909	129	2e-30	229/372	61	16528	C-4AL12-0.43
									C-4BL1-0.71
									C-4DL9-0.31
									2BS1-0.53-0.75
									1BL1-0.47-0.69
									C-4BL1-0.71
250	m02078|LOC_Os12g21800	Ribosomal S3Ae family,putative	2403	744	0.0	606/743	81	18766	4BL5-0.86-1.00
									4DL13-0.56-1.00*
									4AS4-0.63-0.76
251	m02101|LOC_Os12g22030	serinehydroxymethyltransferase	1275	1334	0.0	1061/1276	83	18296	

									C-2AL1-0.85
252	m02159|LOC_Os12g22600	tRNA synthetases class II(D, K and N), putative	1848	150	3e-36	218/342	63	8103	1AS1-0.47-0.86
									1BS10-0.50-0.84
									C-1DS3-0.48
253	m02164|LOC_Os12g22650	M36658 histone H3	411	412	e-115	335/411	81	14705	7AS5-0.59-0.89
254	m02167|LOC_Os12g22680	M36658 histone H3	411	434	e-122	342/411	83	14705	7AS5-0.59-0.89
255	m02215|LOC_Os12g23150	p-nitrophenylphosphatase,putative	702	511	e-145	406/475	85	16811	2DL3-0.49-0.76
									1AS1-0.47-0.86
									2BS3-0.84-1.00
									C-1BL6-0.32
									C-1BL6-0.32
									C-1BL6-0.32
									C-7DS5-0.36
									C-7DS5-0.36
256	m02217|LOC_Os12g23170	Glycosyl hydrolase family 1	1527	239	4e-63	346/542	63	12903	4AL13-0.59-0.66
									4DS3-0.67-0.82
									4DS3-0.67-0.82
									4BS1-0.81-1.00
									3DL2-0.27-0.81
									C-3AL3-0.42
									3DL2-0.27-0.81
257	m02218|LOC_Os12g23180	RNA binding protein,putative	1215	551	e-157	418/489	85	17862	5AS3-0.75-0.98
									5BS5-0.71-1.00*
									5DS1-0.63-0.67
									5BS5-0.71-1.00*
									5AS3-0.75-0.98
258	m02219|LOC_Os12g23190	Putative tyrosinephosphatase family	615	210	7e-55	304/471	64	13410	6DL11-0.74-0.80
									6AL8-0.90-1.00
									6BL5-0.40-1.00
									6BL5-0.40-1.00
259	m02220|LOC_Os12g23200	photosystem I reactioncenter subunit XI, putative	627	757	0.0	556/630	88	16529	
									2AS5-0.78-1.00

260	m02228|LOC_Os12g23280	Heavy-metal-associateddomain, putative	1351	405	e-113	354/429	82	14854	5AL23-0.87-1.00
									4BL5-0.86-1.00
									4DL13-0.56-1.00*
261	m02229|LOC_Os12g23280	Heavy-metal-associateddomain, putative	1309	400	e-112	363/440	82	14854	5AL23-0.87-1.00
									4BL5-0.86-1.00
									4DL13-0.56-1.00*
262	m02305|LOC_Os12g24040	endo-1,4-beta-D-glucanaseKORRIGAN	2085	156	4e-38	131/164	79	8388	2AL1-0.85-1.00
									2BL6-0.89-1.00
									4AL4-0.80-1.00
									2DL9-0.76-1.00
263	m02307|LOC_Os12g24060	hypothetical protein	318	156	6e-39	122/146	83	8388	2AL1-0.85-1.00
									2BL6-0.89-1.00
									4AL4-0.80-1.00
									2DL9-0.76-1.00
264	m02309|LOC_Os12g24080	retrotransposon protein,putative, unclassified	13041	1455	0.0	1089/1246	87	16190	C-5AL10-0.57*
265	m02318|LOC_Os12g24170	AR782	954	725	0.0	574/686	83	8327	5A
									5B
266	m02319|LOC_Os12g24180	Similar toAt2g32810/F24L7.5	876	190	2e-48	156/189	82	8327	5A
									5B
267	m02333|LOC_Os12g24320	ATPase, AAA family,putative	1620	139	5e-33	147/202	72	1719	4AL12-0.43-0.59
									4DS3-0.67-0.82
268	m02334|LOC_Os12g24330	hypothetical protein	2334	873	0.0	1135/1625	69	16932	4AL13-0.59-0.66
									7AS1-0.89-1.00
									7DS4-0.61-1.00
269	m02355|LOC_Os12g24540	PHD-finger, putative	4190	228	3e-59	267/371	71	2097	5AL10-0.57-0.78
									5DL1-0.60-0.74
									5BL14-0.75-0.76
270	m02359|LOC_Os12g24580	expressed protein	534	118	3e-27	98/117	83	12029	2BS1-0.53-0.75
									2AS5-0.78-1.00
271	m02366|LOC_Os12g24650	leucyl aminopeptidase (EC3.4.11.1) (clone TPP6) - tomato (fragment)	1834	538	e-153	453/562	80	14190	C-1BL6-0.32
									1AS1-0.47-0.86

									C-1DS3-0.48
272	m02381|LOC_Os12g24800	Retinal pigment epithelialmembrane protein	1834	404	e-112	443/632	70	11924	6AL4-0.55-1.00*
									6BL5-0.40-1.00
									6DL6-0.29-0.47
273	m02410|LOC_Os12g25090	retrotransposon protein,putative, Ty1-copia sub-class	1004	147	1e-35	142/192	73	16076	1AS1-0.47-0.86
									1BS.sat18-0.50-1.00
									1DS5-0.70-1.00
									1DS5-0.70-1.00
274	m02411|LOC_Os12g25090	retrotransposon protein,putative, Ty1-copia sub-class	1210	147	2e-35	142/192	73	16076	1AS1-0.47-0.86
									1BS.sat18-0.50-1.00
									1DS5-0.70-1.00
									1DS5-0.70-1.00
275	m02414|LOC_Os12g25120	Core histone H2A/H2B/H3/H4,putative	825	538	e-153	392/440	89	17661	C-2BS1-0.53
276	m02418|LOC_Os12g25160	hypothetical protein	336	145	1e-35	139/187	74	6579	4DL13-0.56-1.00*
277	m02422|LOC_Os12g25200	CLC-d chloride channel;anion channel protein	2691	104	3e-22	83/101	82	9923	6BS5-0.76-1.05
									6DS2-0.45-0.79
									6AS5-0.65-1.00
									6BS5-0.76-1.05
278	m02447|LOC_Os12g25450	o-methyltransferase zrp4(ec 2.1.1.-) (omt). [maize	1080	293	2e-79	527/845	62	13223	6DS6-0.99-1.00
279	m02451|LOC_Os12g25490	o-methyltransferase zrp4(ec 2.1.1.-) (omt). [maize	1347	318	5e-87	537/844	63	13223	6DS6-0.99-1.00
280	m02468|LOC_Os12g25660	Similar to cytochromeP450-like protein - Arabidopsis thaliana	1628	158	1e-38	318/517	61	13305	3BS9-0.57-0.78
									3BS9-0.57-0.78
									3DS6-0.55-1.00
									3BS9-0.57-0.78
									3DS6-0.55-1.00
									3AS4-0.45-1.00
281	m02470|LOC_Os12g25680	Similar to cysteineproteinase	1628	103	3e-22	125/181	69	15988	C-5DL1-0.60

									C-5AL10-0.57*
282	m02471|LOC_Os12g25690	UDP-glucose/GDP-mannosedehydrogenase family, NAD binding domain, putative	1908	1711	0.0	1293/1497	86	18378	4AL12-0.43-0.59
									5BL9-0.76-0.79
									4BS8-0.57-0.81
									4DS2-0.82-1.00
283	m02472|LOC_Os12g25700	UDP-glucose/GDP-mannosedehydrogenase family, NAD binding domain, putative	2049	1597	0.0	1236/1460	84	18378	4AL12-0.43-0.59
									5BL9-0.76-0.79
									4BS8-0.57-0.81
									4DS2-0.82-1.00
284	m02473|LOC_Os12g25700	UDP-glucose/GDP-mannosedehydrogenase family, NAD binding domain, putative	1921	1602	0.0	1248/1476	84	18378	4AL12-0.43-0.59
									5BL9-0.76-0.79
									4BS8-0.57-0.81
									4DS2-0.82-1.00
285	m02486|LOC_Os12g25820	o-methyltransferase zrp4(ec 2.1.1.-) (omt). [maize	1092	424	e-119	439/601	73	3596	1BL6-0.32-0.47
									1AL1-0.17-0.61
									1DL2-0.41-1.00
286	m02490|LOC_Os12g25860	Similar too-methyltransferase zrp4 (ec 2.1.1.-) (omt). [maize	714	293	1e-79	370/531	69	3596	1BL6-0.32-0.47
									1AL1-0.17-0.61
									1DL2-0.41-1.00
287	m02507|LOC_Os12g26030	contains similarity to theN terminal domain of the E1 protein x	714	332	7e-91	449/684	65	12956	2DL9-0.76-1.00
288	m02616|LOC_Os12g27100	retrotransposon protein,putative, Ty1-copia sub-class	6936	207	7e-53	396/632	62	10857	
									2BL2-0.36-0.50
									2BL2-0.36-0.50
									2DL3-0.49-0.76
289	m02629|LOC_Os12g27220	Transferase family	1332	212	5e-55	296/444	66	12897	7AL18-0.90-1.00
									7BL10-0.78-1.00
									7BL10-0.78-1.00
									7AL18-0.90-1.00

									7DL3-0.82-1.00
									3BS8-0.78-1.00
									3BS8-0.78-1.00
									7DL3-0.82-1.00
290	m02633|LOC_Os12g27260	hypothetical protein	1332	177	9e-45	284/442	64	12897	7AL18-0.90-1.00
									7BL10-0.78-1.00
									7BL10-0.78-1.00
									7AL18-0.90-1.00
									7DL3-0.82-1.00
									3BS8-0.78-1.00
									3BS8-0.78-1.00
									7DL3-0.82-1.00
291	m02640|LOC_Os12g27330	Transferase family	1044	180	1e-45	283/438	64	12897	7AL18-0.90-1.00
									7BL10-0.78-1.00
									7BL10-0.78-1.00
									7AL18-0.90-1.00
									7DL3-0.82-1.00
									3BS8-0.78-1.00
									3BS8-0.78-1.00
									7DL3-0.82-1.00
292	m02642|LOC_Os12g27350	Transferase family	1638	196	4e-50	290/447	64	12897	7AL18-0.90-1.00
									7BL10-0.78-1.00
									7BL10-0.78-1.00
									7AL18-0.90-1.00
									7DL3-0.82-1.00
									3BS8-0.78-1.00
									3BS8-0.78-1.00
									7DL3-0.82-1.00
293	m02659|LOC_Os12g27520	protein kinase afc2 (ec2.7.1.-)	1572	1407	0.0	990/1093	90	15755	6DL6-0.29-0.47
									C-6BL3-0.36
									C-6AL4-0.55
294	m02660|LOC_Os12g27520	protein kinase afc2 (ec2.7.1.-)	1667	1126	0.0	775/840	92	15755	6DL6-0.29-0.47
									C-6BL3-0.36
									C-6AL4-0.55

295	m02661|LOC_Os12g27520	protein kinase afc2 (ec2.7.1.-)	1658	933	0.0	633/678	93	15755	6DL6-0.29-0.47
									C-6BL3-0.36
									C-6AL4-0.55
296	m02673|LOC_Os12g27640	transposon protein,putative, CACTA, En/Spm sub-class	4101	244	4e-64	248/333	74	17813	2AL
									2DL
									C-2AL1-0.85
									C-2AL1-0.85
									1DS5-0.70-1.00
297	m02723|LOC_Os12g28140	ATPase, AAA family,putative	1986	109	8e-24	119/170	70	13819	3DS6-0.55-1.00
298	m02727|LOC_Os12g28180	ATPase, AAA family,putative	1986	109	8e-24	119/170	70	13819	3DS6-0.55-1.00
299	m02755|LOC_Os12g28460	Similar to mitochondrialprotein	1125	120	2e-27	126/177	71	13819	3DS6-0.55-1.00
300	m02768|LOC_Os12g28590	expressed protein	1829	109	7e-24	121/174	69	13819	3DS6-0.55-1.00
301	m02826|LOC_Os12g29170	hypothetical protein	360	169	1e-42	151/191	79	4871	6AS5-0.65-1.00
									6BS5-0.76-1.05
									C-6DS2-0.45
302	m02831|LOC_Os12g29220	MTN3 homolog [imported] -Arabidopsis thaliana	1677	727	0.0	664/850	78	17133	6BL5-0.40-1.00
									6BL5-0.40-1.00
303	m02846|LOC_Os12g29370	hypothetical protein	909	269	2e-72	371/553	67	6520	5DL5-0.76-1.00
									5BL
									5AL17-0.78-0.87
304	m02849|LOC_Os12g29400	Similar to ABA-responsiveprotein - barley	1124	148	5e-36	260/422	61	16419	2BL2-0.36-0.50
									2DL3-0.49-0.76
									C-2AL1-0.85
									2BL2-0.36-0.50
									2BL2-0.36-0.50
305	m02861|LOC_Os12g29520	Auxin response factor,putative	3177	307	3e-83	332/461	72	8980	3BL7-0.63-1.00
									3AL5-0.78-1.00
306	m02862|LOC_Os12g29520	Auxin response factor,putative	3323	307	3e-83	332/461	72	8980	3BL7-0.63-1.00
									3AL5-0.78-1.00
307	m02867|LOC_Os12g29570	one helix protein	580	234	4e-62	196/245	80	14236	6BL
									6AL4-0.55-0.90
									6DL

308	m02879|LOC_Os12g29690	NBS-LRR disease resistanceprotein homologue	4515	345	1e-94	583/899	64	6925	3AS4-0.45-1.00
309	m02881|LOC_Os12g29710	Similar to NBS-LRR diseaseresistance protein homologue	4575	218	2e-56	330/493	66	6925	3AS4-0.45-1.00
310	m02907|LOC_Os12g29960	Similar to yippee-likeprotein at4g27740. [mouse-ear cress]	321	324	1e-89	262/320	81	11041	5AL12-0.35-0.57
311	m02909|LOC_Os12g29980	expressed protein	1592	139	5e-33	123/159	77	11904	C-7BL2-0.33
312	m02910|LOC_Os12g29990	O-sialoglycoproteinendopeptidase, putative	1695	938	0.0	674/757	89	9875	3DS3-0.24-0.55
									3DS3-0.24-0.55
									3BS1-0.33-0.57
313	m02911|LOC_Os12g29990	O-sialoglycoproteinendopeptidase, putative	1695	930	0.0	669/752	88	9875	3DS3-0.24-0.55
									3DS3-0.24-0.55
									3BS1-0.33-0.57
314	m02918|LOC_Os12g30060	probable prefoldin subunit2	787	451	e-127	367/450	81	14886	2DL9-0.76-1.00
									7AS5-0.59-0.89
									7DS4-0.61-1.00
315	m02919|LOC_Os12g30060	probable prefoldin subunit2	1226	451	e-127	367/450	81	14886	2DL9-0.76-1.00
									7AS5-0.59-0.89
									7DS4-0.61-1.00
316	m02928|LOC_Os12g30150	calcium-dependent proteinkinase 3	1950	827	0.0	897/1273	70	16833	2DL9-0.76-1.00
									C-2AL1-0.85
317	m02931|LOC_Os12g30180	Similar to stem rustresistance protein	1611	142	6e-34	279/464	60	12999	C-4AL12-0.43
									4DS3-0.67-0.82
318	m02943|LOC_Os12g30300	Similar tocalcium-dependent protein kinase 3	1173	410	e-114	462/666	69	16833	2DL9-0.76-1.00
									C-2AL1-0.85
319	m02962|LOC_Os12g30490	ribosomal protein L4/L1family, putative	867	484	e-137	381/457	83	15105	3DL2-0.27-0.81
									3BL10-0.50-0.63
320	m02968|LOC_Os12g30550	expressed protein	1060	215	4e-56	218/301	72	11855	6A
									6D
									6BS
321	m02970|LOC_Os12g30570	Similar to ATMRK1	1155	269	3e-72	391/598	65	13501	5DL5-0.76-1.00
									5AL10-0.57-0.78
									5BL9-0.76-0.79
322	m03013|LOC_Os12g31000	Similar to F16L1.3 protein- Arabidopsis	2010	608	e-174	496/599	82	7224	C-5BS4-0.43

		thaliana							
									5AS
									C-5BS4-0.43
323	m03058|LOC_Os12g31450	12187-10624	726	608	e-174	558/723	77	11342	5AL
324	m03059|LOC_Os12g31460	Similar to DnaJ domainfamily	708	588	e-168	552/709	77	12376	C-5AL10-0.57*
									C-5BL14-0.75*
325	m03060|LOC_Os12g31470	retrotransposon protein,putative, unclassified	6801	129	2e-29	116/151	76	6898	C-7DL5-0.30
									7AL1-0.39-0.71
326	m03077|LOC_Os12g31640	vacuolar sortingprotein-like; embryogenesis protein H beta 58-like protein	768	722	0.0	599/733	81	18151	C4A
									C-4DS1-0.53
									C-4BS4-0.37
327	m03089|LOC_Os12g31760	MADS box protein ZMM4 -maize (fragment)	198	171	2e-43	142/177	80	8488	C-2DL3-0.49
									C-2AL1-0.85
328	m03091|LOC_Os12g31780	expressed protein	992	164	8e-41	209/305	68	18379	2DS5-0.47-1.00
									C-2BS1-0.53
329	m03093|LOC_Os12g31800	RNA-binding proteinprecursor	997	366	e-101	358/466	76	8990	3BL2-0.22-0.50
									3BL2-0.22-0.50
330	m03095|LOC_Os12g31820	phosphoserine phosphataseSerB, putative	1352	618	e-177	607/825	73	13554	2BS1-0.53-0.75
									C-3AS2-0.23
									7AS5-0.59-0.89
331	m03132|LOC_Os12g32130	Trehalose-phosphatase	666	153	1e-37	298/500	59	14699	4AS3-0.76-1.00
									4DL13-0.56-0.71
									4BL1-0.71-0.86
									4DL13-0.56-0.71
									4BL1-0.71-0.86
332	m03142|LOC_Os12g32230	radical SAM domain protein,putative	1795	1171	0.0	868/993	87	15888	5DS1-0.63-0.67
									5AS3-0.75-0.98
									5BS8-0.56-0.71
									5DS1-0.63-0.67
333	m03143|LOC_Os12g32230	radical SAM domain protein,putative	1790	1187	0.0	868/988	87	15888	5DS1-0.63-0.67
									5AS3-0.75-0.98
									5BS8-0.56-0.71

									5DS1-0.63-0.67
334	m03144|LOC_Os12g32240	translation initiationfactor eIF-5A	993	599	e-171	472/547	86	10419	C-5DS1-0.63
335	m03145|LOC_Os12g32250	Similar to zinc fingertranscription factor WRKY1	2091	182	9e-46	169/224	75	14864	C-2AS5-0.78
									C-2BS1-0.53
336	m03155|LOC_Os12g32360	expressed protein	937	110	1e-24	93/117	79	6192	4BS8-0.57-0.81
									4DS2-0.82-1.00
									4DS2-0.82-1.00
									4BS8-0.57-0.81
									4DS2-0.82-1.00
337	m03158|LOC_Os12g32380	ribosomal proteinS14p/S29e, putative	312	217	4e-57	149/162	91	18382	C-5AL10-0.57*
									C-5BL14-0.75*
									C-5DL1-0.60
338	m03170|LOC_Os12g32500	hypothetical protein	342	133	9e-32	107/131	81	17216	C-7BL2-0.33
									C-7AL1-0.39
									C-7DL5-0.30
									C-7AL1-0.39
									C-7AL1-0.39
									C-7DL5-0.30
339	m03182|LOC_Os12g32620	pollen specific LIM domainprotein 1b	854	738	0.0	644/768	83	18010	5BS4-0.43-0.56
									5AS1-0.40-0.75
									C-5DS1-0.63
340	m03183|LOC_Os12g32630	F-box protein family,putative	1618	603	e-173	602/799	75	9896	5BL6-0.29-0.75*
									C-5DL1-0.60
									5AL12-0.35-0.57
341	m03184|LOC_Os12g32630	F-box protein family,putative	1885	540	e-153	552/739	74	9896	5BL6-0.29-0.75*
									C-5DL1-0.60
									5AL12-0.35-0.57
342	m03186|LOC_Os12g32650	expressed protein	5335	507	e-143	395/471	83	13119	5AS1-0.40-0.75
									C-5DS1-0.63
									5BS4-0.43-0.56
343	m03198|LOC_Os12g32760	Sugar transporter	2001	228	1e-59	361/564	64	13040	4BL5-0.86-1.00
									4AS3-0.76-1.00
									4DL13-0.56-0.71

344	m03207|LOC_Os12g32850	cytochrome p450 71e1 (ec1.14.13.68)		175	6e-44	243/366	66	11098	C-6BL3-0.36
345	m03216|LOC_Os12g32940	sorbitol transporter	1855	305	5e-83	382/567	67	13040	4BL5-0.86-1.00
									4AS3-0.76-1.00
									4DL13-0.56-0.71
346	m03217|LOC_Os12g32950	hypothetical protein	553	277	5e-75	198/222	89	13121	5AS1-0.40-0.75
347	m03219|LOC_Os12g32970	hypothetical protein	585	120	1e-27	206/327	62	763	1DL2-0.41-1.00
348	m03220|LOC_Os12g32980	Potyvirus VPg interactingprotein	2379	120	4e-27	288/501	57	14711	4BL1-0.71-0.86
349	m03224|LOC_Os12g33020	AT4g26670/F10M23_10	996	584	e-167	488/598	81	16598	6BL5-0.40-1.00
350	m03229|LOC_Os12g33070	Similar to probabletranscription factor (MYB46) - Arabidopsis thaliana	1230	271	1e-72	246/322	76	3976	6BL
									6BL
									C-6DL6-0.29
									C-6AL4-0.55
351	m03230|LOC_Os12g33080	2-oxoglutarate/malatetranslocator precursor-like protein	1959	1012	0.0	788/938	84	8584	C-5DS1-0.63
									5BS4-0.43-0.56
									5AS1-0.40-0.75
352	m03240|LOC_Os12g33180	At5g27860	639	100	2e-21	81/100	81	9799	C-5AS1-0.40
									C-5DS1-0.63
									5BS4-0.43-0.56
353	m03283|LOC_Os12g33610	phenylalanine ammonia-lyase	2318	1591	0.0	1562/2076	75	18718	6BL3-0.36-0.40
									6DL6-0.29-0.47
									C-6AL4-0.55
354	m03368|LOC_Os12g34440	expressed protein	1829	228	1e-59	442/697	63	10646	1DL2-0.41-1.00
									1DL2-0.41-1.00
355	m03374|LOC_Os12g34500	hypothetical protein	1536	169	4e-42	211/316	66	5370	C-4DL9-0.31
									4AS1-0.20-0.63
356	m03375|LOC_Os12g34510	histone h2a. [norwayspruce, picea excelsa	417	496	e-141	363/409	88	15929	7AL16-0.86-0.90
									7DL3-0.82-1.00
									7AL16-0.86-0.90
									7DL3-0.82-1.00
									7BL10-0.78-1.00
357	m03377|LOC_Os12g34530	Peroxidase, putative	651	123	1e-28	170/248	68	13267	4AS1-0.20-0.63
									C-4BL1-0.71

									4DL9-0.31-0.56
									4DL9-0.31-0.56
358	m03406|LOC_Os12g34820	hypothetical protein	675	112	3e-25	127/179	70	8754	5AL23-0.87-1.00
									5DL5-0.76-1.00
									5AL23-0.87-1.00
359	m03408|LOC_Os12g34840	Similar toGTPase-activating protein homolog T4I9.2 - Arabidopsis thaliana	1651	465	e-131	487/681	71	15039	5BL1-0.55-0.75
360	m03413|LOC_Os12g34890	acyl carrier protein,putative	450	386	e-108	363/468	77	16186	5AS1-0.40-0.75
									5BS4-0.43-0.56
									C-5DS1-0.63
361	m03423|LOC_Os12g34990	hypothetical protein	738	225	4e-59	169/197	85	4318	4AS4-0.63-0.76
									4AS4-0.63-0.76
									4DL9-0.31-0.56
									C-4BL1-0.71
									C-4BL1-0.71
362	m03427|LOC_Os12g35030	At1g61040/T7P1_17	2192	822	0.0	636/749	84	13256	5AS1-0.40-0.75
363	m03428|LOC_Os12g35030	At1g61040/T7P1_17	2214	822	0.0	636/749	84	13256	5AS1-0.40-0.75
364	m03429|LOC_Os12g35040	Metalloenzyme superfamily,putative	1771	478	e-135	380/459	82	9843	5AS1-0.40-0.75
365	m03452|LOC_Os12g35270	expressed protein	927	312	3e-85	276/341	80	10820	5BS8-0.56-0.71
									5AS1-0.40-0.75
									5AS1-0.40-0.75
									5AS1-0.40-0.75
366	m03454|LOC_Os12g35290	Dor1-like family, putative	2143	989	0.0	716/804	89	11549	5AS
									5BS4-0.43-0.56
									5BS4-0.43-0.56
									5A
367	m03457|LOC_Os12g35320	CHY zinc finger, putative	1326	217	2e-56	358/570	62	16859	3AL3-0.42-0.78
									3BL2-0.22-0.50
									3DL2-0.27-0.81
									3BL2-0.22-0.50
368	m03460|LOC_Os12g35350	RanGAP1 interacting protein	2595	136	8e-32	107/129	82	14189	4B
369	m03480|LOC_Os12g35550	expressed protein	783	467	e-132	442/570	77	12236	2DL3-0.49-0.76
									2BL2-0.36-0.50
									C-2AL1-0.85

370	m03489|LOC_Os12g35610	respiratory burst oxidasehomolog	3147	274	3e-73	441/686	64	5373	5AL23-0.87-1.00
371	m03490|LOC_Os12g35610	respiratory burst oxidasehomolog	3160	274	3e-73	441/686	64	5373	5AL23-0.87-1.00
372	m03533|LOC_Os12g36040	Pollen allergen, putative	1173	128	9e-30	130/180	72	14223	5BL9-0.76-0.79
									5DL5-0.76-1.00
									5AL23-0.87-1.00
373	m03547|LOC_Os12g36180	expressed protein	3643	1033	0.0	783/905	86	12591	5AS1-0.40-0.75
									5BS4-0.43-0.56
									C-5DS1-0.63
374	m03549|LOC_Os12g36200	Nucleoside diphosphatekinase	732	510	e-145	415/499	83	9962	5A
375	m03550|LOC_Os12g36210	subtilisin/chymotrypsininhibitor - maize	509	166	1e-41	145/186	77	16076	1AS1-0.47-0.86
									1BS.sat18-0.50-1.00
									1DS5-0.70-1.00
									1DS5-0.70-1.00
376	m03551|LOC_Os12g36220	CI2E	505	160	1e-39	151/197	76	16076	1AS1-0.47-0.86
									1BS.sat18-0.50-1.00
									1DS5-0.70-1.00
									1DS5-0.70-1.00
377	m03553|LOC_Os12g36240	CI2E	512	168	4e-42	156/207	75	16076	1AS1-0.47-0.86
									1BS.sat18-0.50-1.00
									1DS5-0.70-1.00
									1DS5-0.70-1.00
378	m03579|LOC_Os12g36490	Patatin-like phospholipase,putative	3891	500	e-141	758/1156	65	13614	C-2BL2-0.36
									C-2DL3-0.49
379	m03583|LOC_Os12g36530	Patatin-like phospholipase,putative	1434	497	e-141	753/1158	65	13614	C-2BL2-0.36
									C-2DL3-0.49
380	m03591|LOC_Os12g36610	Patatin-like phospholipase,putative	1522	310	2e-84	452/679	66	13614	C-2BL2-0.36
									C-2DL3-0.49
381	m03597|LOC_Os12g36670	Similar to At5g27920	2287	1047	0.0	859/1053	81	12806	5BS4-0.43-0.56
									5AS1-0.40-0.75
382	m03612|LOC_Os12g36810	hypothetical protein	1284	250	2e-66	325/488	66	17414	5BL16-0.79-1.00
									4AL5-0.66-0.80
									5DL5-0.76-1.00

									5BL16-0.79-1.00
383	m03620|LOC_Os12g36890	Similar to cellulosesynthase-like protein	3648	464	e-130	455/613	74	1112	C-2AL1-0.85
									1DS5-0.70-1.00
									1A
384	m03622|LOC_Os12g36910	expressed protein	1972	402	e-112	390/517	75	728	5BS4-0.43-0.56
									C-5DS1-0.63
									5AS1-0.40-0.75
385	m03623|LOC_Os12g36910	expressed protein	2149	402	e-112	390/517	75	728	5BS4-0.43-0.56
									C-5DS1-0.63
									5AS1-0.40-0.75
386	m03627|LOC_Os12g36950	CTP synthase	2160	355	9e-98	635/1042	60	18811	1AL3-0.61-1.00
									1BL3-0.85-1.00
387	m03628|LOC_Os12g36950	CTP synthase	2119	355	9e-98	635/1042	60	18811	1AL3-0.61-1.00
									1BL3-0.85-1.00
388	m03629|LOC_Os12g36950	CTP synthase	2100	355	9e-98	635/1042	60	18811	1AL3-0.61-1.00
									1BL3-0.85-1.00
389	m03640|LOC_Os12g37060	At3g62580/T12C14_280	984	361	e-100	280/323	86	11664	C-5AS1-0.40
390	m03660|LOC_Os12g37260	Lipoxygenase	2746	1223	0.0	1147/1493	76	15436	7AS1-0.89-1.00
									7DS4-0.61-1.00
391	m03661|LOC_Os12g37260	Lipoxygenase	3080	1223	0.0	1147/1493	76	15436	7AS1-0.89-1.00
									7DS4-0.61-1.00
392	m03667|LOC_Os12g37320	Similar to lipoxygenase	1167	163	3e-40	265/413	64	15436	7AS1-0.89-1.00
									7DS4-0.61-1.00
393	m03670|LOC_Os12g37350	Lipoxygenase	1353	838	0.0	715/892	80	15436	7AS1-0.89-1.00
									7DS4-0.61-1.00
394	m03671|LOC_Os12g37360	ADP-ribosylation factorfamily	996	580	e-166	475/585	81	15788	7DL3-0.82-1.00
									C-3DS3-0.24
									3B
									5BL9-0.76-0.79
									7AL18-0.90-1.00
395	m03672|LOC_Os12g37370	RNA Polymerase II subunit14.5 kD, putative	882	439	e-123	314/352	89	6424	5BS4-0.43-0.56
									C-5DS1-0.63
									5AS1-0.40-0.75

396	m03675|LOC_Os12g37400	replication licensingfactor MCM7 homologue	2670	2753	0.0	1974/2207	89	17513	5AS1-0.40-0.75
									5BS4-0.43-0.56
									C-5DS1-0.63
397	m03676|LOC_Os12g37410	ocs-element binding factor1	1266	484	e-137	376/432	87	17079	3A
									3DL2-0.27-0.81
									C-3BL10-0.50*
398	m03678|LOC_Os12g37430	PAPA-1-like conservedregion, putative	2234	559	e-159	601/830	72	12772	5A
									5BS4-0.43-0.56
399	m03679|LOC_Os12g37430	PAPA-1-like conservedregion, putative	2305	559	e-159	601/830	72	12772	5A
									5BS4-0.43-0.56
400	m03687|LOC_Os12g37510	UDP-glucoronosyl andUDP-glucosyl transferase	1834	261	1e-69	263/362	72	7438	C-3AL5-0.78*
									C-3BL2-0.22
									5DL9-0.74-0.76
401	m03689|LOC_Os12g37530	expressed protein	2297	730	0.0	698/901	77	12543	5BS4-0.43-0.56
									C-5DS1-0.63
									C-5AS3-0.75*
402	m03690|LOC_Os12g37530	expressed protein	2143	730	0.0	698/901	77	12543	5BS4-0.43-0.56
									C-5DS1-0.63
									C-5AS3-0.75*
403	m03691|LOC_Os12g37530	expressed protein	2192	730	0.0	698/901	77	12543	5BS4-0.43-0.56
									C-5DS1-0.63
									C-5AS3-0.75*
404	m03697|LOC_Os12g37580	ABC transporter, putative	3684	389	e-108	439/633	69	13087	7DS4-0.61-1.00
									7BS1-0.27-1.00
									7DS4-0.61-1.00
									7AS8-0.45-0.89*
405	m03699|LOC_Os12g37600	Acyltransferase, putative	1677	313	2e-85	358/499	71	6217	2BL
									2AL
406	m03706|LOC_Os12g37650	expressed protein	1053	145	4e-35	239/382	62	17931	3BS1-0.33-0.57
									3BS8-0.78-1.00
									3BL7-0.63-1.00
									3DS3-0.24-0.55

									3BS8-0.78-1.00
									3AS4-0.45-1.00
									3AS4-0.45-1.00
									3BS8-0.78-1.00
									3BS8-0.78-1.00
									3BL7-0.63-1.00
407	m03710|LOC_Os12g37690	Myb-like DNA-bindingdomain, putative	903	155	5e-38	198/299	66	3976	6BL
									6BL
									C-6DL6-0.29
									C-6AL4-0.55
408	m03716|LOC_Os12g37740	NB-ARC domain, putative	3330	191	2e-48	262/395	66	4054	6DL10-0.80-1.00
									6BL5-0.40-1.00
									6AL8-0.90-1.00
409	m03717|LOC_Os12g37750	hypothetical protein	1548	112	7e-25	265/440	60	12681	3DS3-0.24-0.55
									3BS1-0.33-0.57
									3DS3-0.24-0.55
									3BS1-0.33-0.57
410	m03729|LOC_Os12g37870	expressed protein	1909	1369	0.0	1072/1281	83	18131	4BS4-0.37-0.57
411	m03730|LOC_Os12g37880	expressed protein	707	418	e-117	372/466	79	17329	5BS4-0.43-0.56
412	m03735|LOC_Os12g37920	Similar toAT3g09470/F11F8_4	1233	142	5e-34	187/285	65	8951	5BS6-0.81-1.00
									5AS3-0.75-0.98
									5DS2-0.78-1.00
413	m03736|LOC_Os12g37930	Similar toAt3g09470/F11F8_4	1383	226	3e-59	266/390	68	8951	5BS6-0.81-1.00
									5AS3-0.75-0.98
									5DS2-0.78-1.00
414	m03740|LOC_Os12g37970	transcription factor,putative	1167	581	e-166	613/815	75	13842	C-4DS1-0.53
									C-4AL12-0.43
415	m03741|LOC_Os12g37980	Protein kinase domain,putative	2913	109	1e-23	171/269	63	2609	5BS4-0.43-0.56
									4AL4-0.80-1.00
416	m03744|LOC_Os12g38010	Similar to plantmetallothionein-like protein	509	125	3e-29	148/213	69	16897	1BL1-0.47-0.69
									1BL1-0.47-0.69
									1DL2-0.41-1.00
									1BL1-0.47-0.69
									1AL1-0.17-0.61

									1BL1-0.47-0.69
									1AL1-0.17-0.61
									1BL1-0.47-0.69
									1DL2-0.41-1.00
									1DL2-0.41-1.00
									1BL1-0.47-0.69
417	m03748|LOC_Os12g38050	Metallothionein	533	133	1e-31	171/249	68	16897	1BL1-0.47-0.69
									1BL1-0.47-0.69
									1DL2-0.41-1.00
									1BL1-0.47-0.69
									1AL1-0.17-0.61
									1BL1-0.47-0.69
									1AL1-0.17-0.61
									1BL1-0.47-0.69
									1DL2-0.41-1.00
									1DL2-0.41-1.00
									1BL1-0.47-0.69
418	m03751|LOC_Os12g38080	Metallothionein	515	129	1e-30	167/243	68	16897	1BL1-0.47-0.69
									1BL1-0.47-0.69
									1DL2-0.41-1.00
									1BL1-0.47-0.69
									1AL1-0.17-0.61
									1BL1-0.47-0.69
									1AL1-0.17-0.61
									1BL1-0.47-0.69
									1DL2-0.41-1.00
									1DL2-0.41-1.00
									1BL1-0.47-0.69
419	m03757|LOC_Os12g38140	hypothetical protein	825	277	8e-75	280/386	72	15250	5AS3-0.75-0.98
									5S
									5BS4-0.43-0.56
									6DS6-0.99-1.00
									6BS-Sat
									2DS5-0.47-1.00

420	m03761|LOC_Os12g38180	dnaK protein	1200	137	1e-32	276/441	62	17156	6BS-Sat
									6AS5-0.65-1.00
421	m03772|LOC_Os12g38290	Similar tometallothionein-like protein type 1. [rice	231	131	2e-31	152/217	70	16897	1BL1-0.47-0.69
									1BL1-0.47-0.69
									1DL2-0.41-1.00
									1BL1-0.47-0.69
									1AL1-0.17-0.61
									1BL1-0.47-0.69
									1AL1-0.17-0.61
									1BL1-0.47-0.69
									1DL2-0.41-1.00
									1DL2-0.41-1.00
									1BL1-0.47-0.69
422	m03773|LOC_Os12g38300	Metallothionein	479	128	3e-30	172/254	67	16897	1BL1-0.47-0.69
									1BL1-0.47-0.69
									1DL2-0.41-1.00
									1BL1-0.47-0.69
									1AL1-0.17-0.61
									1BL1-0.47-0.69
									1AL1-0.17-0.61
									1BL1-0.47-0.69
									1DL2-0.41-1.00
									1DL2-0.41-1.00
									1BL1-0.47-0.69
423	m03774|LOC_Os12g38310	mitochondrial import innermembrane translocase subunit tim9	700	355	3e-98	319/400	79	11650	5AS1-0.40-0.75
									C-5DS1-0.63
									5BS4-0.43-0.56
									5BS4-0.43-0.56
424	m03785|LOC_Os12g38420	hypothetical protein	605	263	1e-70	224/278	80	16886	C-5AS3-0.75*
									C-5DS1-0.63
									C-5DS1-0.63
425	m03786|LOC_Os12g38430	RNA recognition motif.(a.k.a. RRM, RBD, or RNP domain), putative	1253	854	0.0	681/799	85	16886	C-5AS3-0.75*

									C-5DS1-0.63
									C-5DS1-0.63
426	m03787|LOC_Os12g38440	XH domain, putative	1562	207	2e-53	289/438	65	7796	5AS3-0.75-1.00*
									C-5DS1-0.63
									C-5BS8-0.56*
427	m03788|LOC_Os12g38440	XH domain, putative	1528	207	2e-53	289/438	65	7796	5AS3-0.75-1.00*
									C-5DS1-0.63
									C-5BS8-0.56*
428	m03805|LOC_Os12g38610	lectin-like receptor kinase7;3	2061	575	e-164	801/1200	66	16028	1BL2-0.69-1.00*
									4AL12-0.43-0.59
									4DS1-0.53-0.67
									C-6AL4-0.55
									1AL3-0.61-1.00
									1AL3-0.61-1.00
									1DL
									1BL2-0.69-1.00*
429	m03811|LOC_Os12g38640	At5g08050/F13G24_250	924	364	e-101	384/494	77	2571	
									C-3BL2-0.22
430	m03812|LOC_Os12g38640	At5g08050/F13G24_250	780	215	3e-56	278/376	73	2571	
									C-3BL2-0.22
431	m03821|LOC_Os12g38730	expressed protein	2379	778	0.0	665/830	80	1128	5AS
									5BS
									C-5DS1-0.63
									5BS4-0.43-0.56
									5AS
432	m03822|LOC_Os12g38730	expressed protein	2395	805	0.0	671/830	80	1128	5AS
									5BS
									C-5DS1-0.63
									5BS4-0.43-0.56
									5AS
433	m03824|LOC_Os12g38750	diphosphonucleotidephosphatase 1	2236	569	e-162	426/495	86	667	5BS4-0.43-0.56
									C-5DS1-0.63
434	m03825|LOC_Os12g38750	diphosphonucleotidephosphatase 1	2138	569	e-162	426/495	86	667	5BS4-0.43-0.56
									C-5DS1-0.63

435	m03826|LOC_Os12g38760	diphosphonucleotidephosphatase 1	2106	549	e-156	420/495	84	667	5BS4-0.43-0.56
									C-5DS1-0.63
436	m03827|LOC_Os12g38760	diphosphonucleotidephosphatase 1	2219	549	e-156	420/495	84	667	5BS4-0.43-0.56
									C-5DS1-0.63
437	m03828|LOC_Os12g38770	diphosphonucleotidephosphatase 1	1695	404	e-112	374/495	75	667	5BS4-0.43-0.56
									C-5DS1-0.63
438	m03845|LOC_Os12g38930	Glycosyl transferase family8, putative	2751	141	3e-33	220/342	64	13288	1DL2-0.41-1.00
									C-2DL3-0.49
									1AL3-0.61-1.00
									1BL2-0.69-0.85
439	m03865|LOC_Os12g39120	Protein phosphatase 2C,putative	1738	261	1e-69	350/531	65	12229	5DL5-0.76-1.00
									5AL10-0.57-0.78
440	m03869|LOC_Os12g39160	expressed protein	2084	316	3e-86	365/501	72	11588	4AL12-0.43-0.59
									4B
									4D
									4D
441	m03870|LOC_Os12g39170	probable serinecarboxypeptidase I [imported] - Arabidopsis thaliana	1362	244	1e-64	349/525	66	8542	C-4AL12-0.43
									C-4BS4-0.37
442	m03873|LOC_Os12g39200	expressed protein	1871	629	e-180	649/862	75	14385	5AS3-0.75-0.98
									5AS3-0.75-0.98
									5BS4-0.43-0.56
443	m03874|LOC_Os12g39200	expressed protein	1911	549	e-156	485/614	78	14385	5AS3-0.75-0.98
									5AS3-0.75-0.98
									5BS4-0.43-0.56
444	m03875|LOC_Os12g39210	Cyclin, N-terminal domain,putative	1620	773	0.0	803/1079	74	16528	C-4AL12-0.43
									C-4BL1-0.71
									C-4DL9-0.31
									2BS1-0.53-0.75
									1BL1-0.47-0.69
									C-4BL1-0.71
445	m03878|LOC_Os12g39240	Cytochrome P450	1635	177	2e-44	284/452	62	16621	1DL4-0.18-1.00*
									1BL1-0.47-0.69

446	m03884|LOC_Os12g39300	cytochrome P450, putative	1713	185	8e-47	316/506	62	16621	1DL4-0.18-1.00*
									1BL1-0.47-0.69
447	m03890|LOC_Os12g39360	Eukaryotic aspartylprotease	1341	193	3e-49	425/689	61	16826	6BL5-0.40-1.00
448	m03897|LOC_Os12g39420	probable membranetransporter At2g26510 [imported] - Arabidopsis thaliana	2526	691	0.0	658/836	78	12096	5BS8-0.56-0.71
									5AS3-0.75-0.98
									C-5DS1-0.63
449	m03898|LOC_Os12g39420	probable membranetransporter At2g26510 [imported] - Arabidopsis thaliana	2433	691	0.0	658/836	78	12096	5BS8-0.56-0.71
									5AS3-0.75-0.98
									C-5DS1-0.63
450	m03899|LOC_Os12g39420	probable membranetransporter At2g26510 [imported] - Arabidopsis thaliana	2370	691	0.0	658/836	78	12096	5BS8-0.56-0.71
									5AS3-0.75-0.98
									C-5DS1-0.63
451	m03900|LOC_Os12g39420	probable membranetransporter At2g26510 [imported] - Arabidopsis thaliana	2490	691	0.0	658/836	78	12096	5BS8-0.56-0.71
									5AS3-0.75-0.98
									C-5DS1-0.63
452	m03901|LOC_Os12g39420	probable membranetransporter At2g26510 [imported] - Arabidopsis thaliana	2125	691	0.0	658/836	78	12096	5BS8-0.56-0.71
									5AS3-0.75-0.98
									C-5DS1-0.63
453	m03902|LOC_Os12g39420	probable membranetransporter At2g26510 [imported] - Arabidopsis thaliana	2104	691	0.0	658/836	78	12096	5BS8-0.56-0.71
									5AS3-0.75-0.98
									C-5DS1-0.63
454	m03923|LOC_Os12g39620	Similar to diseaseresistance-like protein - Arabidopsis thaliana	2691	1023	0.0	768/891	86	12836	5BS8-0.56-0.71
455	m03924|LOC_Os12g39630	protein kinase-like protein	2535	527	e-149	560/788	71	16726	2D
									2BS
456	m03927|LOC_Os12g39660	calcium-translocatingP-type ATPase, PMCA-type	3666	614	e-176	586/745	78	12004	C-5DS1-0.63
									5AS3-0.75-0.98
									5BS8-0.56-0.71

457	m03932|LOC_Os12g39710	expressed protein	2667	134	2e-31	240/386	62	12681	3DS3-0.24-0.55
									3BS1-0.33-0.57
									3DS3-0.24-0.55
									3BS1-0.33-0.57
458	m03933|LOC_Os12g39710	expressed protein	1672	134	2e-31	240/386	62	12681	3DS3-0.24-0.55
									3BS1-0.33-0.57
									3DS3-0.24-0.55
									3BS1-0.33-0.57
459	m03945|LOC_Os12g39830	Cyclin, N-terminal domain,putative	1522	527	e-150	551/700	78	8307	C-5DS1-0.63
									5BS8-0.56-0.71
460	m04001|LOC_Os12g40370	F-box domain, putative	2067	104	2e-22	85/105	80	16535	4DL13-0.56-0.71
461	m04012|LOC_Os12g40480	hypothetical protein	1812	702	0.0	543/644	84	3154	2DS1-0.33-0.47
462	m04015|LOC_Os12g40510	EF hand, putative	1132	505	e-143	368/413	89	16712	5BS8-0.56-0.71
									C-5DS1-0.63
									C-5AS1-0.40
463	m04023|LOC_Os12g40570	WRKY transcription factor 1	2034	530	e-150	484/614	78	16503	4BS1-0.81-1.00
									4DS2-0.82-1.00
464	m04024|LOC_Os12g40570	WRKY transcription factor 1	2113	573	e-163	524/667	78	16503	4BS1-0.81-1.00
									4DS2-0.82-1.00
465	m04044|LOC_Os12g40770	ankyrin-like protein-likeprotein	1760	454	e-128	477/653	73	15517	5BS4-0.43-0.56
466	m04045|LOC_Os12g40780	ankyrin-like protein-likeprotein	1563	510	e-144	458/595	76	15517	5BS4-0.43-0.56
467	m04056|LOC_Os12g40880	Phosphoribulokinase /Uridine kinase family, putative	1752	225	1e-58	184/227	81	1912	5BS5-0.71-0.81
									5DS1-0.63-0.67
									C-5AS1-0.40
									2AL
									2BL
									5DS1-0.63-0.67
									5BS5-0.71-0.81
									5AS3-0.75-0.98
468	m04057|LOC_Os12g40880	Phosphoribulokinase /Uridine kinase family, putative	1305	225	7e-59	184/227	81	1912	5BS5-0.71-0.81
									5DS1-0.63-0.67
									C-5AS1-0.40

									2AL
									2BL
									5DS1-0.63-0.67
									5BS5-0.71-0.81
									5AS3-0.75-0.98
469	m04058|LOC_Os12g40890	aux/IAA protein	1572	451	e-127	333/377	88	14899	5BS5-0.71-0.81
									5DS1-0.63-0.67
									5AS3-0.75-0.98
470	m04059|LOC_Os12g40900	AUX/IAA family	1163	141	1e-33	303/483	62	16443	5BL9-0.76-0.79
471	m04079|LOC_Os12g41090	Protein kinase domain,putative	1378	237	1e-62	338/507	66	2582	3DL2-0.27-0.81
									3AL3-0.42-0.78
									3AL3-0.42-0.78
472	m04081|LOC_Os12g41110	EF hand, putative	886	218	4e-57	166/195	85	16205	1AL3-0.61-1.00
473	m04087|LOC_Os12g41170	Root hair defective 3GTP-binding protein (RHD3)	3085	700	0.0	1133/1820	62	17731	C-3DL2-0.27
474	m04088|LOC_Os12g41180	Protein kinase domain,putative	2327	1147	0.0	894/1055	84	9951	5BS8-0.56-0.71
									5AS3-0.75-0.98
									5DS1-0.63-0.67
									6DL6-0.29-0.47
475	m04092|LOC_Os12g41220	probableubiquitin-conjugating enzyme [imported] - Arabidopsis thaliana	966	424	e-119	344/421	81	15842	C-5BL14-0.75*
476	m04099|LOC_Os12g41260	protein kinase ATMRK1 (EC2.7.1.-) [imported] - Arabidopsis thaliana	1692	672	0.0	754/1055	71	13501	5DL5-0.76-1.00
									5AL10-0.57-0.78
									5BL9-0.76-0.79
477	m04100|LOC_Os12g41270	serine/threonine proteinkinase	2817	188	2e-47	382/611	62	16028	1BL2-0.69-1.00*
									4AL12-0.43-0.59
									4DS1-0.53-0.67
									C-6AL4-0.55
									1AL3-0.61-1.00
									1AL3-0.61-1.00
									1DL
									1BL2-0.69-1.00*
478	m04115|LOC_Os12g41400	translation initiationfactor eIF-2 gamma chain F20D22.6 - Arabidopsis thaliana	2077	1813	0.0	1358/1553	87	18249	1DL2-0.41-1.00

479	m04116|LOC_Os12g41410	receptor-like proteinkinase homolog RK20-1	1284	109	5e-24	120/167	71	16028	1BL2-0.69-1.00*
									4AL12-0.43-0.59
									4DS1-0.53-0.67
									C-6AL4-0.55
									1AL3-0.61-1.00
									1AL3-0.61-1.00
									1DL
									1BL2-0.69-1.00*
480	m04126|LOC_Os12g41490	Protein kinase domain,putative	2792	336	6e-92	445/674	66	16028	1BL2-0.69-1.00*
									4AL12-0.43-0.59
									4DS1-0.53-0.67
									C-6AL4-0.55
									1AL3-0.61-1.00
									1AL3-0.61-1.00
									1DL
									1BL2-0.69-1.00*
481	m04127|LOC_Os12g41500	thiosulfatesulfurtransferase (EC 2.8.1.1) [validated] - Arabidopsis thaliana	1221	1147	0.0	923/1093	84	15653	5AS3-0.75-0.98
									5BS8-0.56-0.71
									5DS1-0.63-0.67
482	m04128|LOC_Os12g41510	Protein kinase domain,putative	1971	307	2e-83	547/881	62	16028	1BL2-0.69-1.00*
									4AL12-0.43-0.59
									4DS1-0.53-0.67
									C-6AL4-0.55
									1AL3-0.61-1.00
									1AL3-0.61-1.00
									1DL
									1BL2-0.69-1.00*
483	m04130|LOC_Os12g41530	Protein kinase domain,putative	2049	294	1e-79	421/652	64	16028	1BL2-0.69-1.00*
									4AL12-0.43-0.59
									4DS1-0.53-0.67
									C-6AL4-0.55
									1AL3-0.61-1.00

									1AL3-0.61-1.00
									1DL
									1BL2-0.69-1.00*
484	m04131|LOC_Os12g41540	serine/threonine proteinkinase	1314	280	2e-75	516/801	64	16028	1BL2-0.69-1.00*
									4AL12-0.43-0.59
									4DS1-0.53-0.67
									C-6AL4-0.55
									1AL3-0.61-1.00
									1AL3-0.61-1.00
									1DL
									1BL2-0.69-1.00*
485	m04147|LOC_Os12g41680	salicylic acid-inducedprotein 19	1260	307	1e-83	365/517	70	4891	7DL2-0.61-0.82
									4DS2-0.82-1.00
									4AL12-0.43-0.59
									4BS1-0.81-1.00
									2DS5-0.47-1.00
									2AS5-0.78-1.00
									7BL7-0.63-0.78
									7DL2-0.61-0.82
486	m04149|LOC_Os12g41700	zinc finger protein LSD1	1186	383	e-106	396/536	73	14754	5DL5-0.76-1.00
									5DL5-0.76-1.00
									5AL10-0.57-0.78
									5BL9-0.76-0.79
									5AL10-0.57-0.78
487	m04150|LOC_Os12g41710	DEAD/DEAH box helicase,putative	3293	133	9e-31	277/451	61	12334	5AS1-0.40-0.75
488	m04162|LOC_Os12g41820	S-locus protein 5	3103	844	0.0	692/827	83	12277	5AS3-0.75-0.98
									5BS
									5DS1-0.63-0.67
489	m04173|LOC_Os12g41920	DNA-binding protein PcMYB1,putative	1672	431	e-121	424/552	76	8098	5AS7/10-0.98-1.00
									5BS5-0.71-0.81
									5DS1-0.63-0.67
490	m04174|LOC_Os12g41920	DNA-binding protein PcMYB1,putative	1773	431	e-120	424/552	76	8098	5AS7/10-0.98-1.00

									5BS5-0.71-0.81
									5DS1-0.63-0.67
491	m04175|LOC_Os12g41920	DNA-binding protein PcMYB1,putative	1772	431	e-120	424/552	76	8098	5AS7/10-0.98-1.00
									5BS5-0.71-0.81
									5DS1-0.63-0.67
492	m04181|LOC_Os12g41960	T7N9 14 protein, putative	2700	153	5e-37	149/202	73	10316	1DL2-0.41-1.00
									1BL1-0.47-0.69
									5BL9-0.76-0.79
									5DL5-0.76-1.00
493	m04205|LOC_Os12g42190	transposon protein,putative, unclassified	1371	145	6e-35	144/192	75	4944	C-5BS4-0.43
494	m04210|LOC_Os12g42230	pyruvate dehydrogenase E1beta subunit	1534	156	3e-38	271/444	61	17317	C-5AL10-0.57*
									C-5BL14-0.75*
495	m04216|LOC_Os12g42280	viviparous-14 protein -maize	2465	131	2e-30	388/659	58	11924	6AL4-0.55-1.00*
									6BL5-0.40-1.00
									6DL6-0.29-0.47
496	m04225|LOC_Os12g42370	hypothetical protein	1797	917	0.0	872/1141	76	13815	5BL9-0.76-0.79
									5AL10-0.57-0.78
497	m04232|LOC_Os12g42440	Similar to dnaj proteinhomolog 2. [leek	1404	256	2e-68	460/749	61	17702	5DL5-0.76-1.00
									5AL10-0.57-0.78
498	m04240|LOC_Os12g42520	receptor kinase, putative	3165	110	4e-24	97/125	77	6898	C-7DL5-0.30
									7AL1-0.39-0.71
499	m04243|LOC_Os12g42550	methyl-binding domainprotein MBD106, putative	1651	446	e-125	353/425	83	16346	5BS6-0.81-1.00
									5DS2-0.78-1.00
500	m04249|LOC_Os12g42610	YABBY protein	1575	201	1e-51	162/193	83	17713	5AL
501	m04271|LOC_Os12g42810	Mov34/MPN/PAD-1 family,putative	1693	131	1e-30	115/148	77	11865	7BS1-0.27-1.00
									C-7AS8-0.45
									7DS5-0.36-0.61
502	m04274|LOC_Os12g42840	hypothetical protein	879	316	1e-86	316/418	75	2761	5AS3-0.75-0.98
									5DS2-0.78-1.00
									5DS2-0.78-1.00
503	m04278|LOC_Os12g42860	expressed protein	1370	435	e-122	536/778	68	14123	5DS2-0.78-1.00

504	m04279|LOC_Os12g42860	expressed protein	1471	450	e-126	584/860	67	14123	5DS2-0.78-1.00
505	m04282|LOC_Os12g42880	5-methyltetrahydropteroyltriglutamate--homocysteineS-methyltransferase	2956	2314	0.0	1781/2058	86	18820	5AS3-0.75-0.98
									5BS6-0.81-1.00
									5DS2-0.78-1.00
506	m04283|LOC_Os12g42890	5-methyltetrahydropteroyltriglutamate--homocysteineS-methyltransferase	2808	2903	0.0	2196/2521	87	18820	5AS3-0.75-0.98
									5BS6-0.81-1.00
									5DS2-0.78-1.00
507	m04284|LOC_Os12g42890	5-methyltetrahydropteroyltriglutamate--homocysteineS-methyltransferase	2689	2903	0.0	2196/2521	87	18820	5AS3-0.75-0.98
									5BS6-0.81-1.00
									5DS2-0.78-1.00
508	m04292|LOC_Os12g42970	Similar to NTL1 protein -curled-leaved tobacco	930	248	4e-66	209/257	81	1937	5AS3-0.75-0.98
									5BS6-0.81-1.00
									5DS2-0.78-1.00
509	m04293|LOC_Os12g42980	cysteine synthase A	993	145	4e-35	231/371	62	9252	3BL10-0.50-0.63
									3DL2-0.27-0.81
									3DL2-0.27-0.81
510	m04305|LOC_Os12g43100	expressed protein	1338	1101	0.0	813/932	87	16101	4DL9-0.31-0.56
									6BL5-0.40-1.00
									6DL10-0.80-1.00
									6DL10-0.80-1.00
									6DL10-0.80-1.00
511	m04330|LOC_Os12g43340	Cofilin/tropomyosin-typeactin-binding protein	831	225	5e-59	263/385	68	15915	4DL9-0.31-0.56
512	m04333|LOC_Os12g43370	methionine aminopeptidase,type II, putative	1807	1510	0.0	1101/1245	88	17314	C-7BS1-0.27
									7DS5-0.36-0.61
									3BL7-0.63-1.00
									3BL7-0.63-1.00
513	m04334|LOC_Os12g43380	thaumatin-like proteinprecursor	723	253	1e-67	286/398	71	16808	C-5BL14-0.75*
514	m04335|LOC_Os12g43390	thaumatin-like proteinprecursor	525	228	3e-60	271/384	70	16808	C-5BL14-0.75*
515	m04339|LOC_Os12g43430	thaumatin-like proteinprecursor	795	259	1e-69	305/432	70	16808	C-5BL14-0.75*
516	m04340|LOC_Os12g43440	thaumatin-like proteinprecursor	747	275	2e-74	305/422	72	16808	C-5BL14-0.75*

517	m04341|LOC_Os12g43450	thaumatin-like protein	883	591	e-169	547/697	78	16808	C-5BL14-0.75*
518	m04345|LOC_Os12g43490	thaumatin-like protein TLP7	920	589	e-168	527/658	80	16808	C-5BL14-0.75*
519	m04356|LOC_Os12g43600	Similar to glycine-richRNA-binding protein - rice	814	448	e-126	430/548	78	18748	3DL2-0.27-0.81
520	m04358|LOC_Os12g43620	hypothetical protein	1017	112	4e-25	88/101	87	4156	7BL10-0.78-1.00
									7BL10-0.78-1.00
									7AL21-0.74-0.86
									C-7DL5-0.30
									7BL10-0.78-1.00
521	m04359|LOC_Os12g43630	malate dehydrogenase,NAD-dependent	1345	847	0.0	782/1030	75	16424	3DL3-0.81-1.00
									2BS1-0.53-0.75
									6BL5-0.40-1.00
522	m04360|LOC_Os12g43630	malate dehydrogenase,NAD-dependent	1004	684	0.0	623/815	76	16424	3DL3-0.81-1.00
									2BS1-0.53-0.75
									6BL5-0.40-1.00
523	m04361|LOC_Os12g43640	hypothetical protein	3024	139	1e-32	198/299	66	12334	5AS1-0.40-0.75
524	m04363|LOC_Os12g43660	receptor protein kinase,putative	3539	152	2e-36	286/462	61	2609	5BS4-0.43-0.56
									4AL4-0.80-1.00
525	m04364|LOC_Os12g43670	hypothetical protein	2136	489	e-138	570/832	68	11212	2BS1-0.53-0.75
									2DS5-0.47-1.00
									2AS5-0.78-1.00
526	m04367|LOC_Os12g43700	pathogenesis-relatedprotein 1 - maize	1594	133	4e-31	222/351	63	13000	4DL12-0.71-1.00
									5AL23-0.87-1.00
									4BL5-0.86-1.00
527	m04368|LOC_Os12g43710	hypothetical protein	603	114	8e-26	154/227	67	3976	6BL
									6BL
									C-6DL6-0.29
									C-6AL4-0.55
528	m04370|LOC_Os12g43730	Myosin N-terminal SH3-likedomain, putative	2283	133	6e-31	89/95	93	17918	C-7DL5-0.30
									7A
									7A
									C-7DL5-0.30
529	m04381|LOC_Os12g43840	TPR Domain, putative	1816	137	2e-32	318/545	58	8303	5DL5-0.76-1.00

									5BL16-0.79-1.00
									5BL16-0.79-1.00
									5DL5-0.76-1.00
									5BL16-0.79-1.00
									5AL17-0.78-0.87
									5BL16-0.79-1.00
530	m04390|LOC_Os12g43930	Zinc finger, C3HC4 type(RING finger), putative	1158	334	8e-92	384/533	72	8609	4AL13-0.59-0.66
									4BS1-0.81-1.00
									4DS2-0.82-1.00
531	m04391|LOC_Os12g43940	ankyrin-like protein-likeprotein	1720	118	9e-27	181/283	63	2023	C-4BS4-0.37
									4DS
									4AL13-0.59-0.66
532	m04392|LOC_Os12g43950	hypothetical protein	2701	673	0.0	573/707	81	7227	5AS3-0.75-0.98
									5DS2-0.78-1.00
									5BS6-0.81-1.00
533	m04393|LOC_Os12g43950	expressed protein	3206	673	0.0	573/707	81	7227	5AS3-0.75-0.98
									5DS2-0.78-1.00
									5BS6-0.81-1.00
534	m04395|LOC_Os12g43970	epoxide hydrolase-likeprotein - Arabidopsis thaliana	1215	202	3e-52	254/376	67	6863	3BS8-0.78-1.00
535	m04399|LOC_Os12g44010	secretory acid phosphataseprecursor	1383	180	2e-45	360/592	60	8465	C-5BL14-0.75*
									C-5AL10-0.57*
									7AL18-0.90-1.00
536	m04400|LOC_Os12g44020	secretory acid phosphataseprecursor	1852	148	9e-36	360/602	59	8465	C-5BL14-0.75*
									C-5AL10-0.57*
									7AL18-0.90-1.00
537	m04401|LOC_Os12g44030	truncated putative purpleacid phosphatase	1014	158	6e-39	353/592	59	8465	C-5BL14-0.75*
									C-5AL10-0.57*
									7AL18-0.90-1.00
538	m04407|LOC_Os12g44090	receptor-like proteinkinase	2197	843	0.0	700/849	82	12334	5AS1-0.40-0.75
539	m04408|LOC_Os12g44100	POT family, putative	2246	188	1e-47	450/752	59	16668	1DL2-0.41-1.00
									1AL1-0.17-0.61

									1BL1-0.47-0.69
540	m04409|LOC_Os12g44110	probable peptidetransporter T17F3.11 [imported] - Arabidopsis thaliana	1986	209	7e-54	487/798	61	16668	1DL2-0.41-1.00
									1AL1-0.17-0.61
									1BL1-0.47-0.69
541	m04413|LOC_Os12g44150	plasma-membraneproton-efflux P-type ATPase	3290	2456	0.0	1774/1989	89	17621	5AS7/10-0.98-1.00
									5AS7/10-0.98-1.00
									5DS2-0.78-1.00
									5BS6-0.81-1.00
542	m04417|LOC_Os12g44190	contains similarity toAAA-type ATPase~gene_id:MYH19.21	1590	126	3e-29	142/200	71	1719	4AL12-0.43-0.59
									4DS3-0.67-0.82
543	m04419|LOC_Os12g44210	Similar to containssimilarity to AAA-type ATPase gene_id:MYH19.21	1500	120	3e-27	140/200	70	1719	4AL12-0.43-0.59
									4DS3-0.67-0.82
544	m04420|LOC_Os12g44220	Similar to mitochondrialprotein-like protein	969	128	7e-30	128/176	72	13819	3DS6-0.55-1.00
545	m04421|LOC_Os12g44230	hipl1 protein precursor	2236	440	e-123	419/546	76	10801	5AS3-0.75-0.98
									5BS6-0.81-1.00
									5DS2-0.78-1.00
546	m04427|LOC_Os12g44290	cytochrome P450, putative	1542	153	3e-37	253/395	64	11452	C-6AS1-0.35
									C-6BS5-0.76
547	m04431|LOC_Os12g44320	similar to eukaryoticprotein kinase domains	2697	126	6e-29	332/560	59	16028	1BL2-0.69-1.00*
									4AL12-0.43-0.59
									4DS1-0.53-0.67
									C-6AL4-0.55
									1AL3-0.61-1.00
									1AL3-0.61-1.00
									1DL
									1BL2-0.69-1.00*
548	m04433|LOC_Os12g44340	hypothetical protein	2331	226	5e-59	191/235	81	10608	5DS2-0.78-1.00
									5A
									5BS6-0.81-1.00
549	m04434|LOC_Os12g44350	Actin	1292	1117	0.0	886/1068	82	18589	4DS2-0.82-1.00

									4BS1-0.81-1.00
									4AL12-0.43-0.59
550	m04437|LOC_Os12g44380	sucrose/H+ symporter	1553	660	0.0	558/695	80	11692	5DS2-0.78-1.00
									5BL9-0.76-0.79
									5DS2-0.78-1.00
551	m04438|LOC_Os12g44380	sucrose/H+ symporter	2027	660	0.0	558/695	80	11692	5DS2-0.78-1.00
									5BL9-0.76-0.79
									5DS2-0.78-1.00
552	m04439|LOC_Os12g44390	Similar to SMC1 protein	3720	2041	0.0	1550/1803	85	15940	4DS3-0.67-0.82

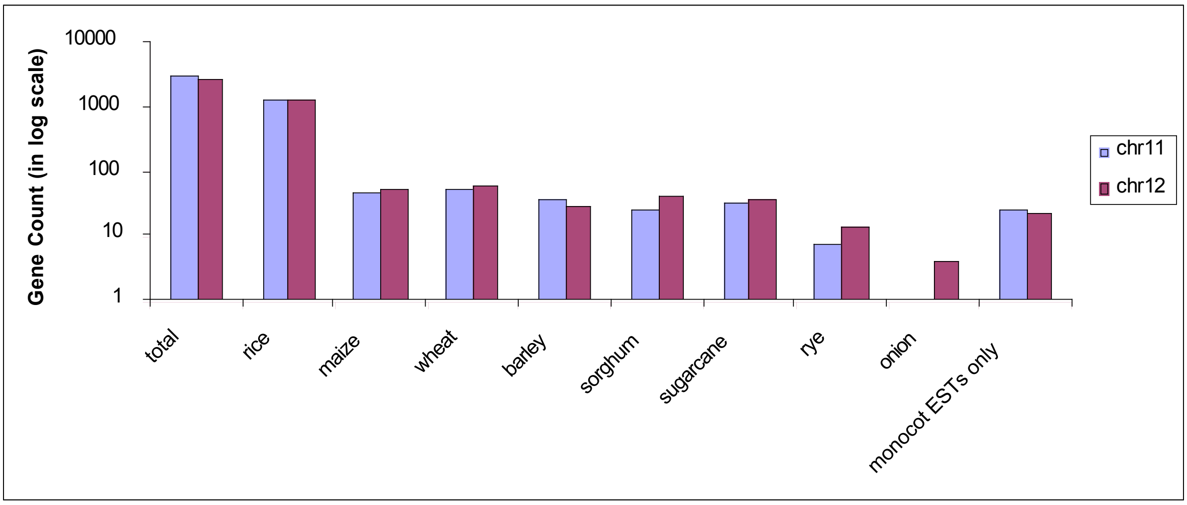

Figure 10. Rice genes with monocot EST matches. Non-TE-related genes from chromosome 11 and 12 with expression support based on alignment to the TIGR Gene Indices are depicted. The five gene indices used are rice, maize, wheat, barley, sorghum, sugarcane, rye, and onion.

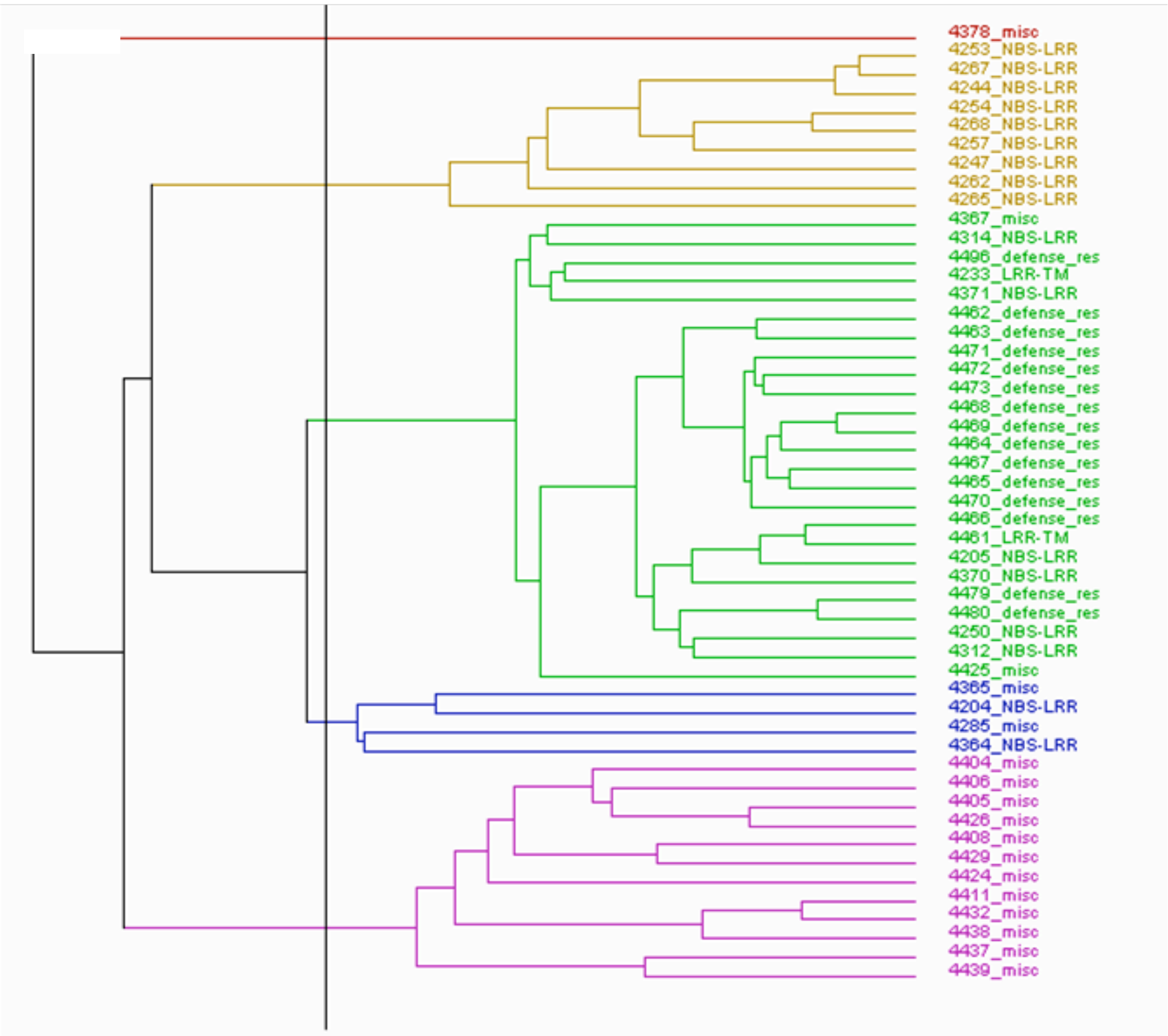

Figure 11. A cladogram of 52 R-like and defense response genes in the interval 112-119 cM on the long arm of chromosome 11. The tree was constructed based on the average distance method using the ClustalW software (http://www.ebi.ac.uk), with the predicted coding sequences of the genes. The genes are clearly separated into 5 different clades according to their predicted function and physical location (indicated by their numbers), except that some NBS-LRR genes grouped with the defense response genes category.

Figure 12. Distribution of rice gene models predicted from chromosome 11 on the 21 wheat chromosomes. Serial numbers on the left represent the gene order in rice according to supporting table 11. Each of the seven homoeologous groups of wheat chromosomes has been color-coded and matches with rice genes are represented by color filled cells. The values in cM represent positions defined by genetically mapped markers in the RGP map.

			Wheat homoeologous groups																				
	Rice chr 11		1			2			3			4			5			6			7		
S No.	cM	Gene model	A	B	D	A	B	D	A	B	D	A	B	D	A	B	D	A	B	D	A	B	D
1	0.0	M00017																					
2		M00019																					
3		M00033																					
4		M00036																					
5		M00037																					
6		M00038																					
7		M00042																					
8		M00057																					
9		M00062																					
10		M00063																					
11		M00064																					
12		M00075																					
13		M00076																					
14		M00080																					
15		M00112																					
16		M00115																					
17		M00117																					
18		M00118																					
19		m00119																					
20	2.8	m00124																					
21		m00128																					
22		m00139																					
23		m00140																					
24		m00142																					
25		m00144																					
26		m00146																					
27		m00147																					
28		m00149																					
29		m00152																					

30		m00153
31		m00171
32		m00192
33		m00193
34		m00195
35		m00214
36		m00233
37		m00237
38		m00239
39		m00247
40	6.0	m00248
41		m00254
42		m00260
43		m00264
44		m00267
45		m00281
46		m00283
47		m00288
48		m00289
49		m00308
50		m00309
51		m00310
52		m00311
53		m00312
54		m00321
55		m00331
56		m00344
57		m00346
58		m00372
59		m00374
60	8.6	m00405
61		m00416
62		m00417
63		m00423
64		m00429
65		m00438
66		m00458
67		m00460
68		m00461
69		m00471
70		m00472
71		m00474
72		m00490
73		m00493
74		m00494
75		m00496
76		m00500
77		m00515

78		m00539
79		m00562
80	17.3	m00567
81		m00568
82		m00569
83		m00571
84		m00572
85		m00573
86		m00585
87		m00604
88		m00606
89		m00608
90		m00609
91		m00610
92		m00612
93		m00623
94		m00636
95		m00637
96		m00638
97		m00639
98		m00641
99		m00682
100	19.8	m00690
101		m00724
102		m00730
103		m00731
104		m00735
105		m00750
106		m00752
107		m00759
108		m00760
109		m00771
110		m00773
111		m00779
112		m00782
113		m00785
114		m00787
115		m00788
116		m00800
117		m00836
118		m00844
119		m00856
120	20.3-<br>27.8	m00870
121		m00871
122		m00875
123		m00955
124		m00973
125		m00974

126		m00992
127		m00994
128		m00995
129		m00996
130		m00999
131		m01000
132		m01012
133		m01016
134		m01017
135		m01019
136		m01046
137		m01050
138		m01152
139		m01155
140	34.8-35.6	m01184
141		m01185
142		m01203
143		m01226
144		m01232
145		m01241
146		m01244
147		m01278
148		m01287
149		m01288
150		m01289
151		m01293
152		m01294
153		m01295
154		m01307
155		m01310
156		m01320
157		m01330
158		m01331
159		m01332
160	35.6-45.3	m01338
161		m01350
162		m01351
163		m01352
164		m01362
165		m01364
166		m01367
167		m01368
168		m01375
169		m01402
170		m01403
171		m01404
172		m01405

173		m01409
174		m01413
175		m01481
176		m01491
177		m01498
178		m01521
179		m01527
180	49.6-52.9	m01545
181		m01559
182		m01576
183		m01608
184		m01625
185		m01626
186		m01644
187		m01677
188		m01789
189		m01800
190		m01821
191		m01852
192		m01853
193		m01854
194		m01855
195		m01859
196		m01863
197		m01879
198		m01883
199		m01884
200	55.9-56.2	m01891
201		m01911
202		m01912
203		m01946
204		m01955
205		m01976
206		m02106
207		m02108
208		m02109
209		m02110
210		m02111
211		m02125
212		m02139
213		m02152
214		m02161
215		m02166
216		m02173
217		m02177
218		m02188
219		m02235

220	56.2-57.3	m02238
221		m02258
222		m02265
223		m02277
224		m02278
225		m02283
226		m02305
227		m02331
228		m02389
229		m02407
230		m02408
231		m02410
232		m02411
233		m02419
234		m02425
235		m02460
236		m02461
237		m02465
238		m02480
239		m02508
240	57.3	m02509
241		m02542
242		m02571
243		m02572
244		m02666
245		m02674
246		m02677
247		m02697
248		m02716
249		m02725
250		m02774
251		m02776
252		m02779
253		m02858
254		m02904
255		m02930
256		m02931
257		m02935
258		m02950
259		m02952
260	71.4-77.4	m02958
261		m02962
262		m02965
263		m02970
264		m02989
265		m02993
266		m02995

267		m02996
268		m02999
269		m03002
270		m03003
271		m03006
272		m03056
273		m03071
274		m03074
275		m03138
276		m03145
277		m03146
278		m03164
279		m03172
280	80.2-80.5	m03173
281		m03204
282		m03205
283		m03206
284		m03212
285		m03243
286		m03262
287		m03275
288		m03311
289		m03320
290		m03340
291		m03348
292		m03349
293		m03350
294		m03351
295		m03352
296		m03365
297		m03382
298		m03403
299		m03416
300	84.6-85.7	m03417
301		m03428
302		m03437
303		m03443
304		m03460
305		m03463
306		m03464
307		m03505
308		m03507
309		m03508
310		m03509
311		m03510
312		m03515
313		m03518

314		m03519
315		m03529
316		m03530
317		m03534
318		m03535
319		m03537
320	88.7-90.1	m03573
321		m03580
322		m03582
323		m03595
324		m03596
325		m03601
326		m03602
327		m03607
328		m03610
329		m03611
330		m03613
331		m03614
332		m03616
333		m03636
334		m03660
335		m03661
336		m03666
337		m03669
338		m03673
339		m03678
340	91.4-97.3	m03689
341		m03724
342		m03725
343		m03727
344		m03732
345		m03733
346		m03734
347		m03741
348		m03742
349		m03748
350		m03779
351		m03790
352		m03791
353		m03798
354		m03801
355		m03845
356		m03848
357		m03849
358		m03894
359		m03895
360	99.2-	m03899

	101.9	
361		m03902
362		m03905
363		m03917
364		m03918
365		m03919
366		m03921
367		m03924
368		m03925
369		m03965
370		m03974
371		m03976
372		m03989
373		m04094
374		m04095
375		m04119
376		m04140
377		m04141
378		m04143
379		m04144
380	112.9	m04212
381		m04225
382		m04233
383		m04234
384		m04235
385		m04239
386		m04242
387		m04285
388		m04292
389		m04306
390		m04311
391		m04329
392		m04330
393		m04367
394		m04378
395		m04420
396		m04442
397		m04443
398		m04449
399		m04456
400	116.2	m04457
401		m04458
402		m04483
403		m04489
404		m04490
405		m04495
406		m04496
407		m04497

408		m04498																					
409		m04511																					
410		m04512																					
411		m04513																					
412		m04517																					
413		m04518																					
414		m04519																					
415		m04521																					
416	117.9	m04529																					

Figure 13. Distribution of rice gene models predicted from chromosome 12 on the 21 wheat chromosomes. Serial numbers on the left represent the gene order in rice according to supporting table 12. Each of the seven homoeologous groups of wheat chromosomes has been color-coded and matches with rice genes are represented by color filled cells. The values in cM represent positions defined by genetically mapped markers in the RGP map.

			Wheat homoeologous groups																				
lo.	Rice chr 12		1			2			3			4			5			6			7		
	cM	Gene model	A	B	D	A	B	D	A	B	D	A	B	D	A	B	D	A	B	D	A	B	D
1	0.0-5.5	m00017																					
2		m00019																					
3		m00037																					
4		m00038																					
5		m00039																					
6		m00040																					
7		m00043																					
8		m00044																					
9		m00052																					
10		m00053																					
11		m00059																					
12		m00060																					
13		m00074																					
14		m00075																					
15		m00076																					
16		m00079																					
17		m00080																					
18		m00082																					
19		m00083																					
20	7.1	m00098																					
21		m00114																					
22		m00116																					
23		m00124																					
24		m00129																					
25		m00138																					
26		m00139																					
27		m00140																					
28		m00141																					
29		m00142																					
30		m00143																					

31		m00149																					
32		m00150																					
33		m00155																					
34		m00169																					
35		m00175																					
36		m00197																					
37		m00198																					
38		m00213																					
39		m00215																					
40	9.7	m00216																					
41		m00221																					
42		m00223																					
43		m00224																					
44		m00231																					
45		m00236																					
46		m00247																					
47		m00249																					
48		m00250																					
49		m00251																					
50		m00256																					
51		m00268																					
52		m00269																					
53		m00270																					
54		m00271																					
55		m00272																					
56		m00277																					
57		m00280																					
58		m00297																					
59		m00305																					
60	10.8	m00306																					
61		m00307																					
62		m00313																					
63		m00322																					
64		m00335																					
65		m00336																					
66		m00342																					
67		m00362																					
68		m00391																					
69		m00404																					
70		m00407																					
71		m00422																					
72		m00432																					
73		m00433																					
74		m00437																					
75		m00438																					
76		m00444																					
77		m00445																					
78		m00446																					

79		m00454
80	12.2-26.0	m00476
81		m00478
82		m00481
83		m00485
84		m00488
85		m00494
86		m00499
87		m00504
88		m00509
89		m00514
90		m00517
91		m00519
92		m00520
93		m00522
94		m00523
95		m00524
96		m00526
97		m00541
98		m00544
99		m00546
100	12.2-26.0	m00556
101		m00557
102		m00578
103		m00588
104		m00590
105		m00601
106		m00603
107		m00604
108		m00606
109		m00626
110		m00632
111		m00634
112		m00643
113		m00647
114		m00657
115		m00658
116		m00659
117		m00661
118		m00662
119		m00669
120	27.6	m00670
121		m00671
122		m00705
123		m00709
124		m00710
125		m00711
126		m00716

127		m00719
128		m00721
129		m00724
130		m00726
131		m00727
132		m00731
133		m00732
134		m00734
135		m00735
136		m00736
137		m00737
138		m00738
139		m00740
140	30.0	m00741
141		m00753
142		m00764
143		m00768
144		m00772
145		m00781
146		m00782
147		m00783
148		m00784
149		m00785
150		m00786
151		m00841
152		m00854
153		m00868
154		m00893
155		m00894
156		m00896
157		m00903
158		m00918
159		m00922
160	39.4	m00923
161		m00934
162		m00936
163		m00943
164		m00978
165		m00983
166		m01018
167		m01020
168		m01021
169		m01029
170		m01036
171		m01037
172		m01038
173		m01039
174		m01043

175		m01044
176		m01177
177		m01183
178		m01184
179		m01204
180	41.8-42.7	m01215
181		m01216
182		m01220
183		m01221
184		m01225
185		m01227
186		m01228
187		m01229
188		m01234
189		m01241
190		m01242
191		m01254
192		m01255
193		m01263
194		m01268
195		m01272
196		m01302
197		m01308
198		m01309
199		m01316
200	47.6-48.2	m01329
201		m01334
202		m01349
203		m01350
204		m01351
205		m01364
206		m01378
207		m01381
208		m01382
209		m01415
210		m01429
211		m01518
212		m01522
213		m01526
214		m01542
215		m01566
216		m01597
217		m01598
218		m01600
219		m01601
220	49.3	m01605
221		m01606
222		m01611

223		m01612
224		m01619
225		m01643
226		m01650
227		m01686
228		m01687
229		m01710
230		m01727
231		m01732
232		m01735
233		m01738
234		m01770
235		m01788
236		m01804
237		m01894
238		m01897
239		m01900
240	50.4-51.5	m01921
241		m01922
242		m01930
243		m01931
244		m01932
245		m01934
246		m01940
247		m01941
248		m01942
249		m02028
250		m02078
251		m02101
252		m02159
253		m02164
254		m02167
255		m02215
256		m02217
257		m02218
258		m02219
259		m02220
260	51.8	m02228
261		m02229
262		m02305
263		m02307
264		m02309
265		m02318
266		m02319
267		m02333
268		m02334
269		m02355
270		m02359

271		m02366
272		m02381
273		m02410
274		m02411
275		m02414
276		m02418
277		m02422
278		m02447
279		m02451
280	51.8-55.1	m02468
281		m02470
282		m02471
283		m02472
284		m02473
285		m02486
286		m02490
287		m02507
288		m02616
289		m02629
290		m02633
291		m02640
292		m02642
293		m02659
294		m02660
295		m02661
296		m02673
297		m02723
298		m02727
299		m02755
300	58.9-61.6	m02768
301		m02826
302		m02831
303		m02846
304		m02849
305		m02861
306		m02862
307		m02867
308		m02879
309		m02881
310		m02907
311		m02909
312		m02910
313		m02911
314		m02918
315		m02919
316		m02928
317		m02931
318		m02943

319		m02962
320	62.2-64.4	m02968
321		m02970
322		m03013
323		m03058
324		m03059
325		m03060
326		m03077
327		m03089
328		m03091
329		m03093
330		m03095
331		m03132
332		m03142
333		m03143
334		m03144
335		m03145
336		m03155
337		m03158
338		m03170
339		m03182
340	67.2	m03183
341		m03184
342		m03186
343		m03198
344		m03207
345		m03216
346		m03217
347		m03219
348		m03220
349		m03224
350		m03229
351		m03230
352		m03240
353		m03283
354		m03368
355		m03374
356		m03375
357		m03377
358		m03406
359		m03408
360	73.0	m03413
361		m03423
362		m03427
363		m03428
364		m03429
365		m03452
366		m03454

367		m03457
368		m03460
369		m03480
370		m03489
371		m03490
372		m03533
373		m03547
374		m03549
375		m03550
376		m03551
377		m03553
378		m03579
379		m03583
380	75.8-78.9	m03591
381		m03597
382		m03612
383		m03620
384		m03622
385		m03623
386		m03627
387		m03628
388		m03629
389		m03640
390		m03660
391		m03661
392		m03667
393		m03670
394		m03671
395		m03672
396		m03675
397		m03676
398		m03678
399		m03679
400	86.5	m03687
401		m03689
402		m03690
403		m03691
404		m03697
405		m03699
406		m03706
407		m03710
408		m03716
409		m03717
410		m03729
411		m03730
412		m03735
413		m03736
414		m03740

415		m03741
416		m03744
417		m03748
418		m03751
419		m03757
420	88.6	m03761
421		m03772
422		m03773
423		m03774
424		m03785
425		m03786
426		m03787
427		m03788
428		m03805
429		m03811
430		m03812
431		m03821
432		m03822
433		m03824
434		m03825
435		m03826
436		m03827
437		m03828
438		m03845
439		m03865
440	94.6	m03869
441		m03870
442		m03873
443		m03874
444		m03875
445		m03878
446		m03884
447		m03890
448		m03897
449		m03898
450		m03899
451		m03900
452		m03901
453		m03902
454		m03923
455		m03924
456		m03927
457		m03932
458		m03933
459		m03945
460	97.0	m04001
461		m04012
462		m04015

463		m04023
464		m04024
465		m04044
466		m04045
467		m04056
468		m04057
469		m04058
470		m04059
471		m04079
472		m04081
473		m04087
474		m04088
475		m04092
476		m04099
477		m04100
478		m04115
479		m04116
480	99.7-100.9	m04126
481		m04127
482		m04128
483		m04130
484		m04131
485		m04147
486		m04149
487		m04150
488		m04162
489		m04173
490		m04174
491		m04175
492		m04181
493		m04205
494		m04210
495		m04216
496		m04225
497		m04232
498		m04240
499		m04243
500	105.1-106.6	m04249
501		m04271
502		m04274
503		m04278
504		m04279
505		m04282
506		m04283
507		m04284
508		m04292
509		m04293

510		m04305
511		m04330
512		m04333
513		m04334
514		m04335
515		m04339
516		m04340
517		m04341
518		m04345
519		m04356
520	108.2	m04358
521		m04359
522		m04360
523		m04361
524		m04363
525		m04364
526		m04367
527		m04368
528		m04370
529		m04381
530		m04390
531		m04391
532		m04392
533		m04393
534		m04395
535		m04399
536		m04400
537		m04401
538		m04407
539		m04408
540	109.2	m04409
541		m04413
542		m04417
543		m04419
544		m04420
545		m04421
546		m04427
547		m04431
548		m04433
549		m04434
550		m04437
551		m04438
552	109.2	m04439
